# *CEBPD* modulates the airway smooth muscle transcriptomic response to glucocorticoids

**DOI:** 10.1186/s12931-022-02119-1

**Published:** 2022-07-28

**Authors:** Mengyuan Kan, Maoyun Sun, Xiaofeng Jiang, Avantika R. Diwadkar, Vishal Parikh, Gaoyuan Cao, Eric Gebski, William Jester, Bo Lan, Reynold A. Panettieri, Cynthia Koziol-White, Quan Lu, Blanca E. Himes

**Affiliations:** 1 Department of Biostatistics, Epidemiology and Informatics, University of Pennsylvania, 402 Blockley Hall, 423 Guardian Drive, Philadelphia, PA 19104 USA; 2 Department of Environmental Health, Harvard T.H. Chan School of Public Health, Boston, MA USA; 3 Rutgers Institute for Translational Medicine and Science, Rutgers University, New Brunswick, NJ USA

**Keywords:** Airway smooth muscle, Asthma, *CEBPD*, Inflammatory response, Glucocorticoid response, RNA-Seq, TNFα

## Abstract

**Background:**

CCAAT/Enhancer Binding Protein D (CEBPD), a pleiotropic glucocorticoid-responsive transcription factor, modulates inflammatory responses. Of relevance to asthma, expression of *CEBPD* in airway smooth muscle (ASM) increases with glucocorticoid exposure. We sought to characterize *CEBPD*-mediated transcriptomic responses to glucocorticoid exposure in ASM by measuring changes observed after knockdown of *CEBPD* and its impact on asthma-related ASM function.

**Methods:**

Primary ASM cells derived from four donors were transfected with *CEBPD* or non-targeting (NT) siRNA and exposed to vehicle control, budesonide (100 nM, 18 h), TNFα (10 ng/ml, 18 h), or both budesonide and TNFα. Subsequently, RNA-Seq was used to measure gene expression levels, and pairwise differential expression results were obtained for exposures versus vehicle and knockdown versus control conditions. Weighted gene co-expression analysis was performed to identify groups of genes with similar expression patterns across the various experimental conditions (i.e., *CEBPD* knockdown status, exposures).

**Results:**

*CEBPD* knockdown altered expression of 3037 genes under at least one exposure (q-value < 0.05). Co-expression analysis identified sets of 197, 152 and 290 genes that were correlated with *CEBPD* knockdown status, TNFα exposure status, and both, respectively. JAK-STAT signaling pathway genes, including *IL6R* and *SOCS3,* were among those influenced by both TNFα and *CEBPD* knockdown. Immunoblot assays revealed that budesonide-induced IL-6R protein expression and augmented IL-6-induced STAT3 phosphorylation levels were attenuated by *CEBPD* knockdown in ASM.

**Conclusions:**

CEBPD modulates glucocorticoid responses in ASM, in part via modulation of IL-6 receptor signaling.

**Supplementary Information:**

The online version contains supplementary material available at 10.1186/s12931-022-02119-1.

## Background

Asthma is a chronic inflammatory respiratory disease characterized by variable airflow limitation and airway hyperresponsiveness to specific environmental stimuli that affects over 22 million Americans and incurs an annual cost of $81.9 billion in the U.S. [1]. Treatment of asthma according to established guidelines includes use of inhaled glucocorticoids to control symptoms in patients with persistent asthma, and “bursts” or long-term use of oral formulations to treat exacerbations or severe forms of asthma, respectively [2]. Chronic use of glucocorticoids elicits considerable adverse effects and may alter tissue sensitivity [3]. Studies to better understand glucocorticoid responses have thus been undertaken to identify mechanisms of resistance and improve personalized treatment strategies [3].

Airway smooth muscle (ASM) is a prominent asthma-related cell type that is directly involved in airway remodeling and airway narrowing [4, 5]. In addition to reducing inflammation, glucocorticoids reduce asthma symptoms by modulating other ASM-dependent processes, including impaired bronchodilation [6], airway hyperresponsiveness [7], and increased ASM contractility [8]. In cells, glucocorticoids exert some of their effects via direct modulation of gene transcription through glucocorticoid receptor (GR) binding to DNA at glucocorticoid response elements (GREs) [3]. Some targets of glucocorticoids include TNFα-inducible pro-inflammatory genes whose expression is modulated by nuclear factor κB (NF-κB) and interferons [9, 10].

CCAAT/Enhancer Binding Proteins (C/EBPs) are a family of six transcription factors that regulate immune responses, as well as cell growth, arrest and differentiation [11]. One of these proteins, CCAAT/enhancer binding protein δ (*CEBPD*), has been linked to various conditions with altered inflammatory responses [12], including cancers [13], lipopolysaccharide-induced acute lung injury [14–16], pulmonary *Aspergillus fumigatus* conidia infection [17], atherosclerosis [18], and Alzheimer’s disease [19]. According to gene expression microarray and RNA-Seq studies, *CEBPD* expression increases with glucocorticoid exposure in ASM [20, 21]. Additionally, exposure to the glucocorticoid dexamethasone increases GR occupancy near *CEBPD* in A549 cells, suggesting that *CEBPD* is a primary glucocorticoid-responsive GR target [22]. Activation of *CEBPD* by inflammatory factors, including interleukin-6 (IL-6) and tumor necrosis factor-α (TNFα), has also been observed in a variety of tissues, indicating that the modulation of inflammation by CEBPD involves complex tissue-specific signaling pathways that may have opposing outcomes depending on cellular context [12, 13, 23]. Inflammatory cytokines such as IL-6, induce the binding of C/EBPs to promoters of acute phase genes to control their transcription [23], and this binding can be inhibited by steroids [24, 25] via the activation of GR and direct interaction between GR and C/EBPs [26].

Previously, we identified hundreds of ASM glucocorticoid-responsive genes, most of which were consistently differentially expressed in cells derived from asthma donors versus donors without asthma [20]. The greatest difference in fold change based on asthma status that we observed among these genes was for *CEBPD*, suggesting that it may contribute to differences in glucocorticoid responses in people with asthma via complex interactions with signaling pathways involving pro-inflammatory cytokines (e.g., TNFα), which are also differentially expressed in asthma. Here, we sought to characterize the effects of *CEBPD* knockdown on the ASM transcriptomic response to glucocorticoid and TNFα exposures, as well as its impact on related ASM function.

## Methods

Detailed methods are provided in the Additional file 1.

### ASM RNA-Seq library construction, sequencing and data analysis

Total RNA was extracted from ASM cells derived from four non-asthma donors that were transfected with *CEBPD* or NT siRNA, and exposed to vehicle control, the glucocorticoid budesonide (BUD) (100 nM), TNFα (10 ng/ml), or BUD + TNFα for 18 h. Stranded RNA-Seq libraries were prepared and sequenced on an Illumina HiSeq 2500 instrument. RNA-Seq data are available in the Gene Expression Omnibus (GEO) under accession GSE146017.

The RAVED pipeline was used to analyze RNA-Seq data [27]. Differential expression analysis was performed for ten pairwise comparisons: *CEBPD* siRNA versus NT siRNA under the four exposure conditions (control, BUD, TNFα, BUD + TNFα); TNFα versus control in cells transfected with NT siRNA and *CEBPD* siRNA; and BUD versus control and BUD + TNFα versus TNFα in cells transfected with NT siRNA or *CEBPD* siRNA. Genes with Benjamini–Hochberg adjusted p-values (i.e., q-values) < 0.05 were considered significant. Results of individual gene’s expression across samples were visualized as boxplots, where the line in the center represents the median value, the box spans the inter-quartile range, and the whiskers show the minimum and maximum (without outliers) of the normalized read counts.

### Weighted gene co-expression network analysis

Weighted gene co-expression network analysis (WGCNA) was performed using the WGCNA R package [28] to identify groups of genes with similar expression patterns. Correlations between the resultant groups of genes and 11 phenotype variables (based on transfection, exposure, and donor status) were obtained.

### Ontological category enrichment analysis

Overall gene set enrichment analysis (GSEA) was performed using the fgsea R package [29]. For select WGCNA co-expression groups, enrichment analysis was performed using modified Fisher’s exact tests [30]. Ontological categories with q-values < 0.05 were considered significant.

### ChIP-Seq data analysis

CEBPD-binding sites were identified using the brocade pipeline [31] applied to ChIP-Seq data from GEO entry GSE32465, which measured CEBPD binding in HepG2 and K562 cell lines [32].

### Immunoblots

ASM cells derived from 6 non-asthma donors were transfected with *CEBPD* or NT siRNA and exposed to DMSO (control), IL-6 (10 ng/ml, 30 min), BUD (100 nM, 24 h), or BUD + IL-6. Immunoblot signals were developed for CEBPD, IL-6Rα, α Tubulin, STAT3, and phosphorylated STAT3 (pSTAT3) from protein samples and changes in band intensities were assessed with paired Student’s t-tests. The ratios of signals were visualized as barplots of height equivalent to the mean across donors and error bars representing standard errors (SEs) across replicates.

### ASM traction microscopy

Primary human ASM cells from non-asthma donors were transfected with *CEBPD* or NT siRNA. Fourier transfer traction microscopy (FTTM) [33] was used to measure traction forces in cells at baseline or exposed to either the contractile agonist histamine (1 μM) or the β_2_-agonist isoproterenol (1 μM) for 5 min. Traction forces were normalized to the baseline traction and visualized as barplots of height equivalent to the mean across five donors and error bars representing SEs across replicates.

## Results

### RNA-Seq data met quality control (QC) considerations

Thirty-two ASM samples corresponding to four non-smoking donors without chronic disease, four exposure conditions and either *CEBPD* or NT siRNA transfection status were prepared. Expression of *CEBPD* in knockdown cells compared to their donor-paired NT siRNA-transfected cells was decreased an average of 67% according to RT-qPCR measurements for all but one sample that showed no change in *CEBPD* expression levels and thus, was excluded from RNA-Seq library preparation, resulting in 31 samples selected for RNA-Seq (Additional file 1: Fig. E1). These RT-qPCR measurements also showed that *CXCL8* expression increased more than tenfold with TNFα exposure, demonstrating an expected pro-inflammatory response, and the increased *CXCL8* expression was blunted by BUD, demonstrating an expected decrease in inflammatory response with glucocorticoid exposure. The RNA-Seq data for the 31 samples sequenced was deemed of high quality (Additional file 1: Fig. E2A, B, Table E1), and all samples were included in differential expression analyses. Normalized *CEBPD* read counts decreased by 70% in the knockdown cells compared to NT siRNA transfected cells (Additional file 1: Fig. E2C). In NT siRNA cells, genes differentially expressed in response to BUD exposure included well-known glucocorticoid-responsive genes (e.g., *FKBP5*, *TSC22D3*, *GLUL*, *PER1*, *CRISPLD2*) [20, 21]. Genes differentially expressed in response to TNFα exposure included well-known pro-inflammatory cytokines (e.g., *IL6, CXCL8*) [9, 10].

**Fig. 1:**
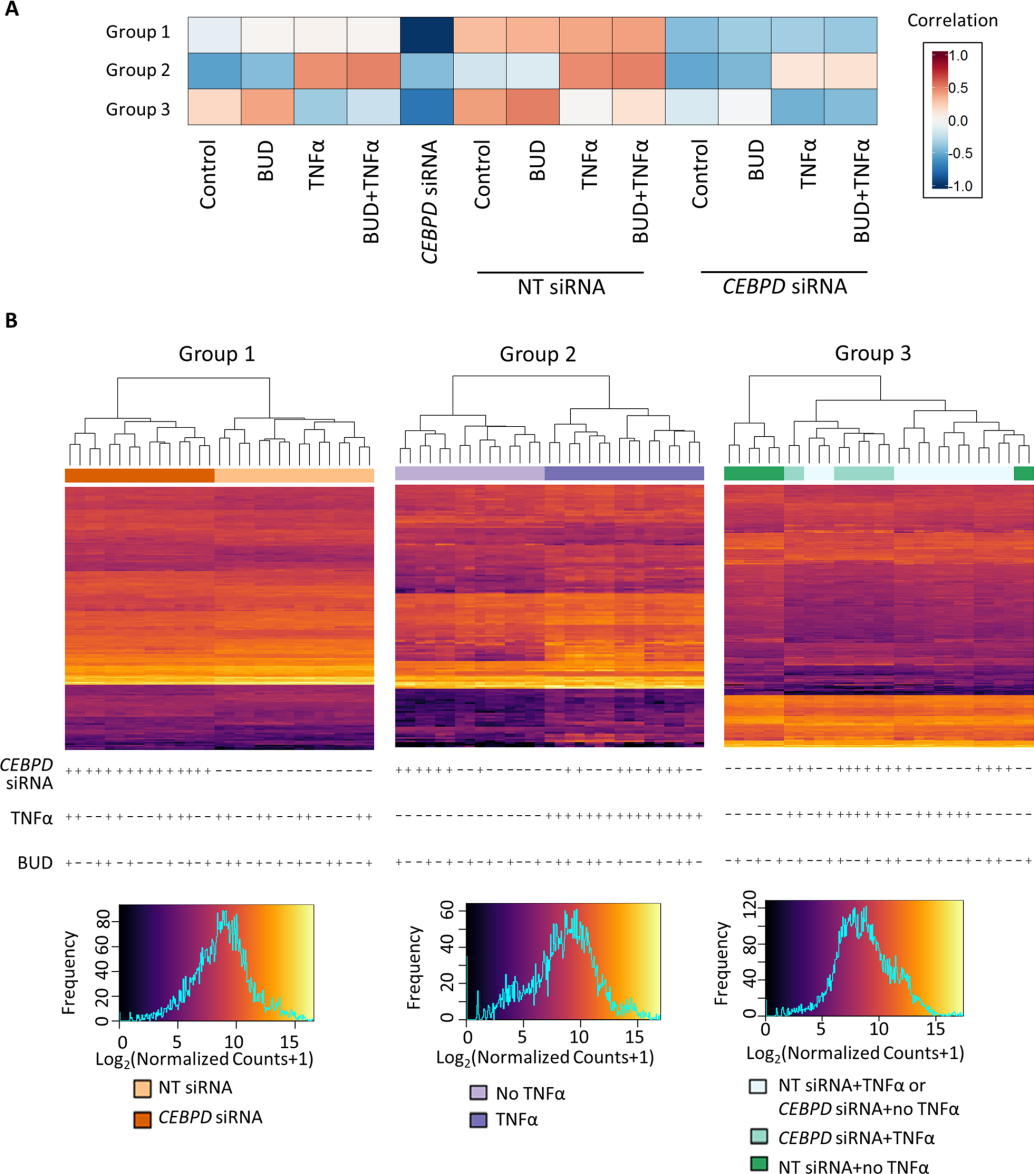
Gene co-expression groups associated with various exposures. **A** Heatmap of correlations between *eigengenes* and 13 experimental conditions in three gene co-expression groups. **B** Heatmaps of gene expression represented by log_2_(normalized count + 1) for all 31 samples in three gene co-expression groups. Samples in these group can be distinguished based on *CEBPD* siRNA status, TNFα exposure status and both. *BUD* budesonide, *NT* non-targeting

### Overall ASM transcriptomic changes in response to *CEBPD* knockdown

Comparison of *CEBPD* siRNA versus NT siRNA samples found 1,617, 1,459, 1,330 and 1,985 differentially expressed genes within control, TNFα, BUD and BUD + TNFα exposure conditions, respectively (Table 1). A total of 3037 genes were differentially expressed under at least one of these exposure conditions, and 588 of the genes were in common across the four exposure groups. The five top-ranked genes influenced by *CEBPD* knockdown according to lowest q-value for each of the four exposure conditions, representing 13 unique genes, included *TNFRSF10D*, a TNF receptor gene with an inhibitory role in apoptosis [34], whose expression was increased by *CEBPD* knockdown, and *TXNIP*, an NF-κB inhibitor gene [35] whose expression was decreased by *CEBPD* knockdown (Table 2). GSEA found that 23 ontological categories were significantly enriched in at least one exposure group with *CEBPD* knockdown (Additional file 1: Table E2, Fig. E3). Seven of these categories were shared across all exposures: *peptide chain elongation*, *ribosome*, *3' UTR mediated translational regulation*, *influenza viral RNA transcription and replication*, *nonsense mediated decay enhanced by the exon junction*, *SRP-dependent cotranslational protein targeting to membrane*, and *influenza life cycle*.

**Table 1:** Number of significantly differentially expressed genes in various comparisons

Genes whose expression differed with *CEBPD* knockdown					
*CEBPD* siRNA versus NT siRNA across various exposures					
Control	TNFα	BUD	BUD + TNFα	Overlap	Total
1617	1459	1330	1985	588	3037

Genes whose expression differed with TNFα and/or budesonide exposure				
	NT siRNA	*CEBPD* siRNA	Overlap	Total
TNFα versus control	2315	1953	1515	2753
BUD versus control	470	421	276	615
BUD + TNFα versus TNFα	535	474	264	745

*BUD* budesonide

Genes with q-value < 0.05 are considered significant

**Table 2:** Top differentially expressed genes in *CEBPD* siRNA versus NT siRNA cells under exposures of control, BUD, TNFα, or BUD + TNFα

Gene symbol (gene name)	Ensembl ID	Control				BUD				TNFα				BUD + TNFα			
		Log_2_ FC	Q-value	Mean normalized counts		Log_2_ FC	Q-value	Mean normalized counts		Log_2_ FC	Q-value	Mean normalized counts		Log_2_ FC	Q-value	Mean normalized counts	
				NT	*CEBPD*			NT	*CEBPD*			NT	*CEBPD*			NT	*CEBPD*
*CEBPD* (CCAAT/enhancer binding protein (C/EBP), delta)	ENSG00000221869	− 1.73	6.40E−54	1086	328	− 1.84	2.20E−30	1578	465	− 1.47	4.70E−14	897	322	− 1.48	2.70E−38	1139	411
*TNFRSF10D* (tumor necrosis factor receptor superfamily member 10d)	ENSG00000173530	1.47	3.40E−43	1153	3071	1.11	1.20E−21	1126	2053	1.15	8.10E−03	1074	2621	1.11	9.10E−23	918	2045
*CDKN1A* (cyclin-dependent kinase inhibitor 1A (p21, Cip1))	ENSG00000124762	1.07	6.80E−39	6008	12,477	1.30	6.20E−42	5028	10,959	1.19	4.20E−29	4853	10,939	1.21	8.30E−20	4720	10,733
*PDIA4* (protein disulfide isomerase family A member 4)	ENSG00000155660	− 1.69	2.00E−37	3825	1197	− 1.38	4.00E−33	3203	1204	− 1.61	1.30E−44	5132	1686	− 1.66	2.90E−29	4427	1412
*RPS15* (ribosomal protein S15)	ENSG00000115268	− 1.25	7.30E−31	2974	1268	− 1.15	2.00E−27	3090	1470	− 1.19	1.50E−53	2745	1201	− 1.37	3.00E−45	2945	1142
*DPP4* (dipeptidyl-peptidase 4)	ENSG00000197635	− 1.19	3.20E−26	1877	831	− 1.08	5.00E−15	1882	1124	− 1.18	2.10E−26	3342	1477	− 1.17	9.20E−35	3793	1693
*TXNIP* (thioredoxin interacting protein)	ENSG00000265972	− 1.37	4.10E−25	3320	1317	− 1.13	3.00E−18	3708	1876	− 1.36	1.40E−37	2387	952	− 1.46	2.70E−21	2544	1018
*ELN* (elastin)	ENSG00000049540	1.21	3.80E−23	7693	16,628	1.00	5.20E−04	14,062	26,557	1.46	6.70E−29	5276	12,817	1.42	6.90E−33	6815	15,927
*VGLL3* (vestigial like family member 3)	ENSG00000206538	− 1.09	1.10E−22	2351	1096	− 0.88	8.60E−09	3369	1924	− 1.00	9.80E−33	2038	1020	− 0.70	3.20E−08	2966	1832
*CPM* (carboxypeptidase M)	ENSG00000135678	− 1.28	1.30E−18	896	385	− 1.40	2.50E−18	728	237	− 1.39	5.60E−24	1053	407	− 1.26	3.60E−33	1043	440
*IGFBP5* (insulin like growth factor binding protein 5)	ENSG00000115461	− 0.58	3.50E−06	251,976	174,891	− 0.36	5.80E−01	179,541	140,387	− 1.02	3.50E−29	253,204	127,426	− 1.06	3.10E−27	165,764	78,967
*ADH1B* (alcohol dehydrogenase 1B (class I), beta polypeptide)	ENSG00000196616	− 2.06	2.70E−04	6291	1905	− 2.18	7.50E−76	11,133	2493	− 2.29	1.10E−04	2143	693	− 1.97	3.20E−05	5099	1540
*PRELP* (proline/arginine-rich end leucine-rich repeat protein)	ENSG00000188783	− 1.66	7.80E−02	500	142	− 2.35	7.60E−39	657	149	− 1.53	4.20E−02	367	116	− 1.06	3.80E−01	366	223

Top five genes with smallest q-values were selected from each comparison yielding 13 genes in total

### Influence of *CEBPD* knockdown on ASM transcriptomic response to TNFα exposure

When comparing TNFα versus control exposure, there were 2315 and 1953 differentially expressed genes in NT siRNA and *CEBPD* siRNA cells, respectively, 1515 of which were in common (Table 1). The log_2_ fold changes corresponding to the differentially expressed genes were broadly similar in NT siRNA and *CEBPD* siRNA cells (Additional file 1: Fig. E4A). The five top-ranked genes according to lowest q-value whose expression was altered by TNFα in each of the two transfection status conditions, representing seven unique genes, are shown in Table 3. Although these seven genes, which included the cytokines *IL32* and *IL6,* were significantly differentially expressed regardless of *CEBPD* knockdown status, genes such as *IER3* and *ICAM1* had reduced levels of expression with *CEBPD* knockdown and were significantly differentially expressed when comparing *CEBPD* knockdown versus NT siRNA status within TNFα-exposed cells (q-value = 7.73 × 10^–5^ for *IER3* and 5.47 × 10^–4^ for *ICAM1*)*.* GSEA found that the ontological categories overrepresented by genes in response to TNFα exposure were the same regardless of knockdown status (Additional file 1: Table E3, Fig. E5). In contrast, the ontological categories obtained for the *CEBPD* siRNA versus NT siRNA comparison among TNFα-exposed cells found that the categories *smooth muscle contraction* and *nitric oxide stimulates guanylate cyclase* were affected by *CEBPD* knockdown (Additional file 1: Table E2, Fig. E3). Individual genes that drove these differences in ontological category overrepresentation included *ITGA1* and *MYL9* for *smooth muscle contraction* (*CEBPD* siRNA versus NT siRNA q-value = 2.24 × 10^–17^ and 3.95 × 10^–8^, respectively) and *GUCY1B3* and *MRVI1* for *nitric oxide stimulates guanylate cyclase* (*CEBPD* siRNA versus NT siRNA q-value = 6.92 × 10^–3^ and 1.08 × 10^–14^, respectively) (Additional file 1: Fig. E6).

**Table 3:** Top differentially expressed genes in TNFα versus control in NT siRNA or *CEBPD* siRNA cells

Gene symbol (gene name)	Ensembl ID	NT siRNA				CEBPD siRNA			
		log_2_FC	Q-value	Mean normalized counts		log_2_FC	Q-value	Mean normalized counts	
				no TNFα	TNFα			no TNF	TNFα
*IL32* (interleukin 32)	ENSG00000008517	3.04	6.50E−118	284	2213	3.27	5.20E−189	237	2306
*IL6* (interleukin 6)	ENSG00000136244	3.57	3.10E−80	892	11,217	3.56	8.50E−97	878	10,187
*TNFAIP3* (TNF alpha induced protein 3)	ENSG00000118503	3.40	5.30E−79	901	9453	3.36	5.50E−27	916	9196
*COL7A1* (collagen, type VII, alpha 1)	ENSG00000114270	1.40	1.90E−75	3896	10,229	1.60	7.70E−58	3237	9913
*NFKB2* (nuclear factor of kappa light polypeptide gene enhancer in B-cells 2 (p49/p100))	ENSG00000077150	2.13	2.70E−70	666	2909	2.26	1.90E−86	515	2463
*IER3* (immediate early response 3)	ENSG00000137331	1.95	1.70E−50	1141	4313	1.89	3.50E−83	797	2903
*ICAM1* (intercellular adhesion molecule 1)	ENSG00000090339	3.20	2.20E−24	490	4243	3.45	1.00E−68	268	2715

Top five genes with smallest q-values were selected from each comparison yielding seven genes in total

### Influence of *CEBPD* knockdown on ASM transcriptomic response to budesonide exposure

When comparing BUD versus control exposure, there were 470 and 421 differentially expressed genes among NT siRNA and *CEBPD* siRNA samples, respectively, 276 of which were in common (Table 1). When comparing BUD + TNFα versus TNFα exposure, there were 535 and 474 differentially expressed genes in NT siRNA and *CEBPD* siRNA, respectively, 264 of which overlapped. Table 4 lists the five top-ranked genes according to q-value for BUD versus control or BUD + TNFα versus TNFα in either transfection condition, yielding 15 unique genes, which include the well-known glucocorticoid-responsive genes *GLUL* and *DUSP1* [20, 21]. Some top-ranked genes, such as *IL1B* and *PTGS2*, had expression levels that were highly induced by TNFα exposure (TNFα versus control q-values < 10^–10^) and therefore, had greater observed differences in expression with glucocorticoid exposure in the BUD + TNFα co-stimulation than the BUD condition. Overall, however, the log_2_ fold changes of the differentially expressed genes in BUD versus control and BUD + TNFα versus TNFα were broadly similar by transfection status (Additional file 1: Fig. E4B, C).

**Table 4:** Top differentially expressed genes in BUD versus control and BUD + TNFα versus TNFα in NT siRNA or *CEBPD* siRNA cells

Gene symbol (gene name)	Ensembl ID	BUD versus control in NT siRNA				BUD versus control in *CEBPD* siRNA				BUD + TNF versus TNFα in NT siRNA				BUD + TNF versus TNFα in *CEBPD* siRNA			
		Log_2_ FC	Q-value	Mean normalized counts		Log_2_ FC	Q-value	Mean normalized counts		Log_2_ FC	Q-value	Mean normalized counts		Log_2_ FC	Q-value	Mean normalized counts	
				No BUD	BUD			No BUD	BUD			No BUD	BUD			No BUD	BUD
*PER3* (period circadian clock 3)	ENSG00000049246	− 2.58	8.10E−81	602	101	− 2.26	1.00E−37	456	107	− 2.80	7.60E−34	287	43	− 2.67	1.00E−21	217	37
*ANGPTL2* (angiopoietin like 2)	ENSG00000136859	− 1.05	3.90E−33	3939	1889	− 1.20	2.70E−40	3603	1608	− 1.06	2.20E−32	1624	782	− 1.13	6.80E−24	1580	712
*GLUL* (glutamate-ammonia ligase)	ENSG00000135821	0.87	9.90E−33	3762	6921	0.58	2.00E−16	2859	4276	0.46	5.30E−08	3276	4504	0.21	5.90E−02	2671	3091
*DUSP1* (dual specificity phosphatase 1)	ENSG00000120129	0.95	8.40E−27	1279	2440	0.61	4.00E−15	2010	2801	0.60	4.30E−02	2464	3809	0.56	5.10E−09	3249	4834
*MAOA* (monoamine oxidase A)	ENSG00000189221	1.58	8.40E−27	213	600	1.82	2.20E−02	165	334	1.57	4.20E−06	275	724	1.61	1.60E−17	182	561
*ADH1B* (alcohol dehydrogenase 1B (class I), beta polypeptide)	ENSG00000196616	0.82	3.70E−14	6291	11,133	1.23	3.90E−50	1905	2493	1.40	6.50E−04	2143	5099	1.71	6.00E−03	693	1540
*MMP10* (matrix metallopeptidase 10)	ENSG00000166670	− 1.12	1.90E−13	615	313	− 0.71	9.70E−03	620	140	− 1.17	6.00E−22	1457	665	− 1.10	3.90E−33	1911	901
*NRG1* (neuregulin 1)	ENSG00000157168	− 0.94	1.30E−12	1095	538	− 0.85	1.40E−10	1476	416	− 1.18	1.00E−28	2334	1111	− 1.03	7.70E−46	3254	1626
*NR1D2* (nuclear receptor subfamily 1 group D member 2)	ENSG00000174738	− 0.75	3.80E−11	1871	1116	− 0.50	3.90E−08	1888	1250	− 1.06	1.70E−26	1572	743	− 0.74	4.90E−14	1493	888
*GABBR2* (gamma-aminobutyric acid (GABA) B receptor, 2)	ENSG00000136928	0.86	4.00E−11	427	739	1.16	3.50E−02	529	868	1.20	4.30E−21	393	854	1.50	6.30E−30	482	1294
*PTX3* (pentraxin 3)	ENSG00000163661	0.69	1.50E−10	1778	2645	0.81	2.10E−02	1729	3765	1.24	7.10E−36	3294	8292	0.83	1.90E−25	3512	6464
*KLF9* (Kruppel-like factor 9)	ENSG00000119138	0.75	2.50E−10	1692	2736	0.91	5.40E−29	1694	2361	0.55	1.40E−08	1472	2142	0.94	2.30E−13	1680	3156
*MMP1* (matrix metallopeptidase 1)	ENSG00000196611	− 0.93	1.90E−06	19,142	10,575	− 0.88	1.80E−36	22,018	11,185	− 0.84	8.40E−14	78,174	47,428	− 1.00	6.50E−25	70,242	37,456
*IL1B* (interleukin 1 beta)	ENSG00000125538	− 1.05	2.50E−02	68	35	− 1.80	8.00E−06	97	35	− 1.54	1.60E−05	911	349	− 1.62	5.20E−34	1217	454
*PTGS2* (prostaglandin-endoperoxide synthase 2 (prostaglandin G/H synthase and cyclooxygenase))	ENSG00000073756	− 0.83	1.20E−01	946	565	− 0.85	1.30E−12	928	549	− 1.78	2.00E−15	5682	1671	− 1.30	2.00E−48	6348	2738

Top five genes with smallest q-values were selected from each comparison yielding 15 genes in total

We found that 23 ontological categories were significantly enriched in at least one of the four comparisons involving BUD (i.e., BUD versus control in: (1) NT siRNA, and (2) *CEBPD* siRNA samples; and BUD + TNFα versus TNFα in: (3) NT siRNA, and (4) *CEBPD* siRNA samples; Additional file 1: Table E4 and Fig. E7). Six of these categories were shared across the four comparisons, including *smooth muscle contraction*, suggesting that contraction-related gene expression changes were influenced by BUD regardless of transfection or TNFα co-stimulation status. In contrast, some categories were enriched only under conditions that also involved *CEBPD* knockdown and/or TNFα exposure. For example, the *cytokine-cytokine receptor interaction* category was not enriched in the BUD versus control within NT siRNA condition, but it was enriched in the BUD versus control within *CEBPD* knockdown and/or TNFα exposure conditions due to the differential expression of genes such as *IL6*, *IL1A*, and *IL1B* observed under the latter conditions. An example of a category that was enriched only with co-stimulation of TNFα was *extracellular matrix organization*, which was driven by the collagen-related genes *COL12A1*, *COL7A1*, *COL5A3* and *COL13A1* that were differentially expressed with TNFα + BUD exposure but not BUD alone.

### Identification of gene co-expression groups and their association with *CEBPD* knockdown and TNFα exposure status

We selected the 1,365 genes that were differentially expressed with *CEBPD* knockdown in any exposure condition for WGCNA analysis. Soft-thresholding power (β) of 18 was chosen to generate an unsigned weighted co-expression network (Additional file 1: Fig. E8). Of eight groups of co-expressed genes identified, three that were significantly correlated with *CEBPD* knockdown or exposure status, but not donor status, were considered further (Additional file 1: Fig. E9). Correlation coefficients showed that Group 1 (composed of 197 genes) was correlated with *CEBPD* knockdown status only, Group 2 (composed of 152 genes) was correlated with TNFα exposure status, and Group 3 (composed of 290 genes) was correlated with both *CEBPD* knockdown and TNFα exposure status (Fig. 1A). To a lesser extent, Groups 2 and 3 were correlated with BUD exposure (p < 0.05). Hierarchical clustering using gene expression levels (i.e., log_2_(normalized counts + 1)) of the genes in each co-expression group clustered the 31 samples according to their transfection and/or TNFα exposure status, consistent with phenotypes that they were associated with (Fig. 1B). Group 3 subjects were not perfectly clustered, which may be due to the slight correlation between their *eigengenes* and donor status. In terms of ontological categories overrepresented by the *eigengenes*, Group 1 contained genes involved in *regulation of actin cytoskeleton*; Group 2 contained genes involved in *interferon*, *cytokine-cytokine*, and *JAK-STAT signaling*; and Group 3 contained genes involved in *translation*, *influenza viral RNA transcription and replication*, and *JAK-STAT signaling* (Additional file 1: Table E5).

### JAK-STAT signaling pathway genes co-expressed in response to both *CEBPD* knockdown and TNFα exposure

**Fig. 2:**
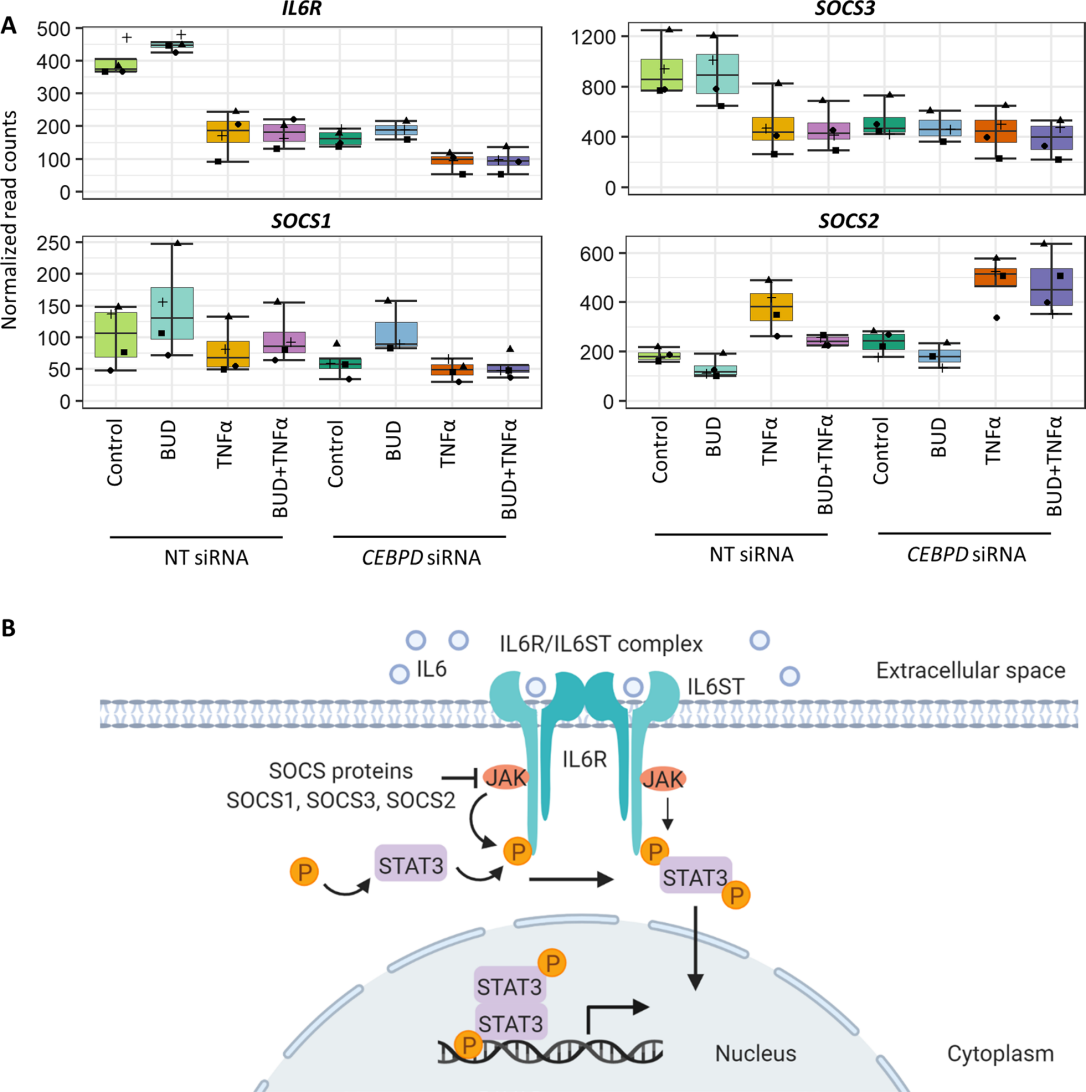
*CEBPD* knockdown influenced ASM expression of genes in the IL-6 receptor signaling pathway. **A** Expression levels of *IL6R*, *SOCS3*, *SOCS1*, and *SOCS2* in response to control, BUD, TNFα, and BUD + TNFα exposures in NT siRNA and *CEBPD* siRNA transfected cells obtained with RNA-Seq data show that CEBPD knockdown altered their response to BUD, TNFα and/or BUD + TNFα exposure. Boxplots show the median value at the center line, the box spans the inter-quartile range, and the whiskers span the minimum and maximum (without outliers) of normalized read counts (N = 3–4 donors per condition). Individual read count values are displayed as points. **B** IL-6 receptor signaling pathway diagram indicating known relationships among IL-6R, SOCS and STAT3 proteins. *BUD* budesonide, *NT* non-targeting

The Group 2 (TNFα exposure-associated) *JAK-STAT signaling* pathway genes that were overrepresented included *IL10RB, IL13RA2, IL15RA, IL7R, LEP, STAT1, STAT4*, while the Group 3 (*CEBPD* knockdown- and TNFα exposure-associated) *JAK-STAT signaling* pathway genes included *CCND3, IL24, IL6R, LIF, PIM1, SOCS1, SOCS2, SOCS3, SPRY4.* We focused further on the *JAK-STAT signaling* pathway genes in Group 3 because its co-expression patterns were associated with a combined phenotype that most relates to the influence of CEBPD on asthma glucocorticoid responses. RNA-Seq results for *IL6R*, *SOCS3*, *SOCS1* and *SOCS2—*genes known to participate in IL-6 signaling pathways—across all conditions showed that their expression levels differed with TNFα exposure versus control within the NT siRNA samples differently than within the *CEBPD* siRNA samples, consistent with the Group 3 phenotype (Fig. 2; Additional file 1: Table E6). Analysis of a ChIP-Seq dataset involving HepG2 and K562 cells found putative CEBPD-binding sites near the transcription start sites (TSS) of each of these four genes, suggesting that CEBPD can directly modulate their transcription (Additional file 1: Fig. E10).

### *CEBPD* knockdown blunted IL6-induced IL-6R signaling in ASM

**Fig. 3:**
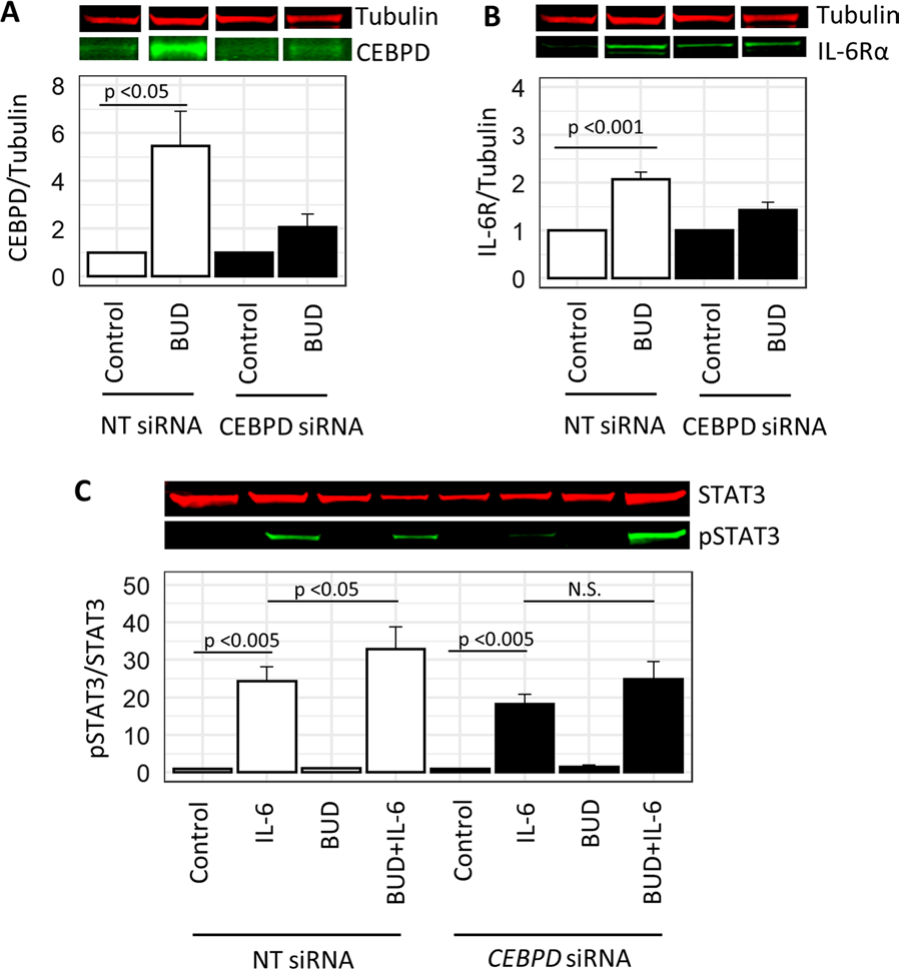
*CEBPD* knockdown blunted IL-6 receptor protein expression and signaling in ASM. **A** Representative immunoblot lanes for tubulin and CEBPD expression. Immunoblot band density quantification results for CEBPD (normalized to tubulin under NT siRNA control or *CEBPD* siRNA control within groups) show that its levels were induced by BUD exposure among NT siRNA-transfected samples, an effect that was substantially reduced with *CEBPD* siRNA transfection. **B** Representative immunoblot lanes for tubulin and IL-6R expression. Immunoblot band density quantification results for IL-6R (normalized to tubulin under NT siRNA control or *CEBPD* siRNA control within groups) were significantly increased with BUD versus control exposure among NT siRNA-transfected cells, an effect that not was significant for BUD versus control exposures with *CEBPD* knockdown. **C** Representative immunoblot results examining pSTAT3 normalized to total STAT3 expression under NT siRNA control or *CEBPD* siRNA control within groups. pSTAT3 was induced with IL-6 exposure and further augmented with addition of BUD exposure in NT siRNA transfected cells. The augmented pSTAT3 induced by BUD + IL-6 versus IL-6 was no longer significant with *CEBPD* knockdown. Barplots of signal ratios are of height equivalent to the mean across donors, and the error bars represent standard errors (SEs) across replicates with N = 6 donors per condition. Intact immunoblot bands are provided in Additional file 1: Fig. E11. *BUD* budesonide, *NT* non-targeting, *pSTAT3* phosphorylated STAT3

Given that *IL-6R* had the strongest effect among the IL-6 signaling genes with *CEBPD* knockdown according to RNA-Seq data, we sought to determine the role of *CEBPD* knockdown on IL-6-induced IL-6R signaling pathways by measuring changes of IL-6R protein levels and downstream phosphorylation of STAT3 (Fig. 2B). The *CEBPD* siRNA-transfected cells had decreased levels of CEBPD under control exposure relative to NT siRNA samples, a difference that was starker with the BUD exposure induction of CEPBD, suggesting that the knockdown effectively reduced CEBPD protein levels (Additional file 1: Fig. E11). The increase in CEBPD that was elicited with BUD exposure in NT siRNA-transfected cells (p < 0.05) was substantially diminished with CEBPD knockdown (Fig. 3A). In NT siRNA-transfected cells, IL-6R protein levels were significantly increased with BUD versus control exposure (p < 0.001), a change that was abrogated with *CEBPD* knockdown (Fig. 3B). The expected IL-6R-mediated induction of pSTAT3 by IL-6 was observed in both NT and *CEBPD* siRNA transfected cells (p-value < 0.005), and while BUD exposure further augmented pSTAT3 levels in NT siRNA transfected cells (p-value < 0.05), this effect was reduced with *CEBPD* knockdown (Fig. 3C).

### CEBPD influenced baseline ASM contractility

**Fig. 4:**
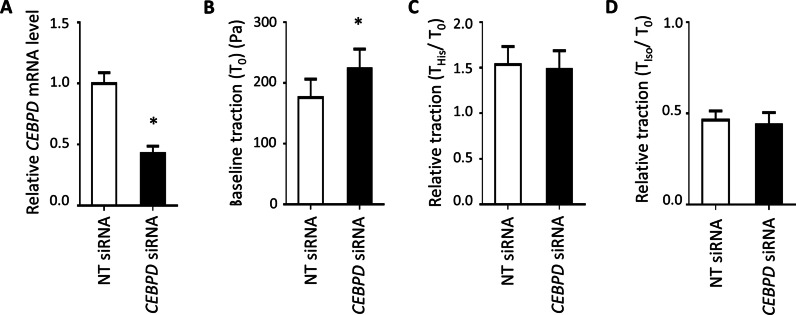
*CEBPD* influenced baseline ASM contractility. **A** *CEBPD* expression reduction in ASM was confirmed using qPCR 48 h after siRNA transfection. **B** *CEBPD* knockdown in ASM cells led to a statistically significant higher traction at baseline (T_0_). *CEBPD* knockdown in ASM cells did not alter **C** the contractile response to histamine (His) or **D** the relaxation response to isoproterenol (Iso) based on the relative traction values. Barplots are of height equivalent to the mean across five donors, and error bars represent standard errors (SEs) across replicates with N = 5 donors per condition. *p-value < 0.05. *NT* non-targeting

*CEBPD* siRNA transfection efficiency in ASM cells was confirmed to be 58% for this experiment (Fig. 4A). At baseline, the average cell traction force was significantly higher (p-value < 0.05) in *CEBPD* knockdown cells (224.4 ± 31.6 Pa, mean ± standard error hereafter) compared to cells transfected with NT siRNA (176.1 ± 30.3 Pa) (Fig. 4B). ASM traction was increased by histamine (contractile agonist) and decreased by isoproterenol (β_2_ agonist) relative to baseline levels, but these responses did not significantly differ with *CEBPD* knockdown (Fig. 4C, D).

## Discussion

We and others have observed a large number of glucocorticoid-responsive genes in ASM cells [20, 21, 36, 37]. Our most recent study found that *CEBPD* had the largest difference in glucocorticoid-induced expression changes in ASM from non-asthma donors versus fatal asthma donors (log_2_ fold change of 1.43 versus 0.48 with budesonide exposure) [20], suggesting that differing levels of *CEBPD* expression may influence glucocorticoid responsiveness in people with asthma. Our present study characterized the ASM transcriptomic responses to glucocorticoid and TNFα exposures in the context of *CEBPD* knockdown, which involved performing 10 pairwise differential expression comparisons. Due to the large number of differentially expressed genes observed among these many comparisons, we performed a weighted gene co-expression analysis to identify the groups of genes specifically changed in response to *CEBPD* knockdown in the context of other relevant exposures, thereby facilitating the identification of individual genes and pathways for validation studies of our main trait of interest.

Pairwise differential expression results revealed many changes in ASM with *CEBPD* knockdown, a large proportion of which were specific to exposure conditions. Our pathway-level results of these exposures recapitulated known pathways, including that TNFα is involved in innate immunity and interferon signaling, and that glucocorticoids alter cytokine-cytokine receptor signaling and smooth muscle contraction. The large number of differentially expressed genes observed in *CEBPD* siRNA versus NT siRNA transfected samples under glucocorticoid and/or TNFα exposures included expected findings, such as changes in genes related to the ontological categories *interferon signaling* and *downstream signaling events of B cell receptor*, as well as novel findings of relevance to asthma, such as the alteration of genes involved in *smooth muscle contraction* and *nitric oxide stimulates guanylate cyclase*. Although many genes from among these comparisons are of interest to better understand asthma, we proceeded with WGCNA to focus on groups of genes with similar changes across exposures and transfection status.

WGCNA identifies gene co-expression groups based on their topological similarity across samples, and it is able to identify relationships of these co-expression groups with multiple phenotypes under consideration [28]. We tailored WGCNA to our study goals by (1) including differentially expressed genes from the *CEBPD* siRNA versus NT siRNA across the four exposure comparisons, and (2) constructing networks that included connections regardless of the direction of expression changed by *CEBPD* knockdown (i.e., we used an unsigned correlation network). Although use of a selected set of genes biases the identification of gene co-expression groups, in this case, it allowed us to identify three salient gene co-expression groups with expression patterns corresponding to *CEBPD* knockdown and/or TNFα exposure status. We verified that individual genes within the groups had results consistent with their grouping: *CEBPD* was among the genes in Group 1 (*CEBPD* knockdown-associated) and many cytokine-related genes were among the genes in Group 2 (TNFα exposure-associated). Interestingly, TNFα exposure and *CEBPD* knockdown resulted in greater transcriptomic effects than budesonide: (1) there were substantially more differentially expressed genes identified in the pairwise TNFα versus control or *CEBPD* siRNA versus NT siRNA, than BUD versus control conditions, and (2) none of the gene co-expression groups were highly correlated with budesonide exposure status. Therefore, Group 3 was deemed most relevant to our question of understanding the impact of CEBPD on asthma-related gene expression changes. The Group 3 (*CEBPD* knockdown- and TNFα exposure-associated) JAK-STAT pathway genes included some whose expression was decreased with *CEBPD* knockdown (e.g., *IL6R*, *SOCS3*, *SOCS1*) and some whose expression changed in the opposite direction (e.g., *SOCS2*), demonstrating that WGCNA was helpful to identify sets of genes that changed under specific conditions, regardless of the direction of this change. Comparison of JAK-STAT pathway genes in Group 3 versus Group 2 was helpful to identify the TNFα-modulated ones that were also changed by *CEBPD* knockdown, which led us to select the IL-6R pathway for further study.

Consistent with RNA-Seq results showing that *IL-6R* transcript levels were substantially reduced with *CEBPD* knockdown in the BUD versus control exposure comparison, immunoblot results showed that *CEBPD* knockdown resulted in decreased IL-6R protein expression when comparing BUD versus control exposures. Extension of RNA-Seq results to the protein level also revealed that IL-6 receptor signaling vis-à-vis IL-6-induced pSTAT3 expression remained intact, although the fold-change of pSTAT3 induced with BUD + IL-6 versus IL-6 alone was only statistically significantly different among NT siRNA-transfected cells, suggesting an overall reduction of IL-6R signaling with CEBPD knockdown. Future studies are needed to investigate more detailed mechanisms whereby altered CEBPD expression and its posttranslational modifications affect IL-6R signaling in ASM to influence glucocorticoid responses in asthma.

Smooth muscle contraction pathway genes were enriched in (1) genes differentially expressed with *CEBPD* knockdown under the condition of TNFα exposure and (2) genes differentially expressed with budesonide exposure regardless of *CEBPD* knockdown status. The traction microscopy results support a potential modest effect of *CEBPD* knockdown on ASM contractile force at baseline, however, *CEBPD* had little effect on ASM excitation–contraction coupling. Together, these results suggest that *CEBPD* alone is not likely to directly regulate ASM contractility in response to glucocorticoid exposure.

Several limitations of our study are worth noting. First, we did not determine whether CEBPD modulated IL6 signaling via membrane-bound IL-6R or trans-signaling of its soluble form. Because prior studies found that membrane-bound IL-6R was not present in ASM, while its soluble form was [38], it is likely that CEBPD influences the IL-6 pathway via trans-signaling. Of note, a specific asthma phenotype has been proposed to correspond to IL-6 trans-signaling, as patients with increased IL-6 trans-signaling had more exacerbations, eosinophilia, and submucosal T cells and macrophages [39], and a coding genetic polymorphism in the IL-6R gene that promotes trans-signaling has been linked to lung function differences in people with severe asthma [40]. Second, additional experiments are necessary to determine whether some of the transcriptomic effects observed may have resulted from direct protein–protein interactions among CEBPD, NF-κB and GR. Third, our statistically significant findings for the effect of CEBPD on IL-6R and pSTAT3 had modest effect sizes, which may be due to relatively long exposure times and resulting compensation by other C/EBP family members. Additional experiments are necessary to determine the time courses of CEBPD effects, as well as the concomitant role of CEBPA, CEBPB, and related proteins on IL-6 signaling.

In summary, we found that *CEBPD* knockdown resulted in many ASM transcriptomic changes in response to glucocorticoid and TNFα exposures. Among these, *CEBPD* knockdown influenced expression of several TNFα-induced JAK-STAT pathway genes, including the IL-6 receptor. Further mechanistic insights regarding these *CEBPD*-mediated ASM transcriptomic changes may lead to an improved understanding of glucocorticoid responses in patients with asthma.

## Supplementary Information

**Additional file 1: Table E1**. RNA-Seq Quality Control Metrics. **Table E2.** Gene set enrichment analysis results corresponding to the CEBPD siRNA versus NT siRNA comparisons. **Table E3**. Gene set enrichment analysis results corresponding to the TNFα versus control comparisons. **Table E4**. Gene set enrichment analysis results corresponding to budesonide exposure. **Table E5**. Ontological categories enriched within gene co-expression groups. **Table E6**. RNA-Seq differential expression results for IL6R, SOCS3, SOCS1, and SOCS2 across the 10 comparisons made. **Figure E1**. Sample quality control prior to RNA-Seq via RT-qPCR of CEBPD and CXCL8. **Figure E2**. RNA-Seq data quality control. **Figure E3**. Significant gene set enrichment analysis categories corresponding to the CEBPD siRNA versus NT siRNA comparisons. **Figure E4**. Overall RNA-Seq results for each exposure were generally consistent with CEBPD knockdown. **Figure E5**. Significant gene set enrichment analysis categories corresponding to the TNFα versus control comparisons. **Figure E6**. TNFα-responsive genes whose expression changed with CEBPD knockdown selected from two significantly changed ontological categories. **Figure E7**. Significant gene set enrichment analysis categories corresponding to budesonide-responsive genes. **Figure E8**. Selection of soft-thresholding power (β) for weighted gene co-expression network analysis. **Figure E9**. Correlations between gene co-expression groups and phenotypes. **Figure E10**. CEBPD-binding sites near select IL-6 signaling pathway genes. **Figure E11**. Full representative immunoblots and CEBPD/Tubulin expression levels showing adequacy of CEBPD knockdown.

## Data Availability

RNA-Seq data are available in the Gene Expression Omnibus (GEO) (https://www.ncbi.nlm.nih.gov/geo/) under accession GSE146017.

## Abbreviations

ASM: Airway smooth muscle
BUD: Budesonide
CEBPD: CCAAT/enhancer binding protein D
ELISA: Enzyme-linked immunosorbent assay
FTTM: Fourier transfer traction microscopy
GSEA: Gene set enrichment analysis
GR: Glucocorticoid receptor
GRE: Glucocorticoid response element
IHC: Immunohistochemistry
NF-κB: Nuclear factor κB
NES: Normalized enrichment score
NT: Non-targeting
TSS: Transcription start site
WGCNA: Weighted gene co-expression network analysis
