# Supplementary material for: CEBPD modulates the airway smooth muscle transcriptomic response to glucocorticoids
Source: Respir Res. 2022 Jul 28;23:193. doi: 10.1186/s12931-022-02119-1 (PMC9331514; doi:10.1186/s12931-022-02119-1)
Supplement: Supplementary file 1 — Additional file 1: Table E1. RNA-Seq Quality Control Metrics. Table E2. Gene set enrichment analysis results corresponding to the CEBPD siRNA versus NT siRNA comparisons. Table E3. Gene set enrichment analysis results corresponding to the TNFα versus control comparisons. Table E4. Gene set enrichment analysis results corresponding to budesonide exposure. Table E5. Ontological categories enriched within gene co-expression groups. Table E6. RNA-Seq differential expression results for IL6R, SOCS3, SOCS1, and SOCS2 across the 10 comparisons made. Figure E1. Sample quality control prior to RNA-Seq via RT-qPCR of CEBPD and CXCL8. Figure E2. RNA-Seq data quality control. Figure E3. Significant gene set enrichment analysis categories corresponding to the CEBPD siRNA versus NT siRNA comparisons. Figure E4. Overall RNA-Seq results for each exposure were generally consistent with CEBPD knockdown. Figure E5. Significant gene set enrichment analysis categories corresponding to the TNFα versus control comparisons. Figure E6. TNFα-responsive genes whose expression changed with CEBPD knockdown selected from two significantly changed ontological categories. Figure E7. Significant gene set enrichment analysis categories corresponding to budesonide-responsive genes. Figure E8. Selection of soft-thresholding power (β) for weighted gene co-expression network analysis. Figure E9. Correlations between gene co-expression groups and phenotypes. Figure E10. CEBPD-binding sites near select IL-6 signaling pathway genes. Figure E11. Full representative immunoblots and CEBPD/Tubulin expression levels showing adequacy of CEBPD knockdown. [file 12931_2022_2119_MOESM1_ESM.docx]

# Online Supplement

**Table of Contents**

[Detailed Methods 3](#_Toc107605473)

[Detailed RNA-Seq Quality Control (QC) Results 12](#_Toc107605474)

Supplementary Tables

[Table E1. RNA-Seq Quality Control Metrics. 14](#_Toc107605475)

[Table E2. Gene set enrichment analysis results corresponding to the CEBPD siRNA versus NT siRNA comparisons. 16](#_Toc107605476)

[Table E3. Gene set enrichment analysis results corresponding to the TNFα versus control comparisons. 23](#_Toc107605477)

[Table E4. Gene set enrichment analysis results corresponding to budesonide exposure. 29](#_Toc107605478)

[Table E5. Ontological categories enriched within gene co-expression groups. 38](#_Toc107605479)

[Table E6. RNA-Seq differential expression results for *IL6R*, *SOCS3*, *SOCS1*, and *SOCS2* across the 10 comparisons made. 40](#_Toc107605480)

Supplementary Figures

[Figure E1. Sample quality control prior to RNA-Seq via RT-qPCR of](#_Toc107605481) *[CEBPD](#_Toc107605481)* [and](#_Toc107605481) *[CXCL8](#_Toc107605481)*[.](#_Toc107605481)

[42](#_Toc107605481)

[Figure E2. RNA-Seq data quality control. 43](#_Toc107605482)

[Figure E3. Significant gene set enrichment analysis categories corresponding to the *CEBPD* siRNA versus NT siRNA comparisons. 44](#_Toc107605483)

[Figure E4. Overall RNA-Seq results for each exposure were generally consistent with *CEBPD* knockdown. 45](#_Toc107605484)

[Figure E5. Significant gene set enrichment analysis categories corresponding to the TNFα versus control comparisons. 46](#_Toc107605485)

[Figure E6. TNFα-responsive genes whose expression changed with *CEBPD* knockdown selected from two significantly changed ontological categories. 47](#_Toc107605486)

[Figure E7. Significant gene set enrichment analysis categories corresponding to budesonide-responsive genes. 48](#_Toc107605487)

[Figure E8. Selection of soft-thresholding power (β) for weighted gene co-expression network analysis.. 49](#_Toc107605488)

[Figure E9. Correlations between gene co-expression groups and phenotypes. 50](#_Toc107605489)

[Figure E10. CEBPD-binding sites near select IL-6 signaling pathway genes. 51](#_Toc107605490)

[Figure E11. Full representative immunoblots and CEBPD/Tubulin expression levels showing adequacy of CEBPD knockdown.](#_Toc107605491)

[52](#_Toc107605491)

[References. 53](#_Toc107605492)

# Detailed Methods

## Ethics Statement

The lung tissue from which airway smooth muscle (ASM) was derived was provided by the National Disease Research Interchange and the International Institute for the Advancement of Medicine and is obtained commercially from deceased anonymous donors. The University of Pennsylvania Committee on Studies Involving Human Beings, the Rutgers Biomedical and Health Sciences Institutional Review Board, and the Harvard Institutional Review Board have determined that use of these cells does not constitute Human Subjects research since all donor tissue was harvested anonymously and de-identified.

## Primary ASM Cell Culture, Transfection and Exposures for RNA-Seq

Primary ASM cells from four donors (non-smokers without chronic disease; 2 male, 2 female) were maintained and cultured in Ham’s F12 medium (Life technologies, Gaithersburg, MD) supplemented with 10% FBS, 50 units/mL penicillin, and 100μg/mL streptomycin. Early-passage ASM cells were seeded in 24-well plates (1.5×10^4^ cells per well). The following day, cells were transfected with 25pmol of *CEBPD* or non-targeting (NT) siRNA. Transfection of *CEBPD* siRNA and NT siRNA ON-TARGETplus pools (GE Healthcare Dharmacon, Lafayette, CO) was performed using DharmaFECT 1 reagent according to the recommended protocol from the manufacturer. After transfection for three days, cells were exposed to: 1) vehicle control, 2) the glucocorticoid budesonide (BUD) (100nM, 18hr), 3) TNFα (10ng/ml, R&D systems, Minneapolis, MN, 18hr), or 4) BUD+TNFα. Subsequently, cells were harvested and total RNA was extracted with the miRNAeasy kit (Qiagen, Valencia, CA). To verify that treatments and knockdown were successful, RNA was reverse transcribed with the miRScript II kit (Qiagen, Valencia, CA) and *CEBPD* and *CXCL8* expression for samples corresponding to the four donors were each detected in triplicate by qPCR.

## RNA-Seq Library Construction and Sequencing

Approximately 1µg of total RNA from each sample was extracted using the miRNAeasy mini kit (Qiagen, Valencia, CA) and poly(A)-selected stranded RNA-Seq libraries were prepared with the Kapa mRNA HyperPrep kit (Kapa Biosystems, Wilmington, MA), where the reverse transcript-PCR primer has the same sequence as the 3’ adaptor ligated to mRNA thus only the first-strand cDNA can be synthesized in the library. Amplified cDNA fragments were analyzed using the 2100 Bioanalyzer (Agilent Technologies, Inc., Santa Clara, CA) to determine fragment quality and size. Library concentrations were determined by Qubit Fluorometric Quantitation (Life Technologies Corporation, Carlsbad, CA). An Illumina HiSeq 2500 instrument was used to generate 125bp paired-end reads at the Bauer Core Facility of Harvard University (Cambridge, MA). RNA-Seq data are available in the Gene Expression Omnibus (GEO) under accession GSE146017.

## RNA-Seq Data Analysis

The RAVED pipeline was used to analyze RNA-Seq data and generate HTML summary reports (<https://github.com/HimesGroup/raved>) [1]. This pipeline included trimming of adapter sequences in raw reads with Trimmomatic (v.0.32) [2], and obtaining overall QC metrics with FastQC (v.0.11.7) [3]. Subsequently, trimmed reads for each sample were aligned to the reference hg38 genome using STAR (v.2.5.2b) [4]. Bamtools (v.2.3.0) [5] was used to summarize the number of mapped reads, including junction spanning reads. The Picard Tools (v.1.96; <http://picard.sourceforge.net>) RnaSeqMetrics function was used to compute the number of bases assigned to various classes of RNA, according to an hg38 refFlat file obtained via a UCSC Genome Table. HTSeq (v.0.6.1, with the strand setting ‘-s reverse’) [6] was used to quantify gene-level read counts, using hg38 human transcriptome files as reference. Raw read plots were created by displaying bigwig files for each sample in the UCSC Genome Browser. Gene-level differential expression analysis was performed under a negative binomial distribution model with DESeq2 (v.1.18.1) [7], after filtering genes with a total read count <10 and disabling the default option for independent filtering of differential expression results.

Differential expression analysis for 10 pair-wise comparisons included: *CEBPD* siRNA versus NT siRNA under 1) control, 2) BUD, 3) TNFα and 4) BUD+TNFα exposure conditions to identify the common set of genes whose expression was altered by *CEBPD*; cells exposed to TNFα versus control in 1) NT siRNA and 2) *CEBPD* siRNA to identify TNFα-induced genes whose expression was altered by *CEBPD* knockdown; cells exposed to BUD versus control in 1) NT siRNA and 2) *CEBPD* siRNA to identify glucocorticoid-responsive genes whose expression was altered by *CEBPD* knockdown; and BUD+TNFα versus TNFα in 1) NT siRNA and 2) *CEBPD* siRNA to identify glucocorticoid-responsive genes under TNFα exposure whose expression was altered by *CEBPD* knockdown. Cell donor was used as a covariate in differential expression analyses. Ensembl gene IDs (2016 archive) were annotated to official gene symbols using BioMart (v.2.34.2) [8]. The Benjamini-Hochberg approach was used to correct for multiple comparisons of genes and adjusted p-values (i.e. q-values) <0.05 were considered significant. Results of individual gene’s expression across samples were visualized as boxplots, where the line in the center represents the median value, the box spans the inter-quartile range, and the whiskers show the minimum and maximum (without outliers) of the normalized read counts.

## Weighted Gene Co-expression Network Analysis

Weighted gene co-expression network analysis was performed using the WGCNA R package (v.1.66) [9] to identify groups of genes with similar expression patterns in response to specific exposures. Genes with q-value <0.05 and an absolute log_2_ fold change >0.05 in the comparison between *CEBPD* siRNA versus NT siRNA under any of the four exposures (i.e., control, BUD, TNFα, and BUD+TNFα) were selected for gene co-expression network analysis. A gene expression matrix of selected genes was obtained and converted using log_2_(normalized counts+1). Phenotypes used to test correlation with gene co-expression groups included 1) the four exposures (i.e., control, BUD, TNFα, and BUD+TNFα), 2) two siRNA transfection status (i.e. *CEBPD* siRNA and NT siRNA), 3) eight pairwise combinations of exposure and siRNA transfection status, and 4) four donors. Phenotypes were coded as indicator variables that yielded 11 total phenotypes.

The connection strength between genes was estimated using an unsigned weighted network *adjacency* (α), i.e., by rising Pearson’s correlation coefficients (s_ij_) to a power β: $\alpha_{ij}={|S_{ij}|}^{\beta}(\beta\geq1)$ for gene i and j, where β was selected using the scale-free topology criterion [10]. The unsigned correlation network was constructed based on the assumption that negatively correlated genes have the same connection strength as the positively correlated ones. Network connectivity of the gene i (k_i_) was computed as the sum of the *adjacency* matrix with all the other genes (u) in the network: $k_{i}= \sum_{u\neq i} \alpha_{iu}$. The *topological overlap matrix* (TOM) between genes i and j was computed as: $TOM= \frac{\sum_{u\neq i,j} {\alpha_{iu}\alpha}_{uj}{+\alpha}_{ij}}{min(k_{i},k_{j})+1-\alpha_{ij}}$. Gene co-expression groups were defined as genes with high topologic overlap in expression and were determined as follows:

1) *Primary gene group determination*. Selected genes were clustered by hierarchical clustering of TOM-based dissimilarity matrix (i.e., 1-TOM matrix). The dynamic tree cut method was applied to cut the branches (i.e., genes) of the hierarchical tree, and genes within the same branch were combined into a group.

2) *Merging of highly similar groups*. Principal components of each gene co-expression group were computed using the corresponding *adjacency* matrix. The principal components were defined as *eigengenes* for each group and further used to determine group similarity. Groups were clustered by hierarchical clustering of 1-correlation matrix of *eigengenes*. Branches (i.e. gene co-expression groups) that had a height <0.25, corresponding to a correlation coefficient ≥0.75 were merged as final determined groups.

Correlation between the final groups and 11 phenotype variables was computed as Pearson correlation coefficients between *eigengenes* for each group and indicator variables for each phenotype. The significance of correlation was computed using one-tailed student’s t-tests.

## Ontological Enrichment Analysis

For primary RNA-Seq results, to test whether ontological categories were overrepresented among genes based on their differential expression ranking, gene set enrichment analysis (GSEA) was performed with the fast gene set enrichment analysis algorithm implemented in the fgsea R package (v.1.4.1) [11]. KEGG and Reactome pathway annotation gene sets were downloaded from MsigDB collections (v.6.2) (<http://software.broadinstitute.org/gsea/msigdb/collections.jsp>) and those with gene number >15 and <500 were included for analysis. Genes were sorted according to decreasing *t-statistics* from DESeq2 results. If multiple Ensembl gene IDs were annotated to the same gene symbol, the one with the largest absolute t-statistic was retained. Gene-level score for a gene in rank k was calculated by adding a positive or negative value, based on the presence or absence of the gene in the gene set, to the score of the gene in rank k-1, and positive values were weighted by gene k’s t-statistic. The enrichment score for a particular gene set was designated as the largest absolute value from all gene scores. P-values were assessed based on the distribution of enrichment scores following 10,000 permutations, and subsequently, q-values were obtained using the Benjamini-Hochberg approach. The leading-edge subsets, defined as genes in the ranked list that appear before and at the position of a positive gene-set enrichment score, or that appear at and after the position of a negative gene set enrichment score, corresponded to genes that drove the enrichment signal. Dependent pathways with q-value <0.05 were collapsed using the *collapsedPathways* function in fgsea.

For select WGCNA co-expression groups with 100-300 genes, to test whether genes from a particular pathway were more likely to be observed in a given co-expression group than those randomly observed in the genome, ontological category enrichment analysis was performed using an adopted Fisher’s exact test, also known as the “EASE score” in the DAVID NIH resource (<https://david.ncifcrf.gov/helps/functional_annotation.html>) [12,13]. As *background* genes we selected the 24,958 human genes tested in differential expression analysis whose gene symbols were available in the Ensembl database (2016 archive). A 2×2 contingency table was constructed to compare the numbers of genes in a pathway that were also in a given co-expression group minus one (cell 1), not in in a given co-expression group (cell 2) versus the number of genes in the human genome that were in the pathway plus one (cell 3) and not in the pathway (cell 4) the pathway. A one-tailed Fisher’s exact test was then used to test whether genes in a given co-expression group were more likely to be enriched in the pathway compared to genes randomly selected from the human genome. The Benjamini-Hochberg approach was used to correct for multiple comparisons made (i.e., number of ontological categories considered) and q-value <0.05 was considered significant.

## ChIP-Seq Data Analysis

ChIP-Seq data available in GEO entry GSE32465, which corresponds to a study that measured CEBPD-binding sites in HepG2 liver hepatocellular and K562 myelogenous leukemia cell lines at baseline [14], were obtained and analyzed with the brocade pipeline (<https://github.com/HimesGroup/brocade>) [15]. Briefly, raw sequence data in *fastq* format was downloaded from SRA and phenotype information was extracted from the *phenoData* object of the GSE Matrix file; reads were aligned to the hg38 reference genome using BWA [16]; Bamtools [5] was used to count and summarize the number of mapped reads; and CEBPD-binding sites were identified using MACS2 [17] where narrow peak calling was assigned. Aligned read files were converted to *bigwig* format and uploaded to the UCSC Genome Brower for peak visualization.

## Immunoblots

ASM cells derived from six non-smoking donors without chronic disease were transfected with NT or *CEBPD* siRNA using HiPerFect^®^ Transfection Reagent (Qiagen, Hilden, Germany), and then exposed to control (DMSO), IL-6 (10 ng/ml, 30 min), BUD (100 nM, 24 hr), or BUD+IL-6. Cells were then treated with a final concentration of 0.6 N perchloric acid, plates scraped, and cells pelleted. Pellets were solubilized in RIPA buffer and sonicated prior to being treated with SDS PAGE and transferred to nitrocellulose membranes, as previously described ([Balenga *et al.*, 2015](#_ENREF_4)). Primary antibodies used in the study included: rabbit polyclonal CEBPD (Rockland, Pottstown, PA), rabbit polyclonal IL-6Rα (MyBioSource, San Diego, CA), mouse monoclonal α Tubulin (Cell Signaling Technology, Danvers, MA), rabbit monoclonal phosphorylated STAT3 (pSTAT3) (Cell Signaling Technology), and mouse monoclonal STAT3 (Cell Signaling Technology). Immunoblot signals were obtained utilizing the Odyssey CLx LI-COR Imager (LI-COR Biosciences, Lincoln, NE) and band densities quantified using the LI-COR ImageStudio Software. Immunoblot band density quantification results for CEBPD and IL-6R were normalized to tubulin, and for pSTAT3 were normalized to STAT3. Quantification levels were normalized to NT siRNA control to confirm that CEBPD knockdown was successful. After this verification, quantification levels were normalized to the expression levels under NT siRNA control or *CEBPD* siRNA control within groups based on siRNA status. The statistical significance between groups was measured using paired two-tailed student’s t-tests. The ratios of signals were visualized as barplots of height equivalent to the mean across donors and error bars representing standard errors (SEs) across replicates.

## Primary ASM Cell Culture, Transfection, Exposure, and Traction Microscopy

ASM cells from five non-smoking donors without chronic disease were cultured in Ham’s F12 medium supplemented with 10% FBS, 50 units/mL penicillin, and 100 μg/mL streptomycin. Only early-passage ASM cells were used for all experiments [18,19]. *CEBPD* siRNA or NT siRNA scrambled control (Qiagen, Valencia, CA) were transfected at the concentration of 25nM using RNAiMax (Life technologies, Gaithersburg, MD) according to manufacturer’s instructions. To test the knockdown efficiency after transfection, ASM cells were harvested at 48 hours after transfection. Total RNA was extracted using the miRNAeasy mini kit (Qiagen, Valencia, CA) and reverse transcription was performed using Superscript III kit (Life technologies, Gaithersburg, MD). β actin was used as internal control in all experiments. Expression was detected in triplicates by qPCR using SYBR Mix.

Fourier transfer traction microscopy (FTTM) [20] was used to measure the contractile force of ASM cells. In brief, FTTM measures the deformation of beads embedded in a soft substrate and converts this to cells’ traction (contractile force per unit area) exerted by cells consistent with that deformation. We fabricated polyacrylamide (PA) gels of known stiffness at a physiological condition (8kPa). After coating the PA gels with 0.2μm fluorescent beads (505/515, Invitrogen, Carlsbad, CA) and bovine collagen, ASM cells were seeded on the PA gel in near confluency. The cells attached to the gel and deformed it by traction, causing the fluorescent beads to change position. Bead displacements were calculated by comparing the positions of beads before and after adding conditioned media or a contractile reagent to the ASM cells. With knowledge of the elastic properties of the PA gels, we then determined the traction exerted by ASM cells using custom made code in Matlab (Mathwork, Waltham, MA). Traction was reported as the root mean square traction. To develop a contractile phenotype in ASM cells, all cells were serum starved for 48 hours prior to traction measurement. In all experiments, a baseline traction (T_0_) was first recorded at the beginning of the experiment before any treatment was introduced to the cell. We then tested the response of ASM cells to a contractile agonist (i.e., histamine) and a β_2_ agonist (i.e., isoproterenol). After baseline measurement, ASM cells were incubated with histamine (1μM) for 5 minutes and the contractile force was measured again (T_His_) and normalized to the baseline traction (T_0_). Histamine was removed after the traction measurement and the ASM cells were provided with fresh media and put back in the incubator after washing twice with warm PBS. ASM cells were rested for two hours and then incubated with isoproterenol (1μM) for 5 minutes, after which the traction force was measured again (T_Iso_) and normalized to the baseline traction. For each donor, at least 12 replicate wells were used for traction measurements. The statistical significance of mean traction changes per donor across the five donors and between treatment conditions was assessed using two-tailed student’s t-tests. Traction measurements were visualized as barplots of height equivalent to the mean of means across the five donors with error bars representing standard errors (SEs) across these replicates.

# Detailed RNA-Seq Quality Control (QC) Results

ASM cells were obtained from four non-asthma donors (2 male and 2 female, age: 20.5±3.5 yrs. (mean±standard deviation)). All donors were white non-smokers with no evidence of chronic disease who died in motor vehicle accidents. ASM cells were transfected with NT or *CEBPD* siRNA, and exposed to control, BUD, TNFα and BUD+TNFα, resulting in 32 RNA samples. Based on qPCR results to ensure that CEBPD knockdown was successful, one sample was not selected for sequencing (Figure E1). Table E1 provides the characteristics of RNA-Seq QC for the 31 samples sequenced. We obtained an average of 52.1 million trimmed sequencing reads per sample (range 45.4-58.6 million reads per sample). An average of 82.5% of RNA-Seq reads aligned to hg38 genome reference files downloaded from Illumina’s iGenomes project (range 79.0%-86.6%), and an average of 33.0% of the mapped reads spanned junctions (range 28.9%-35.6%). An average of 85.0% of bases in mapped reads corresponded to mRNA (range 78.2%-89.1%). An average of 310.7 bp insert size of cDNA fragments (range 257.7-337.8 bp) was estimated from the sequencing data. Plots of normalized read coverage of transcripts vs. normalized position revealed that there was even coverage of transcripts (Figure E2A). Two housekeeping genes (*GABARAP* and *RPL19*) were used as positive controls for sequencing, and as expected, they were highly expressed, and their levels did not differ significantly by treatment or disease status (Figure E2B). Following the above QC results, all sequenced samples were deemed of sufficiently high quality to include in differential expression analyses.

# **Table E1. RNA-Seq Quality Control Metrics.**

Each sample sequenced yielded high quality RNA-Seq data based on the number of raw reads after adaptor trimming (number of reads in both R1 and R2), the percentage of mapped reads among the total number of raw reads, the percentage of junction spanning reads among the mapped reads, the percentage of mapped bases that mapped to mRNA, and the mean insert size of mapped reads. BUD: budesonide; NT: non-targeting.

| Donor | siRNA Transfection Status | Exposure | Total Raw Reads (Million) | Mapped Reads (Percent) | Junction Spanning Reads (Percent) | mRNA Base (Percent) | Mean Insert Size (bp) |
| --- | --- | --- | --- | --- | --- | --- | --- |
| N010912 | NT | control | 55.6 | 81.4 | 32.4 | 86 | 302.3 |
| N010912 | NT | BUD | 56.8 | 79 | 32.5 | 86.2 | 292.7 |
| N010912 | NT | TNFα | 57 | 86.6 | 33.1 | 86.5 | 307.9 |
| N010912 | NT | BUD+TNFα | 54.5 | 85.8 | 31.5 | 82.6 | 299.4 |
| N010912 | CEBPD | control | 50.5 | 84.2 | 33.8 | 86.4 | 323.2 |
| N010912 | CEBPD | TNFα | 49.8 | 83.4 | 33.6 | 87.4 | 318.9 |
| N010912 | CEBPD | BUD+TNFα | 58.6 | 79.9 | 28.9 | 78.2 | 257.7 |
| N021612 | NT | control | 52.6 | 81.3 | 34.2 | 87.4 | 322.2 |
| N021612 | NT | BUD | 46.6 | 83.1 | 35.3 | 87 | 330.3 |
| N021612 | NT | TNFα | 45.4 | 82.9 | 34.7 | 88.3 | 328.6 |
| N021612 | NT | BUD+TNFα | 48.1 | 80.6 | 31 | 80.5 | 285.9 |
| N021612 | CEBPD | control | 51.8 | 80.9 | 30.2 | 78.8 | 282.3 |
| N021612 | CEBPD | BUD | 50.8 | 84.6 | 31 | 78.5 | 296.7 |
| N021612 | CEBPD | TNFα | 48.6 | 84.1 | 34.4 | 86.8 | 329.3 |
| N021612 | CEBPD | BUD+TNFα | 49.1 | 85.3 | 30.4 | 78.6 | 300.4 |
| N051911 | NT | control | 54.1 | 82.6 | 32.2 | 84.4 | 305.6 |
| N051911 | NT | BUD | 54 | 82.4 | 33 | 85.3 | 309.2 |
| N051911 | NT | TNFα | 54.3 | 82.5 | 32.6 | 85.1 | 310.8 |
| N051911 | NT | BUD+TNFα | 58.3 | 81.1 | 35.4 | 88.8 | 335.1 |
| N051911 | CEBPD | control | 49.7 | 82.1 | 35.1 | 87.3 | 327 |
| N051911 | CEBPD | BUD | 47.7 | 82.5 | 35.6 | 87.5 | 337.8 |
| N051911 | CEBPD | TNFα | 49.5 | 81.5 | 31 | 80.3 | 301.8 |
| N051911 | CEBPD | BUD+TNFα | 51.5 | 82.8 | 34.7 | 87 | 312.9 |
| N061311 | NT | control | 49.5 | 80.5 | 34.4 | 88.6 | 336.4 |
| N061311 | NT | BUD | 56.1 | 80.7 | 34.8 | 89.1 | 321 |
| N061311 | NT | TNFα | 54.7 | 80.9 | 34.5 | 88.7 | 320.6 |
| N061311 | NT | BUD+TNFα | 55 | 80.3 | 30.8 | 80.8 | 282.9 |
| N061311 | CEBPD | control | 50.2 | 85.3 | 32.4 | 83.2 | 300.4 |
| N061311 | CEBPD | BUD | 48.2 | 84.5 | 31.6 | 81.1 | 299.8 |
| N061311 | CEBPD | TNFα | 51.4 | 82.2 | 34.7 | 88.8 | 323.6 |
| N061311 | CEBPD | BUD+TNFα | 54.9 | 82.8 | 34.6 | 88.3 | 327.5 |

# Table E2. Gene set enrichment analysis results corresponding to the *CEBPD* siRNA versus NT siRNA comparisons.

Significantly enriched pathways (q-value <0.05, normalized enrichment score (NES) >2) corresponding to the gene set enrichment analyses obtained based on the genes differential expressed for the *CEBPD* siRNA versus NT siRNA comparisons within each of the four exposure conditions (i.e., control, TNFα, BUD, and BUD+TNFα) are shown. Genes in Leading Edge are ordered based on their absolute t-statistics. BUD: budesonide; NT: non-targeting.

| **Pathway** | **Size** | **q-value** | **NES** | **Genes in Leading Edge** |
| --- | --- | --- | --- | --- |
| **CEBPD siRNA versus NT siRNA Among Cells with Control Exposure** | | | | |
| Reactome: peptide chain elongation | 104 | 0.006 | -3.09 | *RPS15,RPL18A,RPL3,EEF1A1,RPL12,RPS7,RPL13,RPL14,RPS8,EEF2,RPS3,RPL10,RPL13A,RPL28,RPL7,RPS4X,RPS9,RPL4,RPL10A,RPS23,RPL18,RPS27,RPL23,RPS20,RPL37,RPS12,RPS16,RPL15,RPL24,RPL30,RPS6,RPL19,RPS2,RPL32,RPS29,RPLP1,RPL5,RPS3A,RPL7A,RPS14,RPS28,RPS25,RPL22,RPL34,RPS11,RPL11,RPL27A,RPL37A,RPL23A,RPL31,RPS5,RPL39,RPL21,RPS27A,RPS21,RPL9,RPL23AP42,RPL29,RPS28P7,RPS15A,RPL26,RPL35,RPS17,RPS18,RPL8,RPS15AP11,RPLP2,RPL6,RPL35A,UBA52,RPL7AP66,RPS13,RPL23AP2,RPL27,RPLP0,RPL36,RPS15P5,RPS24* |
| KEGG: ribosome | 86 | 0.006 | -3.07 | *RPS15,RPL18A,RPL22L1,RPL3,RPL12,RPS7,RPL13,RPL14,RPS8,RPS3,RPL10,RPL13A,RPL28,RPL7,RPS4X,RPS9,RPL4,RPL10A,RPS23,RPL18,RPS27,RPL23,RPS20,RPL37,RPS12,RPS16,RPL15,RPL24,RPL30,RPS6,RPL19,RPS2,RPL32,RPS29,RPLP1,RPL5,RPS3A,RPL7A,RPS28,RPS25,RPL22,RPL34,RPS11,RPL11,RPL27A,RPL37A,RPL23A,RPL31,RPS5,RPL39,RPL21,RPS27A,RPS21,RPL9,RPL29,RPS15A,RPL26,RPL35,RPS17,RPS18,RPL8,RPLP2,RPL6,RPL35A,UBA52,RPS13,RPL27,RPLP0,RPL36,RPS24,RSL24D1,RPL36AL* |
| Reactome: 3' UTR mediated translational regulation | 125 | 0.006 | -3.00 | *RPS15,RPL18A,RPL3,PABPC1,RPL12,RPS7,EIF4B,RPL13,RPL14,RPS8,RPS3,RPL10,RPL13A,RPL28,RPL7,EIF3E,RPS4X,RPS9,RPL4,RPL10A,RPS23,RPL18,RPS27,RPL23,RPS20,RPL37,RPS12,RPS16,RPL15,RPL24,RPL30,RPS6,RPL19,RPS2,RPL32,RPS29,RPLP1,RPL5,EIF3F,RPS3A,RPL7A,RPS14,RPS28,RPS25,RPL22,RPL34,RPS11,RPL11,RPL27A,RPL37A,RPL23A,RPL31,RPS5,RPL39,EIF3H,RPL21,RPS27A,RPS21,RPL9,EIF4A2,RPL23AP42,RPL29,RPS28P7,RPS15A,RPL26,RPL35,RPS17,RPS18,RPL8,RPS15AP11,RPLP2,RPL6,RPL35A,UBA52,EIF3FP3,RPL7AP66,RPS13,EIF2S3,RPL23AP2,RPL27,RPLP0,RPL36,RPS15P5,RPS24* |
| Reactome: influenza viral RNA transcription and replication | 119 | 0.006 | -2.92 | *RPS15,RPL18A,RPL3,RPL12,RPS7,RPL13,DNAJC3,RPL14,RPS8,RPS3,RPL10,RPL13A,RPL28,RPL7,RPS4X,RPS9,RPL4,RPL10A,RPS23,RPL18,RPS27,RPL23,RPS20,RPL37,RPS12,RPS16,RPL15,RPL24,RPL30,RPS6,RPL19,RPS2,RPL32,RPS29,RPLP1,RPL5,RPS3A,RPL7A,RPS14,RPS28,RPS25,RPL22,RPL34,RPS11,RPL11,RPL27A,RPL37A,RPL23A,RPL31,RPS5,RPL39,RPL21,RPS27A,RPS21,RPL9,RPL23AP42,RPL29,RPS28P7,RPS15A,RPL26,RPL35,RPS17,RPS18,RPL8,RPS15AP11,RPLP2,RPL6,RPL35A,UBA52,RPL7AP66,RPS13,RPL23AP2,RPL27,RPLP0,RPL36,RPS15P5,RPS24* |
| Reactome: nonsense mediated decay enhanced by the exon junction complex | 125 | 0.006 | -2.86 | *RPS15,RPL18A,RPL3,PABPC1,RPL12,RPS7,RPL13,RPL14,RPS8,RPS3,RPL10,RPL13A,RPL28,RPL7,RPS4X,RPS9,RPL4,RPL10A,RPS23,RPL18,RPS27,RPL23,RPS20,RPL37,RPS12,RPS16,RPL15,RPL24,RPL30,RPS6,RPL19,RPS2,RPL32,RPS29,RPLP1,RPL5,RPS3A,RPL7A,RPS14,RPS28,RPS25,RPL22,RPL34,RPS11,RPL11,RPL27A,RPL37A,RPL23A,RPL31,RPS5,RPL39,RPL21,RPS27A,RPS21,RPL9,RPL23AP42,RPL29,RPS28P7,RPS15A,RPL26,RPL35,RPS17,RPS18,RPL8,RPS15AP11,RPLP2,RPL6,RPL35A,UBA52,RPL7AP66,RPS13,RPL23AP2,RPL27,RPLP0,RPL36,RPS15P5,RPS24* |
| Reactome: SRP-dependent cotranslational protein targeting to membrane | 127 | 0.006 | -2.84 | *RPS15,RPL18A,RPL3,SSR2,RPL12,RPS7,RPL13,RPL14,RPS8,RPS3,RPL10,RPL13A,RPL28,RPL7,RPS4X,RPS9,RPL4,RPL10A,RPS23,RPL18,RPS27,RPL23,RPS20,RPL37,RPS12,RPS16,RPL15,RPL24,RPL30,RPS6,RPL19,RPS2,RPL32,RPS29,RPLP1,RPL5,RPS3A,RPL7A,RPS14,RPS28,RPS25,RPL22,RPL34,RPS11,RPL11,RPL27A,RPL37A,RPL23A,RPL31,RPS5,RPL39,TRAM1,RPL21,RPS27A,RPS21,RPL9,RPL23AP42,RPL29,RPS28P7,RPS15A,RPL26,RPL35,SEC11A,RPS17,RPS18,RPL8,RPS15AP11,RPLP2,RPL6,RPL35A,UBA52,RPL7AP66,RPS13,RPL23AP2,RPL27,RPLP0,RPL36,RPS15P5,RPS24* |
| Reactome: influenza life cycle | 152 | 0.006 | -2.63 | *RPS15,RPL18A,RPL3,RPL12,RPS7,RPL13,DNAJC3,RPL14,RPS8,RPS3,RPL10,RPL13A,RPL28,RPL7,RPS4X,RPS9,RPL4,RPL10A,RPS23,RPL18,RPS27,RPL23,RPS20,RPL37,RPS12,RPS16,RPL15,RPL24,RPL30,RPS6,RPL19,RPS2,RPL32,RPS29,RPLP1,RPL5,RPS3A,RPL7A,RPS14,RPS28,RPS25,RPL22,RPL34,RPS11,RPL11,RPL27A,RPL37A,RPL23A,RPL31,RPS5,RPL39,RPL21,RPS27A,RPS21,RPL9,RPL23AP42,RPL29,RPS28P7,RPS15A,RPL26,RPL35,RPS17,RPS18,RPL8,RPS15AP11,RPLP2,RPL6,RPL35A,UBA52,RPL7AP66,RPS13* |
| Reactome: metabolism of mRNA | 227 | 0.006 | -2.20 | *RPS15,RPL18A,RPL3,PABPC1,RPL12,RPS7,EIF4B,RPL13,RPL14,RPS8,RPS3,RPL10,RPL13A,RPL28,RPL7,RPS4X,RPS9,RPL4,RPL10A,RPS23,RPL18,ZFP36,RPS27,RPL23,RPS20,RPL37,RPS12,RPS16,DCP1A,RPL15,RPL24,RPL30,RPS6,RPL19,RPS2,RPL32,RPS29,RPLP1,RPL5,RPS3A,RPL7A,RPS14,RPS28,RPS25,RPL22,RPL34,RPS11,RPL11,RPL27A,RPL37A,RPL23A,TNKS1BP1,RPL31,RPS5,RPL39,RPL21,PSME1,RPS27A,RPS21,RPL9,EIF4A2,RPL23AP42,RPL29,RPS28P7,RPS15A,RPL26,RPL35,RPS17,RPS18,RPL8,RPS15AP11,RPLP2,RPL6,RPL35A,UBA52,PSMB9,RPL7AP66,PSMF1,RPS13,HSPB1,HNRNPD,PATL1,RPL23AP2,PSMB8,RPL27,RPLP0,RPL36,RPS15P5,RPS24* |
| Reactome: interferon alpha beta signaling | 48 | 0.006 | -2.08 | *GBP2,SOCS3,STAT1,IFITM3,STAT2,HLA-B,IRF2,IFITM2,HLA-A,IFITM1,HLA-C,SOCS1,IFNAR1,HLA-F,IFI27,IFIT3,IFIT2,IFI35,IFNAR2,PSMB8,IFI6,IFIT1* |
| Reactome: metabolism of RNA | 272 | 0.006 | -2.01 | *RPS15,RPL18A,RPL3,PABPC1,RPL12,RPS7,EIF4B,RPL13,RPL14,RPS8,RPS3,RPL10,RPL13A,RPL28,RPL7,RPS4X,RPS9,RPL4,RPL10A,RPS23,RPL18,ZFP36,RPS27,RPL23,RPS20,RPL37,RPS12,RPS16,DCP1A,RPL15,RPL24,RPL30,RPS6,RPL19,RPS2,RPL32,RPS29,RPLP1,RPL5,RPS3A,RPL7A,RPS14,RPS28,RPS25,RPL22,RPL34,RPS11,RPL11,RPL27A,RPL37A,RPL23A,TNKS1BP1,RPL31,RPS5,RPL39,RPL21,PSME1,RPS27A,RPS21,RPL9,EIF4A2,RPL23AP42,RPL29,RPS28P7,RPS15A,RPL26,RPL35,RPS17,RPS18,RPL8,RPS15AP11,RPLP2,RPL6,RPL35A,UBA52,PSMB9,RPL7AP66,PSMF1,RPS13,HSPB1,SNRPD2,HNRNPD,PATL1,RPL23AP2,PSMB8,RPL27,RPLP0,RPL36,RPS15P5* |
| KEGG: p53 signaling pathway | 61 | 0.006 | 2.07 | *CDKN1A,TNFRSF10B,RRM2B,ZMAT3,MDM2,CDK6,SESN1,ATR,DDB2,APAF1,FAS,PPM1D,CCND3,THBS1,TP53,CYCS,EI24,STEAP3,BAX* |
| Reactome: formation of tubulin folding intermediates by CCT/TriC | 18 | 0.006 | 2.10 | *CCT5,CCT6A,CCT2,TUBB4B,TCP1,CCT7,CCT4,TUBA4A,CCT8,CCT3,TUBB6,TUBB2A* |
| **CEBPD siRNA versus NT siRNA Among Cells with TNFα Exposure** | | | | |
| KEGG: ribosome | 86 | 0.006 | -2.91 | *RPS15,RPL18A,RPL22L1,RPL3,RPS7,RPL4,RPS8,RPS23,RPL14,RPS4X,RPS3,RPL7,RPL13A,RPL13,RPL15,RPL12,RPL10A,RPS12,RPL28,RPS9,RPL23,RPS27,RPL37,RPL24,RPL34,RPL30,RPS25,RPL35,RPL19,RPL32,RPL5,RPS20,RPL31,RPL21,RPL7A,RPS27A,RPS6,RPS15A,RPL23A,RPS3A,RPL10,RPL18,RPS11,RPS29,RPL11,RPLP1,RPS13,RPL27A,RPL22,RPS16,RPL37A,RPS2,RPL39,RPL9,RPS4Y1,RPS5,RPS18,RPL6,RPL29* |
| Reactome: peptide chain elongation | 103 | 0.006 | -2.84 | *RPS15,RPL18A,RPL3,EEF1A1,RPS7,RPL4,RPS8,RPS23,RPL14,RPS4X,RPS3,RPL7,RPL13A,RPL13,RPL15,RPL12,RPL10A,RPS12,EEF2,RPL28,RPS9,RPL23,RPS27,RPL37,RPL24,RPL34,RPL30,RPS25,RPL35,RPL19,RPL32,RPL5,RPS20,RPL31,RPL21,RPL7A,RPS27A,RPS6,RPS15A,RPL23A,RPS3A,RPL10,RPL18,RPS11,RPS29,RPL11,RPLP1,RPS13,RPL27A,RPL22,RPS16,RPL37A,RPS2,RPL39,RPL9,RPS14,RPS4Y1,RPS5,RPS18,RPL6,RPL29* |
| Reactome: 3' UTR mediated translational regulation | 124 | 0.006 | -2.75 | *RPS15,RPL18A,PABPC1,RPL3,RPS7,RPL4,RPS8,RPS23,RPL14,RPS4X,RPS3,EIF4B,RPL7,RPL13A,RPL13,RPL15,RPL12,RPL10A,RPS12,RPL28,RPS9,RPL23,RPS27,RPL37,RPL24,RPL34,RPL30,RPS25,RPL35,RPL19,RPL32,RPL5,RPS20,EIF4A2,RPL31,RPL21,RPL7A,RPS27A,RPS6,RPS15A,RPL23A,RPS3A,EIF3E,RPL10,RPL18,RPS11,RPS29,EIF3F,RPL11,RPLP1,RPS13,EIF3H,RPL27A,RPL22,RPS16,RPL37A,RPS2,RPL39,RPL9,RPS14,RPS4Y1,RPS5,RPS18,RPL6,RPL29,RPS17,RPS28,EIF3B,RPS24,RPL8,EIF2S3,RPL35A,RPLP0,RPL22P11,RPL34P27* |
| Reactome: nonsense mediated decay enhanced by the exon junction complex | 124 | 0.006 | -2.66 | *RPS15,RPL18A,PABPC1,RPL3,RPS7,RPL4,RPS8,RPS23,RPL14,RPS4X,RPS3,RPL7,RPL13A,RPL13,RPL15,RPL12,RPL10A,RPS12,RPL28,RPS9,RPL23,RPS27,RPL37,RPL24,RPL34,RPL30,RPS25,RPL35,RPL19,RPL32,RPL5,RPS20,RPL31,RPL21,RPL7A,RPS27A,RPS6,RPS15A,RPL23A,RPS3A,RPL10,RPL18,RPS11,RPS29,RPL11,RPLP1,RPS13,RPL27A,RPL22,RPS16,RPL37A,RPS2,RPL39,RPL9,RPS14,RPS4Y1,RPS5,RPS18,RPL6,RPL29* |
| Reactome: influenza viral RNA transcription and replication | 118 | 0.006 | -2.66 | *RPS15,RPL18A,RPL3,RPS7,DNAJC3,RPL4,RPS8,RPS23,RPL14,RPS4X,RPS3,RPL7,RPL13A,RPL13,RPL15,RPL12,RPL10A,RPS12,RPL28,RPS9,RPL23,RPS27,RPL37,RPL24,RPL34,RPL30,RPS25,RPL35,RPL19,RPL32,RPL5,RPS20,RPL31,RPL21,RPL7A,RPS27A,RPS6,RPS15A,RPL23A,RPS3A,RPL10,RPL18,RPS11,RPS29,RPL11,RPLP1,RPS13,RPL27A,RPL22,RPS16,RPL37A,RPS2,RPL39,RPL9,IPO5,RPS14,RPS4Y1,RPS5,RPS18,RPL6,RPL29* |
| Reactome: SRP-dependent cotranslational protein targeting to membrane | 126 | 0.006 | -2.54 | *RPS15,RPL18A,RPL3,SSR2,RPS7,RPL4,RPS8,RPS23,RPL14,RPS4X,RPS3,RPL7,RPL13A,RPL13,RPL15,RPL12,RPL10A,RPS12,RPL28,RPS9,RPL23,RPS27,RPL37,RPL24,RPL34,RPL30,RPS25,RPL35,RPL19,RPL32,RPL5,RPS20,RPL31,RPL21,RPL7A,RPS27A,RPS6,RPS15A,RPL23A,RPS3A,RPL10,RPL18,RPS11,RPS29,RPL11,RPLP1,RPS13,RPL27A,RPL22,RPS16,RPL37A,RPS2,RPL39,RPL9,RPS14,RPS4Y1,RPS5,RPS18,RPL6,RPL29* |
| Reactome: influenza life cycle | 151 | 0.006 | -2.47 | *RPS15,RPL18A,RPL3,RPS7,DNAJC3,RPL4,RPS8,RPS23,RPL14,RPS4X,RPS3,RPL7,RPL13A,RPL13,RPL15,RPL12,RPL10A,RPS12,RPL28,RPS9,RPL23,RPS27,RPL37,RPL24,RPL34,RPL30,RPS25,RPL35,RPL19,RPL32,RPL5,RPS20,RPL31,RPL21,RPL7A,RPS27A,RPS6,RPS15A,RPL23A,RPS3A,RPL10,RPL18,RPS11,RPS29,RPL11,RPLP1,RPS13,RPL27A,RPL22,RPS16,RPL37A,RPS2,RPL39,RPL9,IPO5,RPS14,RPS4Y1,RPS5,RPS18,NUP93,RPL6,RPL29* |
| Reactome: smooth muscle contraction | 24 | 0.006 | -2.23 | *ITGA1,LMOD1,ACTG2,MYL9,MYLK,VCL,TPM4,TPM2,TLN1,MYL6,CALD1,MYH11,CALM2,CALM1* |
| Reactome: nitric oxide stimulates guanylate cyclase | 17 | 0.006 | -2.00 | *MRVI1,PRKG1,GUCY1B3,PDE5A,PDE3B,PDE3A* |
| Reactome: destabilization of mRNA by KSRP | 17 | 0.012 | 2.01 | *MAPK11,EXOSC6,DIS3,AKT1,DCP2,KHSRP,EXOSC9,EXOSC4,EXOSC1* |
| Reactome: RNA pol II transcription pre-initiation and promoter opening | 38 | 0.006 | 2.04 | *TAF13,ERCC3,GTF2H3,POLR2A,POLR2H,TAF11,GTF2B,MNAT1,GTF2E1,POLR2G,TAF1,GTF2F1,POLR2K,GTF2F2,POLR2C,POLR2B,CCNH,TAF5,TAF9,POLR2L* |
| Reactome: downstream signaling events of B cell receptor BCR | 90 | 0.006 | 2.12 | *CDKN1A,MDM2,NR4A1,AKT1S1,PSMB2,PSMB5,PSMB7,PSMC4,NRAS,AKT1,PSMD1,RASGRP3,FOXO1,HRAS,PSMA4,GSK3A,PSMD7,PSMC5,FBXW11,CHUK,THEM4,PSMC2,PSMB3,PSMC6,PSMB1,PSMA7,PSMD14,TRIB3,PSMB4,PSMD6,PSMD12,PDPK1,PSMA5,MTOR,PSMD3,PSMD11,PSMD2,BTRC,PSMB6,RICTOR,PSMD13,MLST8,NFKBIB,PSMD10* |
| Reactome: transferrin endocytosis and recycling | 23 | 0.006 | 2.22 | *ATP6V1G1,ATP6V1C1,MCOLN1,TCIRG1,ATP6V0B,ATP6V1E1,ATP6V1D,ATP6V0D1,STEAP3,TFRC,ATP6V1H,ATP6V0A2* |
| **CEBPD siRNA versus NT siRNA Among Cells with BUD Exposure** | | | | |
| Reactome: peptide chain elongation | 103 | 0.003 | -3.13 | *RPS15, RPL18A, RPL3, EEF1A1, RPL13A, RPL12, RPL28, RPL13, RPL10A, RPS3, RPL10, RPS4X, EEF2, RPS23, RPL23, RPS9, RPS27, RPS8, RPL4, RPL7, RPS7, RPL14, RPS20, RPS25, RPL18, RPL37, RPL7A, RPL15, RPS12, RPS3A, RPL34, RPL32, RPL31, RPL19, RPS11, RPL37A, RPS16, RPL5, RPS14, RPL30, RPS15A, RPL24, RPL27A, RPS28, RPS6, RPL21, RPL39, RPL23AP42, RPS29, RPS5, RPL23A, RPL11, RPS2, RPL9, RPL22, RPL29, RPS4Y1, RPLP1, RPS21, RPS27A, RPS18, RPS17, RPL26, RPS13, RPS24, RPL35, RPS28P7, RPL35A, RPL6, RPL5P1, RPL7AP66, RPLP0, UBA52, RPL8, RPLP2, RPL38, RPS15AP11, RPL27, RPL34P27, RPL23AP2, RPL23AP18, RPSAP12* |
| KEGG: ribosome | 86 | 0.003 | -3.12 | *RPS15, RPL18A, RPL3, RPL13A, RPL12, RPL28, RPL13, RPL10A, RPS3, RPL10, RPL22L1, RPS4X, RPS23, RPL23, RPS9, RPS27, RPS8, RPL4, RPL7, RPS7, RPL14, RPS20, RPS25, RPL18, RPL37, RPL7A, RPL15, RPS12, RPS3A, RPL34, RPL32, RPL31, RPL19, RPS11, RPL37A, RPS16, RPL5, RPL30, RPS15A, RPL24, RPL27A, RPS28, RPS6, RPL21, RPL39, RPS29, RPS5, RPL23A, RPL11, RPS2, RPL9, RPL22, RPL29, RPS4Y1, RPLP1, RPS21, RPS27A, RPS18, RPS17, RPL26, RPS13, RPS24, RPL35, RPL35A, RPL6, RPLP0, UBA52, RPL8, RPLP2, RSL24D1* |
| Reactome: influenza viral RNA transcription and replication | 118 | 0.003 | -3.01 | *RPS15, RPL18A, RPL3, RPL13A, RPL12, DNAJC3, RPL28, RPL13, RPL10A, RPS3, RPL10, RPS4X, RPS23, RPL23, RPS9, RPS27, RPS8, RPL4, RPL7, RPS7, RPL14, RPS20, RPS25, RPL18, RPL37, RPL7A, RPL15, RPS12, RPS3A, RPL34, RPL32, RPL31, RPL19, RPS11, RPL37A, RPS16, RPL5, RPS14, RPL30, RPS15A, RPL24, RPL27A, RPS28, RPS6, RPL21, RPL39, RPL23AP42, RPS29, RPS5, RPL23A, RPL11, RPS2, RPL9, RPL22, RPL29, RPS4Y1, RPLP1, RPS21, RPS27A, RPS18, RPS17, RPL26, RPS13, RPS24, RPL35, RPS28P7, RPL35A, RPL6, RPL5P1, RPL7AP66, RPLP0, UBA52, RPL8, RPLP2, RPL38, RPS15AP11, RPL27, RPL34P27, RPL23AP2, RPL23AP18, RPSAP12* |
| Reactome: 3' UTR mediated translational regulation | 124 | 0.003 | -2.98 | *RPS15, RPL18A, RPL3, EIF4B, RPL13A, RPL12, RPL28, PABPC1, RPL13, RPL10A, RPS3, RPL10, RPS4X, EIF3E, RPS23, RPL23, RPS9, RPS27, RPS8, RPL4, RPL7, RPS7, RPL14, RPS20, RPS25, RPL18, RPL37, RPL7A, RPL15, RPS12, RPS3A, RPL34, RPL32, RPL31, RPL19, RPS11, RPL37A, RPS16, RPL5, RPS14, RPL30, RPS15A, RPL24, RPL27A, RPS28, RPS6, EIF3H, RPL21, RPL39, RPL23AP42, RPS29, RPS5, RPL23A, RPL11, RPS2, RPL9, RPL22, EIF3F, RPL29, RPS4Y1, RPLP1, RPS21, RPS27A, RPS18, RPS17, RPL26, RPS13, RPS24, RPL35, RPS28P7, RPL35A, RPL6, EIF4A2, RPL5P1, RPL7AP66, RPLP0, UBA52, RPL8, RPLP2, RPL38, RPS15AP11, RPL27, RPL34P27, RPL23AP2, RPL23AP18, RPSAP12* |
| Reactome: nonsense mediated decay enhanced by the exon junction complex | 124 | 0.003 | -2.93 | *RPS15, RPL18A, RPL3, RPL13A, RPL12, RPL28, PABPC1, RPL13, RPL10A, RPS3, RPL10, RPS4X, RPS23, RPL23, RPS9, RPS27, RPS8, RPL4, RPL7, RPS7, RPL14, RPS20, RPS25, RPL18, RPL37, RPL7A, RPL15, RPS12, RPS3A, RPL34, RPL32, RPL31, RPL19, RPS11, RPL37A, RPS16, RPL5, RPS14, RPL30, RPS15A, RPL24, RPL27A, RPS28, RPS6, RPL21, RPL39, RPL23AP42, RPS29, RPS5, RPL23A, RPL11, RPS2, RPL9, RPL22, RPL29, RPS4Y1, RPLP1, RPS21, RPS27A, RPS18, RPS17, RPL26, RPS13, RPS24, RPL35, RPS28P7, RPL35A, RPL6, RPL5P1, RPL7AP66, RPLP0, UBA52, RPL8, SMG5, RPLP2, RPL38, RPS15AP11, RPL27, RPL34P27, RPL23AP2, RPL23AP18, RPSAP12* |
| Reactome: SRP-dependent cotranslational protein targeting to membrane | 126 | 0.003 | -2.92 | *RPS15, RPL18A, RPL3, RPL13A, RPL12, RPL28, RPL13, RPL10A, RPS3, RPL10, RPS4X, RPS23, RPL23, RPS9, RPS27, RPS8, RPL4, RPL7, RPS7, RPL14, RPS20, RPS25, RPL18, SSR2, RPL37, RPL7A, RPL15, RPS12, RPS3A, RPL34, RPL32, RPL31, RPL19, RPS11, RPL37A, RPS16, RPL5, RPS14, RPL30, RPS15A, RPL24, RPL27A, RPS28, RPS6, RPL21, RPL39, RPL23AP42, RPS29, RPS5, RPL23A, RPL11, RPS2, RPL9, RPL22, RPL29, RPS4Y1, RPLP1, RPS21, RPS27A, SEC11A, RPS18, RPS17, RPL26, RPS13, RPS24, RPL35, RPS28P7, RPL35A, RPL6, RPL5P1, RPL7AP66, RPLP0, UBA52, RPL8, RPLP2, RPL38, RPS15AP11, RPL27, RPL34P27, RPL23AP2, RPL23AP18, RPSAP12* |
| Reactome: influenza life cycle | 150 | 0.003 | -2.74 | *RPS15, RPL18A, RPL3, RPL13A, RPL12, DNAJC3, RPL28, RPL13, RPL10A, RPS3, RPL10, RPS4X, RPS23, RPL23, RPS9, RPS27, RPS8, RPL4, RPL7, RPS7, RPL14, RPS20, RPS25, RPL18, RPL37, RPL7A, RPL15, RPS12, RPS3A, RPL34, RPL32, RPL31, RPL19, RPS11, RPL37A, RPS16, RPL5, RPS14, RPL30, RPS15A, RPL24, RPL27A, RPS28, RPS6, RPL21, RPL39, RPL23AP42, RPS29, RPS5, RPL23A, RPL11, RPS2, RPL9, RPL22, RPL29, RPS4Y1, RPLP1, RPS21, RPS27A, RPS18, RPS17, RPL26, RPS13, RPS24, RPL35, RPS28P7, RPL35A, RPL6, RPL5P1, RPL7AP66, RPLP0, UBA52, RPL8, RPLP2, RPL38, RPS15AP11, RPL27, RPL34P27, RPL23AP2, RPL23AP18, RPSAP12* |
| Reactome: metabolism of mRNA | 226 | 0.003 | -2.28 | *RPS15, RPL18A, RPL3, EIF4B, RPL13A, RPL12, RPL28, PABPC1, RPL13, RPL10A, RPS3, RPL10, RPS4X, RPS23, RPL23, RPS9, RPS27, RPS8, RPL4, RPL7, RPS7, RPL14, RPS20, RPS25, RPL18, RPL37, RPL7A, RPL15, RPS12, RPS3A, RPL34, RPL32, RPL31, RPL19, RPS11, RPL37A, RPS16, RPL5, RPS14, RPL30, TNKS1BP1, RPS15A, RPL24, RPL27A, RPS28, RPS6, RPL21, RPL39, RPL23AP42, RPS29, RPS5, RPL23A, RPL11, RPS2, DCP1A, RPL9, RPL22, RPL29, RPS4Y1, RPLP1, RPS21, RPS27A, RPS18, RPS17, RPL26, RPS13, RPS24, RPL35, RPS28P7, RPL35A, ZFP36, RPL6, EIF4A2, RPL5P1, RPL7AP66, RPLP0, UBA52, RPL8, MAPKAPK2, SMG5, RPLP2, CNOT8, RPL38, RPS15AP11, PSME1, RPL27, RPL34P27, RPL23AP2, DCP1B, RPL23AP18, RPSAP12* |
| Reactome: biological oxidations | 78 | 0.003 | -2.13 | *ADH1B, FMO2, NNMT, ALDH2, FMO3, MAOA, MAT2B, CYP2U1, MGST1, MGST3, GSTM5, CYP27A1, CYP39A1, CYP24A1, UGP2, MAOB, PTGIS, SULT1A1, ACSS2, ALDH1A1, TPMT, CYP19A1, GCLM, CYP1B1, GGT5, ACSS1, ADH4, SLC35D1, ADH6, SULT4A1, BPNT1, GSTM4, CYP26C1, ADH7* |
| KEGG: drug metabolism cytochrome P450 | 34 | 0.003 | -2.10 | *ADH1B, AOX1, FMO2, FMO3, ADH5, MAOA, MGST1, MGST3, GSTM2, GSTM5, GSTK1, GSTM3, MAOB* |
| Reactome: metabolism of RNA | 270 | 0.003 | -2.08 | *RPS15, RPL18A, RPL3, EIF4B, RPL13A, RPL12, RPL28, PABPC1, RPL13, RPL10A, RPS3, RPL10, RPS4X, RPS23, RPL23, RPS9, RPS27, RPS8, RPL4, RPL7, RPS7, RPL14, RPS20, RPS25, RPL18, RPL37, RPL7A, RPL15, RPS12, RPS3A, RPL34, RPL32, RPL31, RPL19, RPS11, RPL37A, RPS16, RPL5, RPS14, RPL30, TNKS1BP1, RPS15A, RPL24, RPL27A, RPS28, RPS6, RPL21, RPL39, RPL23AP42, RPS29, RPS5, RPL23A, RPL11, RPS2, DCP1A, RPL9, RPL22, RPL29, RPS4Y1, RPLP1, RPS21, RPS27A, RPS18, RPS17, RPL26, RPS13, RPS24, RPL35, RPS28P7, RPL35A, ZFP36, RPL6, EIF4A2, RPL5P1, RPL7AP66, RPLP0, UBA52, RPL8, MAPKAPK2, SMG5, RPLP2, CNOT8, SNRPD2, RPL38, RPS15AP11, PSME1, RPL27, RPL34P27, RPL23AP2, DCP1B, RPL23AP18, RPSAP12* |
| Reactome: complement cascade | 17 | 0.003 | -2.08 | *C1S, CFH, MASP1, C3, CFD, CFB, CD46, C7, PROS1, CFI, C6, CD55* |
| Reactome: cell cycle mitotic | 305 | 0.003 | 2.04 | *CDKN1A, CDK6, CCND3, PSMB2, DYNC1H1, PCNA, TYMS, PPP2R1B, TUBGCP5, MAPRE1, PSMD1, CDC25B, RANBP2, PSMB7, PSMD11, CDC25A, PPP2CB, CENPO, SDCCAG8, PSMC5, PPP2CA, PPP2R2A, TUBB4B, PSMA4, PSMD14, MNAT1, PSMC2, PSMD7, HSP90AA1, PSMD6, PCNT, TUBG1, CEP290, PSMB5, ANAPC7, SEC13, YWHAG, POLA2, TUBGCP3, CENPT, RRM2, ZWILCH, KIF2A, RANGAP1, PSMC4, POLE2, PSMD12, ANAPC1, LIG1, POLE, CDCA8, PPP2R3B, TUBGCP2, PSMB3, SEH1L, CENPN, PSMD13, CDC27, RPA2, NUP85, PSME2, CCNE2, POLA1, KIF23, CCNB1, PSME4, BUB3, CDC20, PSMD2, CKS1B, CDK1, BTRC, XPO1, GINS4, ZWINT, CKAP5, PSMA3, PSMB1, KNTC1, CDT1, PAFAH1B1, PSMD10, PRKACA, B9D2, PSMC6, ANAPC5, SMC1A, CENPJ, PSMB6, YWHAE, RFC3, CEP41, ACTR1A, MCM4, ANAPC11, PSMD3, CENPL, TUBA1A, CASC5, AHCTF1, NEDD1, GORASP1, RPA4, ORC4, DYNC1I2, TK2, LIN52, TUBG2, CCNE1, CENPM, DSN1, PSMC1, PRIM2, DYNLL1, RFC2, NUDC, NUP43, FGFR1OP, RPA1, ANAPC2, LIN9, CDKN2D, UBE2C, AURKA, ZW10, POLD1, TUBA4A, ERCC6L, MCM7, GINS2, MCM8, SKA1, RCC2, CSNK1E, PSMD9, TFDP1, RFC5, FEN1, RBL1, ORC6, CENPH, SPC25, TUBGCP6, GINS1, TAOK1* |
| Reactome: formation of tubulin folding intermediates by CCT/TriC | 18 | 0.003 | 2.09 | *CCT6A, CCT5, TUBB4B, CCT3, TCP1, CCT7, CCT8, TUBA1B, CCT4, TUBA1C, CCT2, TUBA1A, TUBB3, TUBB2A, TUBB6* |
| Reactome: downstream signaling events of B cell receptor BCR | 89 | 0.003 | 2.18 | *CDKN1A, MDM2, NRAS, PSMB2, MTOR, PSMD1, PSMB7, PDPK1, PSMD11, AKT1, PSMC5, PSMA4, CHUK, PSMD14, NR4A1, PSMC2, PSMD7, HRAS, PSMD6, PSMB5, NFKBIE, AKT1S1, PSMC4, PSMD12, FBXW11, TRIB3, PSMB3, PSMD13, PSME2, PSME4, PSMD2, THEM4, GSK3A, BTRC, PSMA3, PSMB1, PSMD10, PSMC6, PSMB6, PSMD3, BCL10* |
| KEGG: p53 signaling pathway | 61 | 0.003 | 2.27 | *CDKN1A, CDK6, MDM2, ZMAT3, RRM2B, TNFRSF10B, SESN1, SERPINE1, CCND3, ATR, PPM1D, DDB2, SESN2, FAS, CYCS, SHISA5, TP53, APAF1, STEAP3, BBC3, EI24, PMAIP1, RRM2, BID* |
| Reactome: G1/S transition | 107 | 0.003 | 2.27 | *CDKN1A, PSMB2, PCNA, TYMS, PPP2R1B, PSMD1, PSMB7, PSMD11, CDC25A, PPP2CB, PSMC5, PPP2CA, PSMA4, PSMD14, MNAT1, PSMC2, PSMD7, PSMD6, PSMB5, POLA2, RRM2, PSMC4, POLE2, PSMD12, POLE, PPP2R3B, PSMB3, PSMD13, RPA2, PSME2, CCNE2, POLA1, CCNB1, PSMD2, CKS1B, CDK1, PSMA3, PSMB1, CDT1, PSMD10, PSMC6, PSMB6, MCM4, PSMD3, RPA4, ORC4, TK2, CCNE1, PSMC1, PRIM2* |
| **CEBPD siRNA versus NT siRNA Among Cells with BUD+TNFα Exposure** | | | | |
| KEGG: ribosome | 87 | 0.004 | -3.12 | *RPS15,RPL18A,RPL22L1,RPL28,RPL3,RPS7,RPL12,RPL14,RPL13,RPS3,RPL18,RPS8,RPL19,RPS2,RPL4,RPS9,RPL13A,RPL35,RPS4X,RPL30,RPS27,RPS6,RPL15,RPL27A,RPL7,RPS5,RPL7A,RPL32,RPS20,RPL10A,RPL37,RPL24,RPS12,RPS16,RPL23,RPS23,RPLP1,RPL34,RPL5,RPS25,RPL23A,RPL37A,RPS3A,RPL8,RPL29,RPL11,RPL10,RPS11,RPS28,RPS29,RPL21,RPL22,RPL9,UBA52,RPS4Y1,RPL31,RPS27A,RPS17,RPL6,RPS24,RPS15A,RPS21,RPS13,RPL27,RPSA,RPLP2,RPL35A,RPS18,RPL36,RPL39* |
| Reactome: peptide chain elongation | 104 | 0.004 | -3.06 | *RPS15,RPL18A,RPL28,RPL3,EEF2,RPS7,RPL12,RPL14,RPL13,RPS3,RPL18,RPS8,RPL19,RPS2,RPL4,RPS9,EEF1A1,RPL13A,RPL35,RPS4X,RPL30,RPS27,RPS6,RPL15,RPL27A,RPL7,RPS5,RPL7A,RPL32,RPS20,RPL10A,RPL37,RPL24,RPS12,RPS16,RPL23,RPS23,RPLP1,RPL34,RPL5,RPS25,RPL23A,RPL37A,RPS3A,RPL8,RPL29,RPL11,RPL10,RPS11,RPS28,RPS29,RPL21,RPL22,RPS14,RPL9,UBA52,RPS4Y1,RPL23AP18,RPL31,RPS27A,RPS17,RPL6,RPS24,RPS15A,RPS21,RPS13,RPL27,RPSA,RPLP2,RPL35A,RPS18,RPL36,RPL39,RPL23AP42,RPS15AP11* |
| Reactome: 3' UTR mediated translational regulation | 125 | 0.004 | -3.01 | *RPS15,RPL18A,PABPC1,RPL28,RPL3,RPS7,RPL12,RPL14,RPL13,RPS3,EIF4B,RPL18,RPS8,RPL19,RPS2,RPL4,RPS9,RPL13A,RPL35,RPS4X,RPL30,RPS27,RPS6,RPL15,RPL27A,EIF3H,RPL7,RPS5,RPL7A,RPL32,RPS20,RPL10A,RPL37,RPL24,RPS12,RPS16,RPL23,RPS23,RPLP1,RPL34,EIF3F,RPL5,RPS25,RPL23A,RPL37A,RPS3A,RPL8,RPL29,RPL11,RPL10,RPS11,RPS28,RPS29,RPL21,RPL22,RPS14,EIF3E,RPL9,UBA52,RPS4Y1,RPL23AP18,RPL31,RPS27A,RPS17,RPL6,RPS24,RPS15A,RPS21,RPS13,EIF3B,RPL27,RPSA,RPLP2,RPL35A,EIF3I,EIF4A2,RPS18,RPL36,RPL39,RPL23AP42,RPS15AP11* |
| Reactome: influenza viral RNA transcription and replication | 119 | 0.004 | -2.99 | *RPS15,RPL18A,RPL28,RPL3,RPS7,RPL12,RPL14,RPL13,RPS3,RPL18,RPS8,RPL19,RPS2,RPL4,RPS9,RPL13A,RPL35,RPS4X,RPL30,RPS27,DNAJC3,RPS6,RPL15,RPL27A,RPL7,RPS5,RPL7A,RPL32,RPS20,RPL10A,RPL37,RPL24,RPS12,RPS16,RPL23,RPS23,RPLP1,RPL34,RPL5,RPS25,RPL23A,RPL37A,RPS3A,RPL8,RPL29,RPL11,RPL10,RPS11,RPS28,RPS29,RPL21,RPL22,RPS14,RPL9,UBA52,RPS4Y1,RPL23AP18,RPL31,RPS27A,RPS17,RPL6,RPS24,RPS15A,RPS21,RPS13,RPL27,RPSA,RPLP2,RPL35A,RPS18,RPL36,RPL39,RPL23AP42,RPS15AP11,RPLP0,POLR2E,FAU,POLR2D,RPL5P1,RPL7AP66,RPL26,RPS26,POLR2L,RPL21P134,RPL38,RPS19,RPS15P5,RPS19P3,RPL41,RPL23AP74* |
| Reactome: nonsense mediated decay enhanced by the exon junction complex | 125 | 0.004 | -2.94 | *RPS15,RPL18A,PABPC1,RPL28,RPL3,RPS7,RPL12,RPL14,RPL13,RPS3,RPL18,RPS8,RPL19,RPS2,RPL4,RPS9,RPL13A,RPL35,RPS4X,RPL30,RPS27,RPS6,RPL15,RPL27A,RPL7,RPS5,RPL7A,RPL32,RPS20,RPL10A,RPL37,RPL24,RPS12,RPS16,RPL23,RPS23,RPLP1,RPL34,RPL5,RPS25,RPL23A,RPL37A,RPS3A,RPL8,RPL29,RPL11,RPL10,RPS11,RPS28,RPS29,RPL21,RPL22,RPS14,RPL9,UBA52,RPS4Y1,RPL23AP18,RPL31,RPS27A,RPS17,RPL6,RPS24,RPS15A,RPS21,RPS13,RPL27,RPSA,RPLP2,RPL35A,RPS18,RPL36,RPL39,RPL23AP42,RPS15AP11* |
| Reactome: influenza life cycle | 152 | 0.004 | -2.89 | *RPS15,RPL18A,RPL28,RPL3,RPS7,RPL12,RPL14,RPL13,RPS3,RPL18,RPS8,RPL19,RPS2,RPL4,RPS9,RPL13A,RPL35,RPS4X,RPL30,RPS27,DNAJC3,RPS6,RPL15,RPL27A,RPL7,RPS5,RPL7A,RPL32,RPS20,RPL10A,RPL37,RPL24,RPS12,RPS16,RPL23,RPS23,RPLP1,RPL34,RPL5,RPS25,RPL23A,RPL37A,RPS3A,RPL8,RPL29,RPL11,RPL10,RPS11,RPS28,RPS29,RPL21,RPL22,RPS14,RPL9,UBA52,RPS4Y1,RPL23AP18,RPL31,RPS27A,RPS17,RPL6,RPS24,RPS15A,RPS21,RPS13,NUP93,RPL27,KPNB1,RPSA,RPLP2,RPL35A,NUP62,RPS18,RPL36,RPL39,RPL23AP42,RPS15AP11,RPLP0,POLR2E,FAU,POLR2D,RPL5P1,RPL7AP66,RPL26,NUP153,RPS26,POLR2L,RPL21P134,RPL38,RPS19,RPS15P5,RPS19P3,NUP188,RPL41,RPL23AP74* |
| Reactome: SRP-dependent cotranslational protein targeting to membrane | 127 | 0.004 | -2.87 | *RPS15,RPL18A,RPL28,RPL3,RPS7,RPL12,RPL14,RPL13,RPS3,RPL18,RPS8,RPL19,RPS2,RPL4,RPS9,RPL13A,SSR2,RPL35,RPS4X,RPL30,RPS27,RPS6,RPL15,RPL27A,RPL7,RPS5,RPL7A,RPL32,RPS20,RPL10A,RPL37,RPL24,RPS12,RPS16,RPL23,RPS23,RPLP1,RPL34,RPL5,RPS25,RPL23A,RPL37A,RPS3A,RPL8,RPL29,RPL11,RPL10,RPS11,RPS28,RPS29,RPL21,RPL22,RPS14,RPL9,UBA52,RPS4Y1,RPL23AP18,RPL31,RPS27A,RPS17,RPL6,RPS24,RPS15A,RPS21,RPS13,RPL27,RPSA,RPLP2,RPL35A,RPS18,RPL36,RPL39,RPL23AP42,RPS15AP11,RPLP0,SEC11A,FAU* |
| Reactome: metabolism of mRNA | 227 | 0.004 | -2.52 | *RPS15,RPL18A,PABPC1,RPL28,RPL3,RPS7,RPL12,RPL14,RPL13,RPS3,EIF4B,RPL18,RPS8,RPL19,RPS2,RPL4,RPS9,RPL13A,RPL35,RPS4X,RPL30,RPS27,RPS6,RPL15,RPL27A,RPL7,RPS5,RPL7A,RPL32,RPS20,RPL10A,DCP1A,RPL37,RPL24,RPS12,RPS16,RPL23,RPS23,RPLP1,RPL34,TNKS1BP1,RPL5,RPS25,RPL23A,RPL37A,PSME1,RPS3A,RPL8,RPL29,RPL11,RPL10,RPS11,RPS28,RPS29,RPL21,RPL22,RPS14,RPL9,UBA52,RPS4Y1,RPL23AP18,RPL31,RPS27A,RPS17,RPL6,RPS24,RPS15A,RPS21,PSMF1,RPS13,RPL27,HSPB1,PATL1,RPSA,RPLP2,RPL35A,EIF4A2,PSMB9,RPS18,RPL36,RPL39,RPL23AP42,RPS15AP11,PRKCD,HNRNPD,RPLP0,PARN,FAU* |
| Reactome: metabolism of RNA | 272 | 0.004 | -2.41 | *RPS15,RPL18A,PABPC1,RPL28,RPL3,RPS7,RPL12,RPL14,RPL13,RPS3,EIF4B,RPL18,RPS8,RPL19,RPS2,RPL4,RPS9,RPL13A,RPL35,RPS4X,RPL30,RPS27,RPS6,RPL15,RPL27A,RPL7,RPS5,RPL7A,RPL32,RPS20,RPL10A,DCP1A,RPL37,RPL24,RPS12,RPS16,RPL23,RPS23,RPLP1,RPL34,TNKS1BP1,RPL5,RPS25,RPL23A,RPL37A,PSME1,RPS3A,RPL8,RPL29,RPL11,RPL10,RPS11,RPS28,RPS29,RPL21,RPL22,RPS14,RPL9,UBA52,RPS4Y1,RPL23AP18,RPL31,RPS27A,RPS17,RPL6,RPS24,RPS15A,RPS21,PSMF1,RPS13,NUP93,RPL27,HSPB1,PATL1,RPSA,RPLP2,RPL35A,EIF4A2,SNRPD2,NUP62,PSMB9,RPS18,RPL36,RPL39,RPL23AP42,RPS15AP11,PRKCD,HNRNPD,RPLP0,PARN,FAU,RPL5P1,RPL7AP66,PSMD8,DCP1B,RPL26,NUP153,PSMB10,RPS26* |
| Reactome: smooth muscle contraction | 23 | 0.004 | -2.22 | *ITGA1,MYLK,MYL9,LMOD1,ACTG2,VCL,TLN1,TPM2,TPM4,MYL6,CALM3,MYH11,CALM1,MYL12B,CALD1,MYL6B,CALM2* |

# Table E3. Gene set enrichment analysis results corresponding to the TNFα versus control comparisons.

Significantly enriched pathways (q-value <0.05, normalized enrichment score (NES) >2) corresponding to the gene set enrichment analyses obtained based on the genes differential expressed for the TNFα versus control comparisons within each of the two siRNA conditions (i.e., *CEBPD*, NT) are shown. Genes in Leading Edge are ordered based on their absolute t-statistics. BUD: budesonide; NT: non-targeting.

| **Pathway** | **Size** | **q-value** | **NES** | **Genes in Leading Edge** |
| --- | --- | --- | --- | --- |
| **TNFα versus control among cells transfected with NT siRNA** | | | | |
| Reactome: peptide chain elongation | 104 | 0.004 | -2.81 | *RPS15A, RPS9, RPL11, RPL19, RPL21P134, RPL29, RPL15, RPL32, RPL24, RPS15, RPL5P1, RPS28P7, RPL30, RPS20, RPS7, RPL7AP66, RPL6, RPS15AP11, RPL34, RPL4, RPSAP12, RPS25, RPL13, RPS18, RPL21, RPL37, RPL10, RPL12, RPS19, RPL8, RPS5, RPL37A, RPS15P5, RPL13A, RPL23, RPL10A, RPS16, RPS4X, RPLP0, RPL7A, RPL35A, RPL27, RPLP2, RPS14, RPL31, RPS23, RPS8, RPL14, RPL9, RPS2, RPS6, RPL5, RPS10, RPS21, RPL22, RPS3, RPS28, RPS29, RPL7, RPL18, RPL27A, RPS17, EEF2, RPS12, RPS3A, RPL26, RPL3, RPSA* |
| Reactome: 3' UTR mediated translational regulation | 125 | 0.004 | -2.78 | *RPLP1,RPS4Y1,RPS27,RPSAP9,RPS24,RPS26,RPL38,RPL36,RPL18A,RPL17,EIF2S3,RPL41,RPL21P119,PABPC1,UBA52,RPS13,EIF3K,RPS15A,RPS9,RPL11,RPL19,RPL21P134,RPL29,RPL15,RPL32,RPL24,EIF4B,RPS15,RPL5P1,EIF3H,RPS28P7,RPL30,RPS20,RPS7,EIF4A2,RPL7AP66,RPL6,RPS15AP11,RPL34,RPL4,RPSAP12,EIF3E,RPS25,RPL13,RPS18,RPL21,RPL37,EIF3FP3,RPL10,RPL12,EIF2S2,RPS19,RPL8,RPS5,RPL37A,RPS15P5,RPL13A,RPL23,RPL10A,RPS16,RPS4X,RPLP0,RPL7A,RPL35A,RPL27,RPLP2,RPS14,RPL31,RPS23,EIF3C,RPS8,RPL14,RPL9,RPS2,RPS6,RPL5,RPS10,RPS21,RPL22,RPS3,RPS28,RPS29,RPL7,RPL18,RPL27A,RPS17,RPS12,RPS3A,RPL26,RPL3,RPSA,EIF3F* |
| KEGG: ribosome | 87 | 0.004 | -2.78 | *RPLP1, RPS4Y1, RPS27, RPS24, RPS26, RPL38, RPL36, RPL18A, RPL17, RSL24D1, RPL22L1, RPL41, UBA52, RPS13, RPS15A, RPS9, RPL11, RPL19, RPL29, RPL15, RPL32, RPL24, RPS15, MRPL13, RPL30, RPS20, RPS7, RPL6, RPL34, RPL4, RPS25, RPL13, RPS18, RPL21, RPL37, RPL10, RPL12, RPS19, RPL8, RPS5, RPL37A, RPL13A, RPL23, RPL10A, RPS16, RPS4X, RPLP0, RPL7A, RPL35A, RPL27, RPLP2, RPL31, RPS23, RPS8, RPL14, RPL9, RPS2, RPS6, RPL5, RPS10, RPS21, RPL22, RPS3, RPS28, RPS29, RPL7, RPL18, RPL27A, RPS17, RPS12, RPS3A, RPL26, RPL3, RPSA* |
| Reactome: formation of the ternary complex and subsequently the 43s complex | 56 | 0.003 | -2.46 | *RPS4Y1, RPS27, RPSAP9, RPS24, RPS26, EIF2S3, RPS13, EIF3K, RPS15A, RPS9, RPS15, EIF3H, RPS28P7, RPS20, RPS7, RPS15AP11, RPSAP12, EIF3E, RPS25, RPS18, EIF3FP3, EIF2S2, RPS19, RPS5, RPS15P5, RPS16, RPS4X, RPS14, RPS23, EIF3C, RPS8, RPS2, RPS6, RPS10, RPS21, RPS3, RPS28, RPS29, RPS17, RPS12, RPS3A, RPSA, EIF3F* |
| Reactome: glycolysis | 23 | 0.002 | 2.00 | *PFKFB4, ENO2, PFKFB3, PPP2R1B, TPI1, ENO1, PFKP, PGAM1, PGK1, GAPDH, ALDOA, GPI, TPI1P1, ALDOC, PFKL* |
| KEGG: JAK-STAT signaling pathway | 98 | 0.002 | 2.01 | *IL6, IL7R, CSF2, LIF, CSF3, IL15RA, IFNAR2, IL13RA2, IFNAR1, PIK3CD, IFNGR2, SOCS2, STAT4, IL11, IL4R, IL24, SPRY2, JAK1, STAT1, AKT3, STAT2, SPRY4, STAT5A, STAT5B, STAT6, TYK2, EP300, AKT1, IRF9, OSMR, PRLR, LEPR, IFNGR1, LEP, IL12RB1, PIK3CB, IL15, CNTF, CCND3* |
| Reactome: chemokine receptors bind chemokines | 24 | 0.002 | 2.03 | *CXCL2, CXCL11, CCL7, CXCL5, CXCL6, CXCL3, CCL2, CXCL1, CCL11, CCL20, CXCL10, CCL5, CXCR4, CXCL16, CXCL9* |
| Reactome: antigen presentation class I MHC | 21 | 0.002 | 2.04 | *ERAP1, HLA-F, HLA-B, TAP2, HLA-A, B2M, TAP1, CANX, HLA-C, PDIA3, HSPA5, CALR, SEC24D* |
| KEGG: hematopoietic cell lineage | 44 | 0.002 | 2.04 | *IL6, IL7R, IL1B, MME, CSF2, IL1A, CSF1, ITGA2, CSF3, ITGA1, ANPEP, IL11, IL4R, ITGA5, ITGB3, CD34, CD55, CD38* |
| Reactome: TAK1 activates NFkB by phosphorylation and activation of IKKs complex | 21 | 0.002 | 2.06 | *NFKB2, NFKBIA, IRAK2, RIPK2, TAB3, RELA, NFKBIB, TAB2, NOD2, IKBKB* |
| KEGG: B cell receptor signaling pathway | 55 | 0.002 | 2.08 | *NFKBIA, NFKBIE, RASGRP3, NFKB1, PPP3CC, PIK3CD, MAP2K1, IFITM1, PIK3AP1, RELA, NFKBIB, AKT3, NFATC2, JUN, LYN, IKBKB, AKT1, NFAT5* |
| KEGG: apoptosis | 75 | 0.002 | 2.09 | *NFKBIA, IL1B, IRAK2, IL1A, BIRC3, BIRC2, IRAK3, TRAF2, NFKB1, BID, PPP3CC, PIK3CD, TNFRSF10B, CFLAR, RELA, FAS, AKT3, MYD88, CASP7, TNFRSF10A, IKBKB, AKT1, PRKAR1A, IL1RAP, CASP3, PRKAR2B, APAF1, PIK3CB, XIAP, CYCS, FADD, ENDOD1, TNFSF10* |
| KEGG: graft versus host disease | 16 | 0.002 | 2.11 | *IL6, IL1B, IL1A, HLA-F, HLA-B, HLA-A, HLA-C, HLA-E, FAS* |
| Reactome: antigen processing cross presentation | 66 | 0.002 | 2.13 | *CTSS, PSME2, HLA-F, HLA-B, TAP2, PSME1, PSMB9, HLA-A, B2M, PSMB8, TAP1, ITGAV, PSMB10, HLA-C, PSMB7, PDIA3, SEC61G, CALR, PSMA4, PSMA6, PSMA3, PSMA5, PSMB2, PSMD3, PSMC1, NCF2, PSME4, PSMD2, PSMD14, PSMD12* |
| Reactome: activation of nf kappab in B cells | 58 | 0.002 | 2.14 | *NFKBIA, NFKBIE, PSME2, PSME1, PSMB9, PSMB8, REL, PSMB10, RELA, NFKBIB, PSMB7, IKBKB, CUL1, PSMA4, PSMA6, PSMA3, PSMA5, PSMB2, PSMD3, PSMC1, PSME4, PSMD2, PSMD14, CHUK, BCL10, PSMD12, MALT1* |
| KEGG: RIG-I-like receptor signaling pathway | 51 | 0.002 | 2.19 | *NFKBIA,IRF7,TRAF3,TRAF2,CYLD,NFKB1,IKBKE,ISG15,DHX58,CXCL10,TRIM25,IFIH1,DDX58,RELA,NFKBIB,TBK1,MAPK8,TANK,IKBKB,MAPK13* |
| KEGG: antigen processing and presentation | 45 | 0.002 | 2.20 | *CTSS, PSME2, HLA-F, HLA-B, TAPBP, TAP2, PSME1, HLA-A, B2M, TAP1, RFX5, CD74, CANX, HLA-C, HLA-E, PDIA3, HSPA5* |
| KEGG: cytosolic DNA sensing pathway | 38 | 0.002 | 2.24 | *IL6, NFKBIA, IL1B, IRF7, NFKB1, IL33, IKBKE, CXCL10, CCL5, ADAR, TREX1, DDX58, RELA, NFKBIB, TBK1, POLR3D, IKBKB, POLR3A, CASP1* |
| Reactome: innate immune system | 184 | 0.002 | 2.28 | *NFKB2, NFKBIA, IRAK2, CTSS, RIPK2, TNFAIP3, BIRC3, PANX1, IRF7, BIRC2, TRAF3, IRAK3, TRAF2, CYLD, IKBKE, TAB3, MAP2K1, HERC5, IRF1, ISG15, PPP2R1B, UBE2L6, DHX58, TRIM25, IFIH1, C3, TICAM1, TLR2, NLRC5, DDX58, MAP2K3, CTSK, HSP90B1, CFB, RELA, NFKBIB, IRF2, CFH, TLR1, DUSP3, UNC93B1, TBK1, MYD88, UBA7, RPS6KA1, DUSP6, TAB2, PIK3C3, JUN, TLR3, CASP4, MAPK8, RPS6KA3, EP300, CD55, TANK, NOD2, IKBKB, MAPK13, CD46* |
| KEGG: cytokine cytokine receptor interaction | 157 | 0.002 | 2.30 | *IL6, IL7R, IL1B, CXCL2, CSF2, LIF, CXCL11, IL1A, CCL7, INHBA, CXCL5, CSF1, CSF3, IL15RA, IFNAR2, CXCL6, TNFRSF1B, CXCL3, IFNAR1, CCL2, CXCL1, VEGFC, IFNGR2, CCL11, TNFSF13B, CCL20, CXCL10, CCL5, TNFRSF10B, BMP2, TNFSF15, IL11, TNFRSF9, TNFSF4, IL18R1, IL4R, TNFSF18, IL24, FAS, PF4V1, CXCR4, EGFR, TGFBR1, TNFRSF10A, TNFRSF12A, VEGFA, CXCL16, CXCL9, TNFRSF21, TNFRSF8, TNFRSF11B, CCL13, TNFRSF14, KDR, IL17RB, OSMR, PRLR, LEPR, RELT, IFNGR1, TNFRSF18, PDGFC, IL1RAP, CCL8, LEP, VEGFB, IL12RB1, BMPR1B* |
| KEGG: NOD-like receptor signaling pathway | 56 | 0.002 | 2.37 | *IL6, NFKBIA, IL1B, CXCL2, RIPK2, CCL7, TNFAIP3, BIRC3, BIRC2, NFKB1, CCL2, CXCL1, TAB3, CCL11, CCL5, HSP90B1, RELA, NFKBIB, CARD6, TAB2, MAPK8, CCL13, NOD2, IKBKB, MAPK13* |
| KEGG: toll like receptor signaling pathway | 75 | 0.002 | 2.41 | *IL6, NFKBIA, IL1B, CXCL11, IRF7, TRAF3, IFNAR2, NFKB1, IFNAR1, IKBKE, PIK3CD, MAP2K1, CXCL10, CCL5, TICAM1, TLR2, MAP2K3, CTSK, RELA, STAT1, TLR1, AKT3, TBK1, MYD88, MAP3K8, TAB2, JUN, CXCL9, TLR3, MAPK8, IKBKB, MAPK13, AKT1* |
| Reactome: RIG-I/MDA5 mediated induction of ifn alpha beta pathways | 56 | 0.002 | 2.42 | *NFKB2, NFKBIA, TNFAIP3, IRF7, TRAF3, TRAF2, CYLD, IKBKE, HERC5, IRF1, ISG15, UBE2L6, DHX58, TRIM25, IFIH1, NLRC5, DDX58, RELA, NFKBIB, IRF2, TBK1, UBA7, EP300, TANK, IKBKB* |
| Reactome: interferon alpha beta signaling | 48 | 0.002 | 2.44 | *IFI27, IRF7, HLA-F, IFNAR2, HLA-B, OAS2, IFNAR1, OAS1, OAS3, MX1, HLA-A, IFI6, IRF1, ISG15, MX2, GBP2, ADAR, IFIT1, PSMB8, IFI35, IFITM1, IFIT3, XAF1, OASL, HLA-C, JAK1, STAT1, IRF2, STAT2, IFIT2, TYK2, ISG20, USP18* |
| Reactome: interferon gamma signaling | 49 | 0.002 | 2.44 | *ICAM1, IRF7, HLA-F, VCAM1, HLA-B, OAS2, OAS1, OAS3, HLA-A, IFNGR2, IRF1, SP100, B2M, GBP2, PML, GBP5, GBP4, GBP1, MT2A, OASL, HLA-C, PRKCD, JAK1, STAT1, IRF2, PTPN2* |
| Reactome: cytokine signaling in immune system | 222 | 0.002 | 2.50 | *IL6, NFKB2, ICAM1, IL7R, IL1B, IRAK2, CSF2, RIPK2, IL1A, IFI27, IRF7, HLA-F, IRAK3, IFNAR2, VCAM1, HLA-B, OAS2, IFNAR1, OAS1, OAS3, MX1, HLA-A, PIK3CD, TAB3, IFI6, MAP2K1, HERC5, IFNGR2, IRF1, ISG15, MX2, SP100, B2M, GBP2, UBE2L6, SOCS2, ADAR, IFIT1, PSMB8, PML, TRIM25, GBP5, GBP4, IFI35, SQSTM1, IFITM1, NUP62, IFIT3, GAB2, XAF1, NEDD4, GBP1, MT2A, DDX58, OASL, HLA-C, PRKCD, UBE2E1, RELA, EIF2AK2, JAK1, STAT1, IRF2, PTPN2, STAT2, MYD88, IL1RN, CRKL, UBA7, STAT5A, MAP3K8, TAB2, IFIT2, STAT5B, TYK2, ISG20, USP18, LYN, NOD2, IKBKB, TPR, IRF9, KPNA2, CUL1, PRLR* |
| Reactome: interferon signaling | 129 | 0.002 | 2.53 | *ICAM1, IFI27, IRF7, HLA-F, IFNAR2, VCAM1, HLA-B, OAS2, IFNAR1, OAS1, OAS3, MX1, HLA-A, IFI6, HERC5, IFNGR2, IRF1, ISG15, MX2, SP100, B2M, GBP2, UBE2L6, ADAR, IFIT1, PSMB8, PML, TRIM25, GBP5, GBP4, IFI35, IFITM1, NUP62, IFIT3, XAF1, NEDD4, GBP1, MT2A, DDX58, OASL, HLA-C, PRKCD, UBE2E1, EIF2AK2, JAK1, STAT1, IRF2, PTPN2, STAT2, UBA7, IFIT2, TYK2, ISG20, USP18, TPR, IRF9, KPNA2* |
| **TNFα versus control among cells transfected with *CEBPD* siRNA** | | | | |
| KEGG: ribosome | 86 | 0.003 | -2.44 | *RPL29, RPL38, RPS21, RPS26, RPL26, RPL19, RPS7, RPS16, RPS5, RPLP2, RPL35, RPL13, RPL11, RPL12, RPS28, RPS24, RPL18, RPS20, RPL8, RPL37A, RPL24, RPL41, RPL37, RPL30, RPL27, RPS2, RPL32, RPL6, RPL13A, RPL14, RPL35A, RPS18, RPL34, RPS4Y1, RPL10A, RPS29, RPL15, RPS17, RPS25, RPS15A, RPS8, RPL21, RPLP0, RPS12, RPS13, RPL9, RPS3, RPL7A, RPL31, RPL4, RPL23, RPL22, RPL3, RPS6, RPS4X, RPSA, RPL27A, RPS23, RPL5, RPS3A, RPL7* |
| Reactome: peptide chain elongation | 103 | 0.003 | -2.40 | *RPL29,RPL38,RPS21,RPS26,RPL26,RPL19,RPS7,RPS16,RPS5,RPLP2,RPL35,RPL13,RPL11,RPL12,RPS28,RPL5P1,RPL21P134,RPS24,RPL18,RPS20,RPS14,RPL8,RPL37A,RPL24,RPL41,RPL37,RPSAP9,RPL30,RPL27,RPS2,RPL32,RPL6,RPL13A,RPSAP12,RPL14,RPL35A,RPS18,RPL34,RPS4Y1,RPL10A,RPS29,RPL15,RPS17,EEF2,RPS25,RPS15A,RPS8,RPL21,RPLP0,RPS12,RPS13,RPL9,RPS3,RPL7A,RPL31,RPL4,RPL23,RPL22,RPL3,RPS6,RPS4X,RPSA,RPL27A,RPS23,RPL5,RPS3A,RPL7* |
| Reactome: 3' UTR mediated translational regulation | 124 | 0.003 | -2.40 | *RPL29, RPL38, EIF2S3, RPS21, RPS26, RPL26, RPL19, RPS7, RPS16, RPS5, RPLP2, RPL35, RPL13, RPL11, RPL12, RPS28, PABPC1, RPL5P1, EIF2S2, RPL21P134, RPS24, RPL18, RPS20, RPS14, RPL8, RPL37A, RPL24, RPL41, RPL37, RPSAP9, EIF3H, RPL30, EIF3E, EIF3C, RPL27, RPS2, RPL32, RPL6, RPL13A, RPSAP12, RPL14, RPL35A, RPS18, RPL34, EIF4B, RPS4Y1, RPL10A, RPS29, RPL15, RPS17, EIF1AX, RPS25, RPS15A, RPS8, RPL21, RPLP0, RPS12, RPS13, EIF4A2, RPL9, RPS3, RPL7A, RPL31, RPL4, RPL23, RPL22, RPL3, RPS6, RPS4X, RPSA, RPL27A, RPS23, RPL5, RPS3A, EIF3F, RPL7* |
| Reactome: cytosolic tRNA aminoacylation | 24 | 0.003 | -2.16 | *NARS, AIMP1, VARS, CARS, MARS, LARS, FARSB, PPA1, AARS, YARS, EPRS, IARS, SARS, GARS* |
| Reactome: formation of the ternary complex and subsequently the 43S complex | 56 | 0.003 | -2.15 | *EIF3G,RPS27,EIF3FP3,RPS26P28,RPS10,RPS27A,EIF3I,EIF3J,RPS19P3,EIF2S3,RPS21,RPS26,RPS7,RPS16,RPS5,RPS28,EIF2S2,RPS24,RPS20,RPS14,RPSAP9,EIF3H,EIF3E,EIF3C,RPS2,RPSAP12,RPS18,RPS4Y1,RPS29,RPS17,EIF1AX,RPS25,RPS15A,RPS8,RPS12,RPS13,RPS3,RPS6,RPS4X,RPSA,RPS23,RPS3A,EIF3F* |
| Reactome: amino acid synthesis and interconversion transamination | 15 | 0.004 | -2.07 | *GLS, GOT1, PSPH, PYCR1, PHGDH, ASNS, PSAT1* |
| KEGG: graft versus host disease | 16 | 0.002 | 2.01 | *IL6, IL1A, IL1B, HLA-A, HLA-B, HLA-F, HLA-C, FAS, HLA-E* |
| Reactome: signaling by the B cell receptor BCR | 114 | 0.002 | 2.04 | *NFKBIA, NFKBIE, RASGRP3, PSME2, PSME1, PSMB9, PIK3CD, NFKBIB, RICTOR, ITPR2, PSMB8, REL, IKBKB, RELA, ORAI1, NCK1, PSMB7, PSMB10, AKT3, FYN, AKT1S1, LYN, PSMA6, PSMA4, ITPR3, GSK3A, PSMA3, NR4A1, AKT1, PSMD3, FOXO1, FOXO3, MALT1, PSMA5, SHC1, PSMD2, BAD, PSMC4, PSMB4, PIK3AP1, CUL1* |
| KEGG: hematopoietic cell lineage | 43 | 0.002 | 2.04 | *IL6, IL7R, IL1A, MME, CSF2, CSF3, CSF1, ITGA2, IL1B, ANPEP, IL4R, IL11, CD55, ITGA5, ITGA1, ITGB3, TFRC, CD34* |
| Reactome: immunoregulatory interactions between a lymphoid and a non-lymphoid cell | 32 | 0.002 | 2.05 | *ICAM1, VCAM1, C3, HLA-A, HLA-B, B2M, HLA-F, HLA-C, IFITM1, PVRL2, CD34, ICAM2, CXADR, ULBP2* |
| KEGG: B cell receptor signaling pathway | 58 | 0.002 | 2.05 | *NFKBIA, NFKB1, NFKBIE, RASGRP3, MAP2K1, PIK3CD, NFKBIB, PPP3CC, IKBKB, RELA, NFATC2, IFITM1, AKT3, LYN, JUN, AKT1, MAP2K2, MALT1* |
| Reactome: TAK1 activates NFkB by phosphorylation and activation of IKKs complex | 20 | 0.002 | 2.10 | *NFKB2, NFKBIA, IRAK2, RIPK2, TAB3, NFKBIB, IKBKB, RELA, TAB2* |
| KEGG: antigen processing and presentation | 46 | 0.002 | 2.11 | *CTSS, PSME2, TAPBP, PSME1, TAP2, HLA-A, HLA-B, TAP1, B2M, HLA-F, RFX5, HLA-C, CANX, CD74, PDIA3, HSPA5, HLA-E, HSPA1A, CALR* |
| Reactome: chemokine receptors bind chemokines | 24 | 0.002 | 2.11 | *CXCL2, CXCL3, CXCL5, CXCL6, CCL20, CXCL1, CCL2, CCL7, CXCR4, CXCL10, CXCL11, CCL5, CXCL16* |
| Reactome: activation of NF-kappab in B cells | 59 | 0.002 | 2.13 | *NFKBIA,NFKBIE,PSME2,PSME1,PSMB9,NFKBIB,PSMB8,REL,IKBKB,RELA,PSMB7,PSMB10,PSMA6,PSMA4,PSMA3,PSMD3,MALT1,PSMA5,PSMD2,PSMC4,PSMB4,CUL1* |
| Reactome: glycolysis | 23 | 0.002 | 2.14 | *PFKFB4, ENO2, PFKFB3, TPI1, ENO1, PFKP, PGK1, PGAM1, ALDOA, PPP2R1B, GAPDH, GPI, TPI1P1, PFKL, ALDOC* |
| KEGG: apoptosis | 76 | 0.002 | 2.16 | *NFKBIA, IL1A, BIRC3, IRAK2, BIRC2, NFKB1, IRAK3, TRAF2, BID, IL1B, PIK3CD, TNFRSF10B, PPP3CC, CFLAR, IKBKB, RELA, AKT3, PRKAR1A, FAS, PRKAR2B, CASP7, TRADD, XIAP, AKT1, TNFSF10, CASP3, MYD88, ENDOD1, IL1RAP, BCL2L1, BAD, TNFRSF10A* |
| Reactome: NOD1/2 signaling pathway | 29 | 0.002 | 2.16 | *TNFAIP3, BIRC3, IRAK2, RIPK2, BIRC2, CYLD, TAB3, IKBKB, CASP4, CASP1, MAPK13, TAB2* |
| Reactome: antigen processing cross presentation | 66 | 0.002 | 2.20 | *CTSS, PSME2, PSME1, TAP2, PSMB9, HLA-A, HLA-B, TAP1, B2M, HLA-F, PSMB8, HLA-C, PSMB7, ITGAV, PSMB10, PDIA3, SEC61G, PSMA6, PSMA4, PSMA3, PSMD3, CALR, PSMA5, PSMD2, PSMC4, PSMB4, CYBA, SEC61B* |
| KEGG: JAK-STAT signaling pathway | 97 | 0.002 | 2.28 | *IL6, IL7R, LIF, IL13RA2, CSF2, CSF3, IL15RA, IFNAR1, IFNGR2, IFNAR2, IL24, PIK3CD, IL4R, SOCS2, IL11, SPRY2, TYK2, IFNGR1, STAT4, STAT1, IL21R, JAK1, OSMR, AKT3, IRF9, IL15, STAT5A, STAT6, STAT5B, PRLR, STAT2, AKT1, SPRY4, IL10RB, CCND3, CNTF, BCL2L1, SPRY3, EPOR, EP300* |
| KEGG: RIG-I-like receptor signaling pathway | 51 | 0.002 | 2.30 | *NFKBIA, NFKB1, TRAF2, TRAF3, IKBKE, CYLD, TRIM25, ISG15, IFIH1, CXCL10, NFKBIB, IRF7, IKBKB, RELA, TBK1, DHX58, DDX58, TANK, MAPK13, MAPK8, DDX3Y, AZI2, TRADD* |
| KEGG: cytosolic DNA sensing pathway | 37 | 0.002 | 2.32 | *IL6, NFKBIA, NFKB1, IL1B, IKBKE, IL33, CXCL10, NFKBIB, CCL5, ADAR, IRF7, IKBKB, RELA, TBK1, DDX58, TREX1, CASP1* |
| Reactome: innate immune system | 184 | 0.002 | 2.34 | *TNFAIP3, NFKB2, NFKBIA, CTSS, BIRC3, IRAK2, RIPK2, BIRC2, IRAK3, TRAF2, TRAF3, IKBKE, PANX1, C3, MAP2K1, CYLD, CTSK, NLRC5, TAB3, TRIM25, TICAM1, ISG15, IFIH1, NFKBIB, IRF1, CFB, IRF7, IKBKB, TLR2, RELA, TBK1, DHX58, MAP2K3, DDX58, DUSP6, IRF2, TLR1, PPP2R1B, CD55, CASP4, TANK, CASP1, HERC5, CFH, UNC93B1, MAPK13, HSP90B1, MAPK8, UBE2L6, MAPKAPK2, TAB2, UBE2D3, JUN, TLR3, RPS6KA1, ELK1, CD46, MYD88, RPS6KA3, DDOST, CNPY3, UBA7, PIK3C3, NOD2, PRKCSH, MAP2K2* |
| KEGG: toll like receptor signaling pathway | 76 | 0.002 | 2.36 | *IL6, NFKBIA, NFKB1, TRAF3, IFNAR1, IL1B, IKBKE, MAP2K1, CTSK, IFNAR2, TICAM1, PIK3CD, CXCL10, CXCL11, CCL5, MAP3K8, IRF7, IKBKB, TLR2, RELA, TBK1, MAP2K3, STAT1, TLR1, AKT3, MAPK13, MAPK8, TAB2, JUN, TLR3, AKT1, MYD88* |
| KEGG: cytokine cytokine receptor interaction | 153 | 0.002 | 2.39 | *IL6, IL7R, LIF, IL1A, CXCL2, CSF2, CXCL3, CXCL5, CSF3, CSF1, CXCL6, CCL20, IL15RA, CXCL1, CCL2, IFNAR1, IL1B, IFNGR2, CCL7, IFNAR2, VEGFC, IL24, VEGFA, CXCR4, TNFRSF1B, INHBA, IL18R1, IL4R, CXCL10, TNFRSF9, TNFSF4, TNFRSF10B, CXCL11, CCL5, TNFRSF12A, TNFSF15, BMP2, IL11, CXCL16, IFNGR1, TNFSF18, IL21R, OSMR, CCL13, RELT, IL15, CCL8, TNFRSF21, TNFRSF18, PF4V1, PRLR, FAS, PDGFC, HGF, TNFRSF8, TNFSF13B* |
| Reactome: interferon gamma signaling | 49 | 0.002 | 2.43 | *ICAM1, VCAM1, OAS3, IFNGR2, HLA-A, MT2A, OAS1, OAS2, HLA-B, B2M, HLA-F, IRF1, HLA-C, IRF7, GBP5, GBP4, IFNGR1, SP100, IRF2, GBP1, STAT1, JAK1, PTPN2, IRF9, PML, PTPN1, OASL, PRKCD, GBP2* |
| KEGG: NOD-like receptor signaling pathway | 54 | 0.002 | 2.47 | *IL6, TNFAIP3, NFKBIA, CXCL2, BIRC3, RIPK2, BIRC2, NFKB1, CXCL1, CCL2, IL1B, CCL7, TAB3, NFKBIB, CCL5, IKBKB, RELA, CCL13, CASP1, MAPK13, CARD6, HSP90B1, MAPK8, CCL8, TAB2* |
| Reactome: RIG-I/MDA5 mediated induction of ifn alpha beta pathways | 55 | 0.002 | 2.50 | *TNFAIP3, NFKB2, NFKBIA, TRAF2, TRAF3, IKBKE, CYLD, NLRC5, TRIM25, ISG15, IFIH1, NFKBIB, IRF1, IRF7, IKBKB, RELA, TBK1, DHX58, DDX58, IRF2, TANK, HERC5, UBE2L6, UBE2D3* |
| Reactome: interferon alpha beta signaling | 48 | 0.002 | 2.51 | *IFI6, IFNAR1, OAS3, HLA-A, IFNAR2, OAS1, MX1, IFI27, OAS2, ISG15, HLA-B, MX2, HLA-F, IRF1, PSMB8, HLA-C, IFIT1, ADAR, XAF1, IRF7, ISG20, TYK2, IFIT3, IRF2, STAT1, IFI35, IFITM1, JAK1, IRF9, USP18, PTPN1, OASL, IFIT2, GBP2, STAT2* |
| Reactome: interferon signaling | 129 | 0.002 | 2.53 | *ICAM1, VCAM1, IFI6, IFNAR1, OAS3, IFNGR2, HLA-A, IFNAR2, MT2A, OAS1, MX1, IFI27, TRIM25, OAS2, ISG15, HLA-B, MX2, B2M, HLA-F, IRF1, PSMB8, HLA-C, UBE2E1, IFIT1, ADAR, XAF1, IRF7, NUP62, ISG20, GBP5, TYK2, GBP4, IFNGR1, DDX58, SP100, IFIT3, IRF2, GBP1, STAT1, EIF2AK2, IFI35, IFITM1, JAK1, PTPN2, HERC5, IRF9, USP18, PML, PTPN1, OASL, PRKCD, UBE2L6, IFIT2, GBP2* |
| Reactome: cytokine signaling in immune system | 221 | 0.002 | 2.61 | *IL6, NFKB2, IL7R, IL1A, ICAM1, IRAK2, RIPK2, CSF2, IRAK3, VCAM1, SQSTM1, IFI6, IFNAR1, IL1B, OAS3, MAP2K1, IFNGR2, HLA-A, IFNAR2, MT2A, OAS1, MX1, TAB3, IFI27, TRIM25, OAS2, PIK3CD, ISG15, HLA-B, SOCS2, MX2, B2M, HLA-F, IRF1, PSMB8, HLA-C, UBE2E1, IFIT1, ADAR, XAF1, MAP3K8, IRF7, IKBKB, NUP62, RELA, ISG20, GBP5, TYK2, GAB2, GBP4, IFNGR1, DDX58, SP100, IFIT3, IRF2, IL1RN, GBP1, STAT1, EIF2AK2, IFI35, IFITM1, JAK1, PTPN2, CASP1, HERC5, ADAM17, IRF9, USP18, PML, STAT5A, PTPN1, YES1, OASL, PRKCD, UBE2L6, FYN, LYN, IFIT2, TAB2, GBP2, STAT5B, PRLR, TNIP2, HGF* |

# Table E4. Gene set enrichment analysis results corresponding to budesonide exposure.

Significantly enriched pathways (q-value <0.05, normalized enrichment score (NES) >2) corresponding to the gene set enrichment analyses obtained based on the genes differential expressed for the BUD versus control and BUD+TNFα versus TNFα comparisons within each of the two siRNA conditions (i.e., *CEBPD*, NT) are shown. Genes in Leading Edge are ordered based on their absolute t-statistics. BUD: budesonide; NT: non-targeting.

| **Pathway** | **Size** | **q-value** | **NES** | **Genes in Leading Edge** |
| --- | --- | --- | --- | --- |
| **BUD versus control among cells transfected with NT siRNA** | | | | |
| Reactome: 3' UTR mediated translational regulation | 126 | 0.005 | 2.65 | *EIF3D,EIF4H,RPS13,RPS4X,EIF2S1,RPS7,RPS27A,RPL10A,RPS3A,RPL5,RPL10,RPS17,RPLP0,EIF3B,RPS8,RPS6,RPL13,RPL23,RPL7A,RPL7,RPL34,RPS5,RPL3,RPS3,RPL13A,RPL32,RPS18,RPL4,RPS12,EIF4G1,RPS14,RPL5P1,FAU,RPS16,EIF4B,RPL6,RPS29,PABPC1,RPL29,RPSA,RPS15A,RPS21,RPL37,RPS23,RPL9,RPL31,EIF3E,RPL14,RPL15,EIF2S2,EIF3J,EIF3A,RPS19,RPL27,RPL35,RPL11,EIF2S3,RPL38,RPL18,RPS24,RPL30,EIF1AX,RPL24,RPL26,RPS25,RPS9,RPL37A,RPL21,RPL36,RPS19P3,EIF3G,RPL18A,RPL23A,RPL34P27,RPL23AP18,EIF3F,RPL8,RPS20,RPS15,RPL22,RPLP2,RPL23AP2,RPS28,RPL19* |
| KEGG: ribosome | 87 | 0.005 | 2.58 | *RPS13,RPS4X,RPS7,RPS27A,RPL10A,RPS3A,RPL5,RPL10,RPS17,RPLP0,RPS8,RPS6,RPL13,RPL23,RPL7A,RPL7,RPL34,RPS5,RPL3,RPS3,RPL13A,RPL32,RPL22L1,RPS18,RPL4,RPS12,FAU,RPS16,RPL6,RPS29,RPL29,RPSA,RPS15A,RPS21,RPL37,RPS23,RPL9,RPL31,RPL14,RPL15,RPS19,RPL27,RPL35,RPL11,RPL38,RPL18,RPS24,RPL30,RPL24,RPL26,RPS25,RPS9,RSL24D1,RPL37A,RPL21,RPL36,RPL18A,RPL23A,RPL8,RPS20,RPS15,RPL22,RPLP2,RPS28,RPL19,RPS26,RPL27A,RPS27,RPL12,RPL17,RPS27L,RPS2* |
| Reactome: glucose metabolism | 58 | 0.005 | 2.50 | *PGK1,GYS1,ENO1,TPI1,PGM1,GPI,PFKFB4,PFKP,UGP2,PYGL,ALDOA,TPI1P1,PYGB,GAPDH,PHKA1,PGAM1,PHKA2,PFKL,AGL,ENO2,PGM2,MDH2,ALDOC,PPP2R1A* |
| Reactome: glycolysis | 23 | 0.005 | 2.39 | *PGK1,ENO1,TPI1,GPI,PFKFB4,PFKP,ALDOA,TPI1P1,GAPDH,PGAM1,PFKL,ENO2,ALDOC,PPP2R1A* |
| KEGG: glycolysis gluconeogenesis | 49 | 0.005 | 2.29 | *ADH1B,LDHA,PGK1,ENO1,TPI1,PGM1,GPI,ALDH1B1,PFKP,HK2,ALDOA,GAPDH,PGAM1,ALDH9A1,PFKL,HK1,ADH5,ADH1A,ENO2,PGM2,ACSS1,ALDOC,PDHA1* |
| Reactome: response to elevated platelet cytosolic Ca2 | 65 | 0.005 | 2.24 | *FN1,THBS1,SERPINE1,A2M,ACTN4,ACTN1,VCL,WDR1,ALDOA,TLN1,CAP1,CD36,CD9,ITGB3,PROS1,ABCC4,SERPING1,PFN1,HABP4,CFD,PPIA,CFL1,PPIAP22,CALM1,SRGN* |
| KEGG: starch and sucrose metabolism | 26 | 0.005 | 2.19 | *GYS1,PGM1,GPI,UGP2,HK2,PYGL,PYGB,HK1,AGL,MGAM,PGM2* |
| Reactome: smooth muscle contraction | 24 | 0.005 | 2.14 | *TPM4,ACTG2,VCL,ACTA2,TLN1,TPM2,LMOD1,MYL9,ITGA1,CALD1,MYL6,ITGB5,TPM3,TPM1,SORBS3,CALM1,MYL12B* |
| Reactome: metabolism of RNA | 273 | 0.005 | 2.13 | *MAPK14,TNKS1BP1,HSPB1,RPS13,ETF1,RPS4X,ZFP36,PRKCD,EXOSC7,RPS7,RPS27A,RPL10A,RPS3A,RPL5,GEMIN5,NUP93,RPL10,NUP50,RPS17,RPLP0,RPS8,RPS6,WDR77,RPL13,RPL23,RPL7A,RPL7,EIF4A3,RPL34,RPS5,RPL3,RPS3,RPL13A,RPL32,RPS18,RPL4,RPS12,YWHAB,EIF4G1,RPS14,RPL5P1,FAU,RPS16,PSMA7,EIF4B,RPL6,RPS29,PABPC1,GEMIN4,RPL29,RPSA,PPP2R1A,RPS15A,RPS21,RPL37,SEH1L,RPS23,RPL9,RPL31,RPL14,SNRPD1,RPL15,PSMF1,MAPK11,RPS19,RPL27,RPL35,RPL11,SMG8,SNRPB,RNPS1,RPL38,RANBP2,RPL18,RPS24,PRMT5,POM121,RPL30,PSMA1,CNOT8,RPL24,PSMA5,RPL26,SMG5,RPS25,MAPKAPK2,RPS9,NUP153,RPL37A,SNRPE,RPL21,RPL36,SNRPG,RPS19P3,RPL18A,RPL23A,RPL34P27,RPL23AP18,RPL8,DIS3,NUP43,RPS20,RPS15,PSMC3,RPL22,RPLP2,NUP54,RPL23AP2,EXOSC2,RPS28,RPL19,GEMIN7,EXOSC9,NUP155,SMG1,RPS26,EIF4E,YWHAZ,RPL27A,RBM8A,NUP133,NUP205,RPS27,SNRPD2,PSMD4,RPL7AP66,CNOT2,RPL12,RPL17,DDX20,PPP2R2A,PSMB4,RPS2,AKT1* |
| KEGG: arginine and proline metabolism | 42 | 0.005 | 2.07 | *GLUL,MAOA,ASL,ALDH1B1,P4HA1,SAT1,AMD1,ALDH9A1,CKB,SRM,PYCR2,GLS,MAOB,PYCRL* |
| Reactome: metabolism of proteins | 413 | 0.005 | 1.81 | *GCNT1,TUBB6,ARSJ,ACTB,VDAC1,TUBB2A,GNPNAT1,GRPEL1,EIF3D,EIF4H,CCT3,CCT5,PROS1,DPM3,EIF5A,B3GNT5,RPS13,ETF1,RPS4X,EIF2B1,EIF2S1,MGAT1,RPS7,RPS27A,TUBA1B,RPL10A,TOMM40,RPS3A,RPL5,TUBA1C,HSPD1,CCT2,NOP56,MPI,RPL10,MGAT5,RPS17,RPLP0,EIF3B,RPS8,RPS6,GFPT2,RPL13,RPL23,RPL7A,RPL7,ALG5,TBCE,EIF5B,PIGA,RPL34,RPS5,RPL3,RPS3,RPL13A,RPL32,MGAT3,RPS18,RPL4,RPS12,EIF4G1,RPS14,SEC61G,TRAM1,RPL5P1,FAU,ARSK,RPS16,EIF4B,RPL6,SSR3,RPS29,TUBB4B,GALNT10,TUBA1A,PABPC1,DAD1,TIMM8A,RPL29,RPSA,RPS15A,RPS21,RPL37,RPS23,GCNT4,PMM2,RPL9,RPL31,EIF3E,RPL14,RPL15,BCS1L,EEF1B2,EIF2S2,ALG2,EIF3J,EIF3A,RPS19,RPL27,RPL35,RPL11,EIF2S3,PIGN,RPL38,SRP19,EIF2B5,RPL18,RPS24,RPL30,EIF1AX,RPL24,MTX1,ST6GALNAC4,RPL26,ALG8,RPS25,RPS9,PREB,B4GALT5,RPL37A,ST6GALNAC3,RPL21,RPL36,SLC25A4,RPS19P3,EIF3G,SEC24B,RPL18A,RPL23A,RPL34P27,RPL23AP18,EIF3F,ATP5G1,RPL8,RPS20,RPS15,TOMM20,C1GALT1C1,TIMM50,DOHH,SEC61B,RPL22,EIF4EBP1,RPLP2,CCT7,PAM16,RPL23AP2,TOMM5,TIMM9,ALG14,KIFC3,PIGG,RPS28,MGAT4B,RPL19,SRPRB* |
| Reactome: biological oxidations | 81 | 0.008 | 1.92 | *MAOA,NNMT,ADH1B,GGT5,PTGS1,UGP2,GSTO1,FMO2,TBXAS1,FMO3,SLC35D1,MAT2A,ADH1A,CYP26C1,ACSS1,MGST1,CYP19A1,AHCY,MAOB,ADH6,PTGIS,CYP26B1,SULT1A1,ALDH2* |
| Reactome: formation of tubulin folding intermediates by CCT/TriC | 18 | 0.024 | 1.88 | *TUBB6,TUBB2A,CCT3,CCT5,TUBA1B,TUBA1C,CCT2,TUBB4B,TUBA1A* |
| Reactome: degradation of the extracellular matrix | 17 | 0.027 | -1.88 | *TIMP2,TIMP1,FURIN,MMP11,MMP17,MMP2,KLKB1,MMP16,MMP1,MMP3,MMP10* |
| Reactome: TCA cycle and respiratory electron transport | 117 | 0.085 | 1.51 | *LDHA,SLC16A3,PDK4,SUCLG2,PDK3,MDH2,PDHA1,DLST,SLC16A1,UQCRC1,COX5B,NDUFB8,NDUFB9,PDK1,COX5A,ATP5F1,IDH3B,ATP5G1,L2HGDH,CYCS,ATP5H,NDUFA9,FH,NDUFA12,ATP5C1,UQCR11,ETFA,SDHD,NDUFS5,ATP5J,SUCLA2P1,ATP5J2,UQCRFS1,PDP1,NDUFC2,NDUFS2,COX8A,UQCRQ,OGDH,LDHB,NDUFS4,COX7A2L,COX6B1,UCP3,PDHB,COX6C,IDH3A,CS,NDUFAB1,NDUFB7,ETFDH,ATP5B,ETFB,NDUFA2,SDHB,SUCLG1,UCP2* |
| Reactome: cytosolic tRNA aminoacylation | 24 | 0.261 | 1.47 | *DARS,FARSB,PPA1,VARS,LARS,TARS,AIMP2,EEF1E1,GARS,HARS,KARS,NARS* |
| Reactome: extracellular matrix organization | 66 | 0.261 | -1.37 | *PLOD3,TIMP2,PPIB,COL25A1,TIMP1,FURIN,SERPINH1,MMP11,MMP17,MMP2,KLKB1,COL11A2,COL13A1,PCOLCE,COL1A2,COL12A1,COL24A1,PCOLCE2,ADAMTS14,COL10A1,MMP16,MMP1,MMP3,MMP10* |
| KEGG: NOD-like receptor signaling pathway | 53 | 0.308 | -1.33 | *RIPK2,CCL2,CASP8,MAPK3,IL6,CASP1,HSP90B1,IL1B,CCL7,NLRP1* |
| Reactome: NCAM1 interactions | 28 | 0.319 | 1.38 | *COL4A1,COL5A2,COL5A1,CACNB2,COL4A2,GDNF,COL4A3,COL4A4,COL3A1,COL6A2,ARTN,COL4A5,COL6A1,COL1A1* |
| KEGG: leishmania Infection | 48 | 0.409 | -1.22 | *TGFB2,JAK2,TGFB3,HLA-DMA,ITGB1,TLR4,MAPK3,HLA-DMB,FCGR2A,IL12A,IL1A,PTGS2,TGFB1,MARCKSL1,IL1B* |
| KEGG: hematopoietic cell lineage | 43 | 0.447 | 1.20 | *ITGA5,IL1R1,ITGA4,ITGA1,CD36,CD9,TFRC,ITGB3,IL6R,MME,IL7R,IL1R2,THPO,IL4R,CSF1* |
| Reactome: signaling by BMP | 21 | 0.551 | -1.11 | *BMPR1A,BMPR2,SMAD7,SKI,UBE2D1,BMP2,ZFYVE16,SMURF1,GREM2* |
| KEGG: cytokine-cytokine receptor interaction | 153 | 0.600 | 1.03 | *TGFBR2,PLEKHO2,TNFRSF1B,CCL20,IL1R1,ACVRL1,CXCL5,PDGFRB,LEP,IL17RA,OSMR,MET,CCL28,TNFRSF19,CCL11,INHBB,IL6R* |
| **BUD versus control among cells transfected with *CEBPD* siRNA** | | | | |
| KEGG: ribosome | 86 | 0.004 | 2.31 | *RPL22L1,RPL36,RPL14,RPS8,RPLP0,RPS2,RPS7,RPL27,RPL35,RPS16,RPS3,RPL7,RPL3,RPS6,RPL29,RPLP1,RPL10A,RPS19,RPS13,RPS21,RPS18,RPL13,RPS27A,RPS12,RPL6,RPL32,RPL8,RPL10,RPL11,RPS3A,RPL5,RPL4,RPL12,RPS5,RPS23,RPSA,RPL34,RPS27,RPL18A,RPLP2,RPL31,RPL7A,RPL13A,RPS15,RPL23A,RPL39,RPS4X,RPS9,RPS4Y1,RPL23,RPL37,RPS29,RPL15,RPL9,RPL24,RPS28,RSL24D1,RPS10,RSL24D1P11,RPS27L,RPS17,RPL36A,RPL26* |
| Reactome: smooth muscle contraction | 24 | 0.004 | 2.22 | *TPM4,ACTA2,VCL,TPM2,LMOD1,MYL9,ACTG2,ITGA1,SORBS3,TLN1,CALD1,TPM1,TPM3* |
| KEGG: glycolysis gluconeogenesis | 42 | 0.004 | 2.17 | *ADH1B,LDHA,PGK1,PGM1,TPI1,ALDH1B1,ENO1,GPI,PFKP,PCK2,ALDOA,ENO2,GAPDH,ACSS1,PFKL,PGAM1,ALDH3B1,HK1,ADH5,HK2,PGM2,ALDH7A1* |
| Reactome: 3' UTR mediated translational regulation | 124 | 0.004 | 2.15 | *EIF1AX,PABPC1,EIF4H,EIF2S3,EIF2S2,RPL36,RPL14,EIF2S1,RPS8,RPLP0,RPS2,RPS7,RPL27,EIF4G1,RPL35,EIF3J,RPS16,RPS3,RPL7,RPL3,RPS6,RPL29,RPLP1,RPL10A,RPS19,RPS13,EIF3E,RPS21,RPS18,RPL13,RPS27A,RPS12,RPL6,RPL32,RPL8,RPL10,RPL11,RPS3A,RPL5,RPL4,RPL12,RPS5,RPS14,EIF3F,RPS23,EIF3C,RPSA,RPL34,EIF3D,RPS27,RPL18A,EIF3B,RPLP2,RPL31,RPL7A,RPL13A,RPS15,RPL23A,RPL22P11,EIF3A,RPL39,RPS4X,EIF3K,RPS9,RPS4Y1,RPL23,RPL37,EIF3G,RPS29,RPL15,RPL9,RPL24,RPS28* |
| Reactome: glucose metabolism | 55 | 0.004 | 2.13 | *PGK1,PYGB,PGM1,TPI1,ENO1,GYS1,GPI,PFKP,PCK2,ALDOA,PHKA1,UGP2,ENO2,GAPDH,PFKFB4,PFKL,PHKA2,PGAM1,SLC25A1,PGM2,PYGL,PYGM,ALDOC,CALM1,PPP2CB,CALM2,ENO3* |
| Reactome: glycolysis | 23 | 0.004 | 2.12 | *PGK1,TPI1,ENO1,GPI,PFKP,ALDOA,ENO2,GAPDH,PFKFB4,PFKL,PGAM1* |
| Reactome: NCAM1 interactions | 28 | 0.004 | 2.09 | *COL5A2,COL5A1,COL3A1,COL1A1,COL4A1,COL6A2,COL6A1,COL4A4,COL1A2,CACNB2,COL4A2,CACNB1,CACNA1G,ARTN,COL6A3* |
| Reactome: cytosolic tRNA aminoacylation | 24 | 0.004 | 2.05 | *FARSB,GARS,AIMP2,PPA1,DARS,YARS,MARS,AARS,KARS,EPRS,TARS,LARS,SARS,CARS,EEF1E1,VARS* |
| Reactome: response to elevated platelet cytosolic Ca2 | 62 | 0.004 | 1.98 | *SERPINE1,FN1,THBS1,ACTN1,WDR1,ACTN4,VCL,ALDOA,TLN1,SPARC,PROS1,CFL1,LAMP2,CAP1,SERPING1,HABP4,CD9,PFN1,CLU,SRGN* |
| Reactome: metabolism of proteins | 408 | 0.004 | 1.64 | *GCNT1,B3GNT5,ACTB,TUBB6,TRAM1,GNPNAT1,ARSJ,EIF1AX,VDAC1,EIF4EBP1,CCT3,PROS1,EIF5A,GCNT4,BCS1L,CCT5,SSR3,PABPC1,EIF4H,EIF2S3,TUBA1B,EIF2S2,GRPEL1,NOP56,TUBB2A,PIGA,RPL36,HSPD1,RPL14,EIF2S1,RPS8,RPLP0,RPS2,RPS7,RPL27,EIF4G1,RPL35,EIF3J,RPS16,TUBA1C,TIMM9,RPS3,PAM16,MAN1A1,TOMM20,MGAT1,FBXW5,CCT8,RPL7,TBCE,RPL3,RPS6,EIF5B,RPL29,RPLP1,C1GALT1C1,RPL10A,SPCS1,RPS19,RPS13,EIF3E,RPS21,TIMM50,STS,RPS18,RPL13,SEC61G,RPS27A,ARSK,CCT6A,EEF1B2,RPS12,RPL6,TIMM8B,TOMM40,PIGL,RPL32,RPL8,RPL10,RPL11,RPS3A,RPL5,ALG2,RPL4,RPL12,RPS5,RPS14,PIGS,EIF3F,TIMM13,RPS23,EIF3C,ALG3,PMM2,TBCB,GRPEL2,MPI,RPSA,RPL34,EIF3D,B4GALT2,DNAJC19,FXN,SRPRB,RPS27,DAD1,EIF2B1,RPL18A,SPCS2,FBXL3,FBXW4,FBXO4,EIF3B,CYC1,RPLP2,HSPA9,RPL31,ETF1,MOGS,RPL7A,RPL13A,CS,EIF2B4,TIMM8A,TOMM22,ALG1,DOHH,TIMM23B,ATP5G1,RPS15,B4GALT6,RPL23A,RFT1,RPL22P11,EIF3A,B3GNT4,EEF2,TBCA,RPL39,RPS4X* |
| Reactome: degradation of the extracellular matrix | 17 | 0.005 | -2.11 | *FURIN,MMP2,MMP16,MMP10,MMP3,MMP1* |
| KEGG: cytokine-cytokine receptor interaction | 145 | 0.007 | -1.91 | *BMPR1A,GDF5,IL21R,CSF2RB,IL24,TNFSF14,IL4R,IFNE,TNFSF18,TNFSF9,TNFRSF8,VEGFA,ACVR1B,IL7,CXCL3,EDA2R,ACVR2B,TNFRSF1A,FIGF,IL1R2,KDR,TNFRSF9,RELT,IL17RB,IFNAR1,TNFRSF25,INHBE,TNFRSF10B,TNFSF13B,IL12A,VEGFB,CSF3,IL13RA1,CLCF1,CXCL1,TNFRSF21,TNFSF10,TSLP,KITLG,TGFB1,TNFSF15,TGFB2,ACVR1,HGF,PDGFRA,TNFRSF11B,CCL2,IL6,TNFRSF10D,CCL7,IL1A,IL1B,IL11,INHBA,CXCL12,LIF* |
| Reactome: metabolism of RNA | 271 | 0.011 | 1.57 | *HSPB1,ZFP36,RNPS1,PABPC1,SEH1L,TNKS1BP1,RPL36,RPL14,SNRPD1,RPS8,RPLP0,RPS2,RPS7,RPL27,EIF4G1,RPL35,RPS16,RPS3,SNRPB,RPL7,RPL3,RPS6,RPL29,RPLP1,MAPK11,WDR77,RPL10A,RPS19,RPS13,MAPK14,RPS21,GEMIN5,RPS18,RPL13,SMG1,RPS27A,RPS12,RPL6,RPL32,RPL8,RPL10,EXOSC8,RPL11,RPS3A,RPL5,RPL4,RPL12,GEMIN2,RPS5,RPS14,YWHAB,RPS23,PSMF1,PSMD9,PRMT5,PSMA7,NUP153,RPSA,RPL34,PSMD8,RPS27,RPL18A,RPLP2,RPL31,ETF1,RPL7A,PHAX,RPL13A,CLNS1A,DCPS,RPS15,MAGOH,YWHAZ,CNOT10,RPL23A,EXOSC7,SNRPG,RPL22P11,NUP93,ZFP36L1,RPL39,RPS4X,LSM5,PSMB9,RPS9,POM121,RPS4Y1,RPL23,RPL37,SNRPD3,HNRNPD,PRKCD,RPS29,TGS1,RPL15,PPP2CA,RPL9,RPL24,RPS28,NUP85,CASC3,SMN2* |
| KEGG: arginine and proline metabolism | 40 | 0.014 | 1.92 | *GLUL,ALDH1B1,MAOA,AMD1,SRM,P4HA1,ODC1,GLS,SAT1,ALDH7A1,PYCR1,P4HA2,P4HA3,MAOB,NAGS,ALDH9A1* |
| KEGG: NOD-like receptor signaling pathway | 52 | 0.028 | -1.78 | *MAPK9,MAP3K7,TRAF6,TRIP6,MAPK10,CXCL1,MAPK3,CASP1,HSP90AA1,NLRP1,HSP90B1,CCL2,IL6,CCL7,IL1B* |
| Reactome: formation of tubulin folding intermediates by CCT/TriC | 18 | 0.032 | 1.87 | *TUBB6,CCT3,CCT5,TUBA1B,TUBB2A,TUBA1C,CCT8,CCT6A* |
| Reactome: biological oxidations | 71 | 0.115 | 1.53 | *ADH1B,NNMT,CYP1B1,GGT5,MAOA,PTGS1,UGP2,GSTO1,CYP11A1,ACSS1,CYP19A1,SLC35D1,MAT2A,TBXAS1,FMO3,FMO2,MGST1,GGCT,CYP7B1,MAOB,AHCY,CYP26B1,CYP39A1* |
| KEGG: starch and sucrose metabolism | 22 | 0.129 | 1.66 | *PYGB,PGM1,GYS1,GPI,UGP2,HK1,HK2,PGM2,PYGL,PYGM* |
| KEGG: leishmania Infection | 48 | 0.140 | -1.57 | *HLA-DMB,ELK1,PTPN6,MAP3K7,FCGR2A,TRAF6,TLR4,IL12A,HLA-DMA,TGFB1,ITGB1,TGFB2,MAPK3,MARCKSL1,IL1A,IL1B,PTGS2* |
| KEGG: hematopoietic cell lineage | 40 | 0.234 | -1.47 | *CSF3,KITLG,CD55,ITGA6,IL6,CD44,IL1A,IL1B,ITGA2,IL11* |
| Reactome: extracellular matrix organization | 64 | 0.416 | 1.26 | *COL5A2,COL5A1,ADAMTS2,COL3A1,COL1A1,COL7A1,COL4A1,COL8A1,COL6A2,COL6A1,COL4A4,COL1A2,COL4A2,COL16A1,TLL1,PLOD1,MMP15,COL6A3,COL27A1,COL5A3* |
| Reactome: TCA cycle and respiratory electron transport | 117 | 0.502 | 1.14 | *LDHA,SLC16A3,PDK4,PDK1,SLC16A1,CYCS,ATP5D,COX5A,UQCRQ,NDUFV1,SDHD,UQCRC1,NDUFB7,NDUFB2,COX5B,SUCLG2,SUCLG1,PDP1,ATP5I,CYC1,PDHA1,CS,COX7C,NDUFAB1,DLST,ATP5G1,IDH3B,ETFA,NDUFB10,NDUFA2,FH,NDUFA5,ATP5C1,NDUFB3,ATP5F1* |
| Reactome: signaling by BMP | 21 | 0.763 | 0.89 | *FSTL1,SMURF2,SMAD4,CHRDL1,SMAD6* |
| **BUD+TNFα versus TNFα among cells transfected with NT siRNA** | | | | |
| Reactome: 3' UTR mediated translational regulation | 125 | 0.005 | 2.91 | *EIF3B,RPS19,RPS5,RPSA,EIF4G1,RPS21,RPS7,RPLP2,RPL10A,RPS17,EIF2S1,RPS29,RPL36,RPL29,RPL26,RPS8,RPS10,RPS15AP11,RPL13A,RPL21P134,RPL3,RPS16,RPS6,RPS3,RPL27,RPL13,RPS28,RPL37A,RPL7A,RPS12,RPL18,RPS2,FAU,RPS15,RPL34,EIF4H,RPS15P5,RPL14,RPL15,RPL23,RPS14,RPL23AP18,RPS3A,EIF3K,RPS27A,RPL35A,RPL12,RPL35,RPL5,RPS25,RPL30,EIF3I,RPL18A,EIF3D,RPLP0,RPL27A,RPL9,RPL4,RPSAP12,EIF3G,RPS15A,RPL8,RPL10,RPS23,RPS19P3,RPL23AP42,EIF3F,RPL5P1,RPL19,RPL32,RPS4Y1,RPS13,RPL31,EIF2S2,UBA52,EIF2S3,EIF4A1,EIF3A,RPS26P28,RPL38,RPL7,RPS18,RPS26,RPL6,RPL17,RPL22,RPS4X,RPS26P35,RPL37,RPS20,RPS9* |
| KEGG: ribosome | 87 | 0.005 | 2.86 | *RPL22L1,RPS19,RPS5,RPSA,RPS21,RPS7,RPLP2,RPL10A,RPS17,RPS29,RPL36,RPL29,RPL26,RPS8,RPS10,RPL13A,RPL3,RPS16,RPS6,RPS3,RPL27,RPL13,RPS28,RPL37A,RPL7A,RPS12,RPL18,RPS2,RPS27L,FAU,RPS15,RPL34,RPL14,RPL15,RPL23,RPS3A,RPS27A,RPL35A,RPL12,RPL35,RPL5,RPS25,RPL30,RPL18A,RPLP0,RPL27A,RPL9,RPL4,RPS15A,RPL8,RPL10,RPS23,RPL19,RPL32,RPS4Y1,RPS13,RPL31,UBA52,RPL38,RPL7,RPS18,RPS26,RPL6,RPL17,RPL22,RPS4X,RPL37,RPS20,RPS9,MRPL13* |
| KEGG: arginine and proline metabolism | 40 | 0.005 | 2.37 | *GLUL,MAOA,ASL,ALDH2,ALDH1B1,MAOB,SRM,PYCRL,AMD1,LAP3,ALDH18A1,ASS1,SAT1,ALDH9A1,P4HA2* |
| KEGG: glycolysis gluconeogenesis | 47 | 0.005 | 2.29 | *LDHA,ENO1,PGK1,ADH1B,TPI1,ALDH2,ALDH1B1,PFKP,ADH5,GPI,HK1,PGM1,PGAM1,ADH1A,GAPDH,PFKL,PGAM4,ALDH9A1,PDHA1,ALDOA,ACSS1,ALDH3B1,LDHB* |
| Reactome: formation of tubulin folding intermediates by CCT/TriC | 18 | 0.005 | 2.19 | *TUBB6,TUBB2A,TUBB4B,TUBA1B,TUBA1C,CCT3,CCT5,CCT7,TUBA1A,CCT2,CCT8,TCP1,CCT6A,TUBB4A,CCT4* |
| Reactome: glucose metabolism | 58 | 0.005 | 2.15 | *UGP2,ENO1,PGK1,TPI1,PYGB,TPI1P1,PFKP,PHKA1,GPI,PGM1,PGAM1,GYS1,GAPDH,SLC25A1,PFKL,PHKA1P1,MDH2,PYGL,ALDOA,GBE1,PPP2R1A,PCK2,PFKM,CALM3* |
| Reactome: extracellular matrix organization | 68 | 0.005 | -2.12 | *COL1A2,COL6A2,COL6A1,MMP3,MMP14,COL11A1,COL10A1,COL6A3,MMP2,MMP9,ADAMTS14,COL13A1,MMP16,COL5A3,COL7A1,COL12A1,MMP1,MMP10* |
| Reactome: smooth muscle contraction | 24 | 0.005 | 2.12 | *TPM4,TPM2,MYL9,MYL12B,ACTA2,VCL,MYL6,TLN1,LMOD1,ACTG2,MYLK,TPM3,CALD1,SORBS3,CALM3,ITGB5,MYH11,CALM1,SORBS1* |
| Reactome: glycolysis | 23 | 0.005 | 2.11 | *ENO1,PGK1,TPI1,TPI1P1,PFKP,GPI,PGAM1,GAPDH,PFKL,ALDOA,PPP2R1A,PFKM* |
| Reactome: degradation of the extracellular matrix | 19 | 0.005 | -2.11 | *MMP3,MMP14,MMP2,MMP9,MMP16,MMP1,MMP10* |
| Reactome: TCA cycle and respiratory electron transport | 116 | 0.005 | 2.09 | *LDHA,PDK4,SLC16A3,UQCR11,ATP5G1,FH,NDUFB2,ATP5H,NDUFS2,MDH2,COX5B,NDUFB7,PDHA1,COX6B1,CS,ATP5E,ATP5A1,COX8A,SLC16A1,UQCRQ,ETFA,LDHB,NDUFB9,ATP5D,SDHB,IDH1,ATP5B,ATP5C1,SUCLG1,COX6A1,NDUFV1,ATP5I,ATP5F1,UQCRFS1,ETFB,ATP5J,NDUFS8,COX7B,UQCRH,NDUFS6,NDUFA4,DLST,ATP5O,NDUFA11,NDUFS4,IDH3B,PDHB,UQCRC1* |
| Reactome: biological oxidations | 79 | 0.005 | 2.08 | *NNMT,GGT5,UGP2,MAOA,PAPSS2,ADH1B,PTGS1,ALDH2,CYP1B1,MAOB,CYP19A1,MGST1,GCLM,AHCY,ADH1A,MAT2A,GSTO1,TBXAS1,CYP1A1,FMO2,SLC35D1,ACSS1,CYP2U1* |
| Reactome: cytosolic tRNA aminoacylation | 24 | 0.005 | 2.01 | *PPA1,VARS,AIMP2,KARS,EPRS,DARS,LARS,FARSA,FARSB,HARS,MARS,GARS,NARS,QARS,TARS,SARS,IARS* |
| KEGG: NOD-like receptor signaling pathway | 56 | 0.005 | -1.93 | *CXCL1,CCL8,MAPK10,CASP1,MAPK8,BIRC2,BIRC3,CCL2,RIPK2,CXCL2,HSP90B1,NLRP1,IL1B,IL6,CCL7* |
| KEGG: cytokine-cytokine receptor interaction | 156 | 0.005 | -1.80 | *TSLP,CCL3,KIT,CXCL14,NGFR,ACVR2B,IL12A,TGFB3,IL18,ACVR1,IL1RAP,FIGF,IFNE,IL18R1,IL21R,CXCL6,CD40,TNFSF9,TNFSF13B,CXCL1,BMPR1A,BMPR2,CCL8,INHBE,FLT3LG,TNFRSF10B,VEGFA,VEGFC,TGFBR1,TGFB2,CXCR4,CXCL12,TGFB1,TNFRSF14,IL23A,IL7,LEPR,CSF2,TNFSF15,TNFSF10,HGF,IFNAR1,CCL2,CXCL2,CXCL3,BMP2,TNFRSF11B,IL11,IL24,IL1B,IL6,LIF,IL1A,INHBA,CCL7* |
| Reactome: metabolism of RNA | 272 | 0.005 | 2.40 | *HSPB1,RPS19,MAPK11,SNRPB,RPS5,RPSA,EIF4G1,RPS21,RPS7,ZFP36,RPLP2,RPL10A,RPS17,RPS29,RPL36,GEMIN7,RPL29,RPL26,TNKS1BP1,RPS8,RPS10,RPS15AP11,RPL13A,RPL21P134,CLNS1A,YWHAB,RPL3,RPS16,RPS6,RPS3,RPL27,RPL13,EIF4A3,RPS28,RPL37A,LSM1,RPL7A,SNRPD2,RPS12,RPL18,RPS2,FAU,RPS15,RPL34,RPS15P5,RPL14,PSMF1,RPL15,RPL23,RPS14,RPL23AP18,EXOSC7,RPS3A,PSMA7,RPS27A,RPL35A,MAPK14,RPL12,RPL35,RPL5,RPS25,ETF1,RPL30,RPL18A,RPLP0,PSMC3,PSMB4,RNPS1,RPL27A,RPL9,RPL4,PPP2R1A,PSMC5,NCBP2,RPSAP12,SMG5,SNRPE,RPS15A,RPL8,RPL10,RPS23,RPS19P3,RPL23AP42,RPL5P1,RPL19,PSMD8,RPL32,RPS4Y1,GEMIN5,SNRPG,PRKCD,PSME2,SNRPD1,NUP35,RPS13,PSMA5,EXOSC2,RPL31,WDR77,UBA52,EIF4A1,RPS26P28,AKT1,PSMB5,RPL38,RPL7,UPF3A,RPS18,PRMT5,RPS26,RPL6,RPL17,RPL22,GEMIN4,PSMB3,RPS4X,RPS26P35,PSMB9,PSMC4,RPL37,RPS20,RPS9,PSMD13,KHSRP,SEH1L,NUP50,EDC4,NUP153,POM121,RPS27,PARN,NCBP1,PABPC1* |
| Reactome: metabolism of proteins | 412 | 0.005 | 2.10 | *B3GNT5,GCNT1,TUBB6,ARSJ,VDAC1,EIF3B,EIF5A,TUBB2A,TUBB4B,ACTB,GNPNAT1,TUBA1B,RPS19,GRPEL1,TUBA1C,SEC61G,HSPD1,ST6GALNAC4,TRAM1,RPS5,RPSA,CCT3,C1GALT1C1,EIF4G1,STS,RPS21,RPS7,TIMM8B,ATP5G1,RPLP2,RPL10A,RPS17,EIF2S1,RPS29,RPL36,MPI,RPL29,RPL26,EIF5B,RPS8,RPS10,RPS15AP11,PROS1,RPL13A,RPL21P134,RPL3,RPS16,MGAT5,RPS6,RPS3,RPL27,RPL13,GALNT10,SLC25A6,RPS28,MGAT1,RPL37A,CS,TIMM13,RPL7A,TBCE,RPS12,RPL18,RPS2,ATP5A1,FAU,RPS15,RPL34,EIF4H,RPS15P5,RPL14,NOP56,RPL15,RPL23,RPS14,RPL23AP18,EIF2B5,CCT5,RPS3A,EIF3K,RPS27A,RPL35A,RFT1,RPL12,CCT7,RPL35,RPL5,RPS25,EEF1D,ETF1,RPL30,EIF3I,SRPRB,RPL18A,EIF3D,RPLP0,B3GNT4,BCS1L,KIFC3,RPL27A,RPL9,TUBA1A,RPL4,ATP5B,EEF1B2,PIGN,TIMM17A,RPSAP12,EIF3G,FBXL3,RPS15A,RPL8,PIGW,RPL10,RPS23,RPS19P3,RPL23AP42,TIMM50,ALG8,EIF3F,RPL5P1,RPL19,RPL32,CCT2,RPS4Y1,SEC61B,RPS13,RPL31,MGAT4B,CCT8,EIF2S2,UBA52,PMM2,PMPCA,EEF2,B4GALT5,EIF2S3,MLEC,ALG3,EIF4A1,EIF3A,RPS26P28,MAN1A1,TCP1,CCT6A,ALG5,TUBB4A,RPL38,RPL7,EIF2B1,PFDN6,RPS18,PIGS,TIMM22,RPS26,RPL6,RPL17,RPL22,CHCHD4,RPS4X,RPS26P35,TOMM40,TIMM8A,GCNT4,CCT4,DAD1,RPL37,RPS20* |
| Reactome: signaling by BMP | 21 | 0.006 | -2.04 | *UBE2D3,SMAD1,SKI,ACVR2B,SMURF1,SMAD4,BMPR1A,BMPR2,UBE2D1,ZFYVE16,SMAD7,BMP2,GREM2* |
| Reactome: response to elevated platelet cytosolic Ca2 | 66 | 0.007 | 1.95 | *FN1,ACTN4,CD9,A2M,SERPINE1,THBS1,WDR1,ITGB3,ACTN1,CAP1,PPIA,VCL,CFL1,TLN1,PF4,PFN1,PROS1,ALDOA,CFD,PPIAP22* |
| KEGG: hematopoietic cell lineage | 44 | 0.037 | -1.70 | *ITGA1,IL7,CD55,ITGA6,CSF2,MME,CD44,IL11,ITGA2,IL1B,IL6,IL1A* |
| KEGG: starch and sucrose metabolism | 25 | 0.062 | 1.73 | *UGP2,PYGB,GPI,HK1,PGM1,GYS1,PYGL,GBE1,MGAM,GANC* |
| KEGG: leishmania Infection | 49 | 0.114 | -1.50 | *TGFB2,ITGB1,TGFB1,JAK2,FOS,MARCKSL1,IL1B,IL1A,PTGS2* |
| Reactome: NCAM1 interactions | 28 | 0.396 | -1.17 | *CACNA1H,AGRN,COL9A3,COL1A2,COL6A2,COL6A1,PRNP,COL6A3,GFRA1* |
| **BUD+TNFα versus TNFα among cells transfected with *CEBPD* siRNA** | | | | |
| Reactome: degradation of the extracellular matrix | 19 | 0.008 | -2.22 | *MMP14,MMP9,TIMP2,FURIN,MMP2,KLKB1,MMP3,MMP16,MMP1,MMP10* |
| KEGG: NOD-like receptor signaling pathway | 55 | 0.008 | -2.19 | *MAPK3,HSP90AA1,RELA,CCL8,NFKB1,CXCL2,CCL2,MAPK13,BIRC3,NLRP1,CASP1,HSP90B1,IL6,RIPK2,CCL7,IL1B* |
| KEGG: leishmania Infection | 48 | 0.008 | -2.13 | *TGFB2,MYD88,ITGB1,MAPK3,RELA,NFKB1,IL12A,MAPK13,JUN,TGFB1,MARCKSL1,IL1A,IL1B,PTGS2* |
| KEGG: cytokine-cytokine receptor interaction | 153 | 0.008 | -2.11 | *TGFB2,TSLP,TNFSF18,VEGFB,CXCL16,TNFRSF9,IL15RA,IL21R,TNFRSF12A,CCL8,ACVR1,TNFRSF21,TNFRSF11B,CXCL2,CXCR4,CCL2,MET,TNFSF10,IL12A,IL7R,CXCL3,CXCL12,BMP2,VEGFA,TGFB1,CSF2,VEGFC,IL6,TNFSF15,IL24,CCL7,IL11,INHBA,IL1A,LIF,IL1B* |
| Reactome: extracellular matrix organization | 67 | 0.008 | -1.99 | *SERPINH1,ADAMTS14,PPIB,COL15A1,COL6A3,COL10A1,MMP14,COL6A2,MMP9,TIMP2,FURIN,COL6A1,MMP2,KLKB1,COL13A1,COL7A1,COL11A1,COL5A3,MMP3,COL12A1,MMP16,MMP1,MMP10* |
| Reactome: glucose metabolism | 57 | 0.009 | 2.01 | *UGP2,PHKA1,GYS1,PYGB,PYGL,PGM2,ENO2,GPI,PGK1,PHKA2,PGM1,ENO1,TPI1,PCK2,PFKP,AGL,ALDOC,PFKL,PYGM* |
| KEGG: hematopoietic cell lineage | 42 | 0.011 | -2.02 | *ITGA6,IL7R,ANPEP,MME,CSF2,ITGA2,IL6,CD44,IL11,IL1A,IL1B* |
| KEGG: arginine and proline metabolism | 40 | 0.012 | 1.99 | *MAOA,MAOB,ALDH2,ASL,GLUL,P4HA3,ALDH1B1,AMD1,GLS,P4HA1,ARG2,GLUD1,CPS1,P4HA2,OAT,ALDH3A2,ALDH18A1,ALDH4A1,ALDH7A1* |
| KEGG: starch and sucrose metabolism | 24 | 0.012 | 1.96 | *UGP2,GYS1,PYGB,PYGL,PGM2,GANC,GPI,PGM1,AGL,HK1,PGM2L1,PYGM* |
| Reactome: cytosolic tRNA aminoacylation | 24 | 0.023 | 1.90 | *PPA1,NARS,DARS,AIMP2,EPRS,LARS,GARS,AARS,SARS,FARSB,EEF1E1,VARS,YARS,AIMP1,TARS* |
| KEGG: glycolysis gluconeogenesis | 45 | 0.030 | 1.76 | *LDHA,ADH1B,ALDH2,PGM2,ALDH1B1,ENO2,GPI,PGK1,PGM1,ENO1,TPI1,PCK2,ALDH3B1,PFKP,ALDOC,HK1,PFKL,ADH5,DLD* |
| KEGG: ribosome | 87 | 0.037 | -1.62 | *RPS6,RPL3,RPL14,RPL31,RPL22,RPL4,RPL35A,RPL41,MRPL13,RPSA,RPS18,RPL23A,RPL36,RPL27,RPL21,RPL24,RPS4X,RPL11,RPL13A,RPS3A,RPS12,RPL38,RPL29,RPL7A,RSL24D1,RPL39,RPS27,RPL6,RPL32,RPS3,RPL37A,RPS16,RPS15,RPL30,RPS5,RPL37,RPS11,RPL8,RPL35,RPS9,RPL27A,RPS24,RPL12,RPL36AL,UBA52,RPL18,RPL13,RPS20,RPS26,RPL19,RPS2,RPLP1,RPL18A,RPL28* |
| Reactome: biological oxidations | 74 | 0.045 | 1.68 | *MAOA,NNMT,UGP2,CYP1B1,GGT5,PTGS1,ADH1B,MAOB,ALDH2,PAPSS2,CYP19A1,MGST2,MGST1,MTR,CYP7B1,GGCT,CYP39A1,GCLC,GCLM,ALDH1A1,GSTO2,CYP2U1* |
| Reactome: smooth muscle contraction | 24 | 0.045 | 1.84 | *TPM4,ACTA2,LMOD1,CALD1,MYL6,MYH11,ACTG2,SORBS1,VCL,MYLK,TPM3,MYL12B,CALM1,SORBS3,TPM2* |
| Reactome: glycolysis | 23 | 0.075 | 1.73 | *ENO2,GPI,PGK1,ENO1,TPI1,PFKP,ALDOC,PFKL,TPI1P1,PFKFB2,GAPDH,PPP2R1B,ENO3,PPP2CA,PFKFB4* |
| Reactome: NCAM1 interactions | 29 | 0.217 | 1.47 | *COL5A2,COL4A1,COL5A1,COL4A4,CACNA1G,COL4A2,ARTN,COL3A1,CACNB1,COL1A1,COL9A3* |
| Reactome: signaling by BMP | 21 | 0.238 | -1.45 | *SMURF1,SMAD7,SMURF2,ZFYVE16,BMP2,GREM2* |
| Reactome: metabolism of RNA | 271 | 0.288 | -1.18 | *PSMC2,RPL21,DDX6,RPL5P1,RPL24,SMG9,RPS14,EXOSC9,RPS4X,RPL11,AKT1,RPL13A,PSMB2,RPS3A,CNOT3,SMN1,RQCD1,RPS12,RPL38,PSMB8,RPL26P30,RPL29,RPL23AP74,PSMA5,RPL7A,PSMB10,RPL39,PSME1,SMG8,PSMC3,PSMC4,RPS27,RPL6,RPL32,RPS3,RPL37A,PSME2,NUP214,HSPA1B,PSMA3,NUP153,RPS16,RPS15,RPL30,PSMA7,RPS5,RPL23AP2,PRKCD,RPL23AP18,PATL1,RPL37,ELAVL1,RPS11,RPL8,RPL35,RPS9,PSMD8,PSMC1,PPP2R2A,PSMD1,RPL27A,AAAS,RPS24,PSMD10,RPL12,PSMD4,UBA52,RPL18,RPL13,MAPKAPK2,RPS20,RPS26,PSMB4,PSMB9,DCP1B,PAIP1,KHSRP,RPL19,PSME4,RPL7AP66,NUP88,PSMB5,RPS2,PSMD11,RPLP1,SMG6,PSMA4,PSMB1,RPL23AP42,NUP188,SMG7,RPL18A,ZFP36L1,DCP1A,HSPA8,PSMD2,PSMD3,PSMB6,NUP62,PSMB7,RPL28* |
| Reactome: response to elevated platelet cytosolic Ca2 | 64 | 0.329 | 1.24 | *FN1,THBS1,LAMP2,ITGB3,CD9,ACTN4,SRGN,PROS1,PF4,F8,SERPINE1,A2M,FIGF,STXBP3,WDR1,PRKCA,TGFB3,VCL,HABP4* |
| Reactome: 3' UTR mediated translational regulation | 124 | 0.389 | -1.16 | *RPL21,RPL5P1,RPL24,RPS14,RPS4X,RPL11,RPL13A,RPS3A,RPS12,RPL38,RPL26P30,RPL29,RPL23AP74,RPL7A,RPL39,RPS27,RPL6,RPL32,RPS3,RPL37A,RPS16,RPS15,RPL30,RPS5,RPL23AP2,RPL23AP18,RPL37,RPS11,RPL8,RPL35,RPS9,RPL27A,RPS24,EIF3H,RPL12,UBA52,RPL18,RPL13,RPS20,RPS26,RPL19,EIF3I,RPL7AP66,RPS2,RPLP1,RPL23AP42,RPL18A,RPL28* |
| Reactome: TCA cycle and respiratory electron transport | 117 | 0.731 | 0.94 | *PDK4,LDHA,NDUFS2,PDK1,SDHD,SLC16A3,SLC16A1,FH,SUCLG2,NDUFA5,DLD,CYCS,SDHC,PDK3,SLC16A8,IDH1,NDUFC2,NDUFV1,NDUFB2,ETFA,NDUFA2,DLST,ATP5H,NDUFA12,NDUFA10,NDUFV3,CS,NDUFA8,UQCR11,NDUFS3,DLAT,IDH3A,IDH3B,NDUFB10,PDHA1,PDPR,ATP5C1,COX7C,NDUFB3* |
| Reactome: formation of tubulin folding intermediates by CCT/TriC | 18 | 0.738 | 0.89 | *TUBB6,TUBB2A,TUBA1C,CCT6A,TUBA1B,CCT5* |
| Reactome: metabolism of proteins | 414 | 0.805 | -0.93 | *EIF2B2,MAN1B1,RPS16,ST6GALNAC2,EEF1D,RPS15,RPL30,SUMF2,SEC13,HSPA9,RPS5,EDEM2,RPL23AP2,RPL23AP18,CANX,RPL37,COX17,GALNT2,RPS11,TOMM22,FBXW5,RPL8,EEF1A1,RPL35,PFDN5,RPS9,SEC61B,STT3A,SEC11A,SEC24C,ARSA,RPL27A,RPS24,TOMM40,EIF3H,KIFC3,RPN1,RPL12,UBA52,ALG3,RPL18,RPL13,RPS20,EEF2,RPS26,SEC24D,GFPT2,EIF5A2,RPL19,EIF5,TUBA1A,UGGT1,RPN2,B3GNT9,EIF3I,PIGO,MCFD2,GGCX,RPL7AP66,PREB,RPS2,TOMM7,TUSC3,PIGU,RPLP1,SLC25A13,PIGT,PFDN2,ST6GAL1,ARSI,PIGQ,XRN2,SLC25A6,PFDN1,RPL23AP42,KIF13A,DPAGT1,DDOST,ACO2,GALNT6,ST3GAL1,PRKCSH,RPL18A,ARSG,B3GNT7,USP11,GANAB,FURIN,PDIA3,SSR2,ST3GAL2,SPHK1,CALR,PLAUR,RPL28,SEMA6D* |

# Table E5. Ontological categories enriched within gene co-expression groups.

Ontological categories that were significant (q-value <0.05) in each of the three gene co-expression groups (i.e., groups 1, 2 and 3) are shown, along with the number of genes within the co-expression group were part of the ontological category (Gene count), the names of these genes (Genes), the percentage that the Gene count represents of the total number of genes in the co-expression group (Percentage of Gene count), and the corresponding q-value.

| **Ontological category** | **Gene count** | **Percentage of**  **Gene count** | **q-value** | **Genes** |
| --- | --- | --- | --- | --- |
| **Gene co-expression group 1 associated with *CEBPD* knockdown status (N = 197)** | | | |  |
| KEGG: regulation of actin cytoskeleton | 10 | 5.1 | 3.10E-02 | *ACTN1, CYFIP2, FGF18, FGFR1, FGFR4, ITGB8, MYH9, PDGFRA, SSH2, TIAM2* |
| **Gene co-expression group 2 associated with TNFα exposure status (N = 152)** | | | | |
| Reactome: cytokine signaling in immune system | 13 | 8.6 | 2.70E-05 | *DDX58, GBP5, ICAM1, IFITM3, IL1RN, IL7R, IRAK2, MX1, OAS1, OAS2, OAS3, STAT1, USP18* |
| Reactome: interferon signaling | 10 | 6.6 | 7.30E-05 | *DDX58, GBP5, ICAM1, IFITM3, MX1, OAS1, OAS2, OAS3, STAT1, USP18* |
| Reactome: interferon alpha beta signaling | 7 | 4.6 | 1.80E-04 | *IFITM3, MX1, OAS1, OAS2, OAS3, STAT1, USP18* |
| Reactome: immune system | 19 | 12.5 | 4.30E-04 | *BIRC3, C1S, C3, DDX58, GBP5, ICAM1, IFITM3, IKBKE, IL1RN, IL7R, IRAK2, MX1, OAS1, OAS2, OAS3, PIK3AP1, SAA1, STAT1, USP18* |
| Reactome: interferon gamma signaling | 6 | 3.9 | 3.90E-03 | *GBP5, ICAM1, OAS1, OAS2, OAS3, STAT1* |
| KEGG: JAK-STAT signaling pathway | 7 | 4.6 | 2.00E-02 | *IL10RB, IL13RA2, IL15RA, IL7R, LEP, STAT1, STAT4* |
| KEGG: cytokine-cytokine receptor interaction | 8 | 5.3 | 4.20E-02 | *CCL13, CCL5, CXCL1, IL10RB, IL15RA, IL7R, LEP, TNFSF10* |
| **Gene co-expression group 3 associated with both *CEBPD* knockdown and TNFα exposure status (N = 290)** | | | | |
| Reactome: translation | 15 | 5.2 | 1.10E-05 | *EEF1A1, EEF1D, EIF4B, PABPC1, RPL10A, RPL12, RPL13, RPL13A, RPL18A, RPL23AP18, RPL23AP42, RPL3, RPL4, RPS3, RPS8* |
| Reactome: peptide chain elongation | 12 | 4.1 | 1.70E-05 | *EEF1A1, RPL10A, RPL12, RPL13, RPL13A, RPL18A, RPL23AP18, RPL23AP42, RPL3, RPL4, RPS3, RPS8* |
| Reactome: 3' UTR mediated translational regulation | 13 | 4.5 | 1.70E-05 | *EIF4B, PABPC1, RPL10A, RPL12, RPL13, RPL13A, RPL18A, RPL23AP18, RPL23AP42, RPL3, RPL4, RPS3, RPS8* |
| Reactome: nonsense mediated decay enhanced by the exon junction complex | 12 | 4.1 | 7.70E-05 | *PABPC1, RPL10A, RPL12, RPL13, RPL13A, RPL18A, RPL23AP18, RPL23AP42, RPL3, RPL4, RPS3, RPS8* |
| Reactome: influenza viral RNA transcription and replication | 11 | 3.8 | 3.50E-04 | *RPL10A, RPL12, RPL13, RPL13A, RPL18A, RPL23AP18, RPL23AP42, RPL3, RPL4, RPS3, RPS8* |
| Reactome: SRP-dependent cotranslational protein targeting to membrane | 11 | 3.8 | 4.80E-04 | *RPL10A, RPL12, RPL13, RPL13A, RPL18A, RPL23AP18, RPL23AP42, RPL3, RPL4, RPS3, RPS8* |
| KEGG: ribosome | 9 | 3.1 | 1.00E-03 | *RPL10A, RPL12, RPL13, RPL13A, RPL18A, RPL3, RPL4, RPS3, RPS8* |
| Reactome: metabolism of mRNA | 13 | 4.5 | 1.80E-03 | *EIF4B, PABPC1, RPL10A, RPL12, RPL13, RPL13A, RPL18A, RPL23AP18, RPL23AP42, RPL3, RPL4, RPS3, RPS8* |
| Reactome: influenza life cycle | 11 | 3.8 | 1.80E-03 | *RPL10A, RPL12, RPL13, RPL13A, RPL18A, RPL23AP18, RPL23AP42, RPL3, RPL4, RPS3, RPS8* |
| Reactome: metabolism of RNA | 13 | 4.5 | 8.80E-03 | *EIF4B, PABPC1, RPL10A, RPL12, RPL13, RPL13A, RPL18A, RPL23AP18, RPL23AP42, RPL3, RPL4, RPS3, RPS8* |
| KEGG: JAK-STAT signaling pathway | 9 | 3.1 | 1.00E-02 | *CCND3, IL24, IL6R, LIF, PIM1, SOCS1, SOCS2, SOCS3, SPRY4* |

# Table E6. RNA-Seq differential expression results for *IL6R*, *SOCS3*, *SOCS1*, and *SOCS2* across the 10 comparisons made.

NT: non-targeting.

| **Gene Symbol** | **Log_2_ Fold Change** | **Q-value** | **Mean Normalized Counts** | |
| --- | --- | --- | --- | --- |
|  |  |  | **NT siRNA** | ***CEBPD* siRNA** |
| ***CEBPD* siRNA versus NT siRNA under control exposure** | | | | |
| *IL6R* | -1.28 | 6.30E-18 | 397 | 164 |
| *SOCS3* | -0.83 | 2.30E-11 | 934 | 524 |
| *SOCS1* | -0.72 | 3.00E-02 | 102 | 60 |
| *SOCS2* | 0.36 | 1.70E-01 | 185 | 237 |
| ***CEBPD* siRNA versus NT siRNA under budesonide exposure** | | | | |
| *IL6R* | -1.29 | 4.70E-12 | 450 | 188 |
| *SOCS3* | -0.98 | 6.60E-12 | 910 | 477 |
| *SOCS1* | -0.6 | 9.60E-02 | 145 | 110 |
| *SOCS2* | 0.47 | 2.60E-01 | 132 | 183 |
| ***CEBPD* siRNA versus NT siRNA under TNFα exposure** | | | | |
| *IL6R* | -0.94 | 4.10E-06 | 178 | 92 |
| *SOCS3* | -0.13 | 7.70E-01 | 492 | 443 |
| *SOCS1* | -0.66 | 1.50E-01 | 80 | 49 |
| *SOCS2* | 0.37 | 2.10E-02 | 379 | 487 |
| ***CEBPD* siRNA versus NT siRNA under under budesonide +TNFα exposure** | | | | |
| *IL6R* | -0.96 | 6.10E-05 | 179 | 94 |
| *SOCS3* | -0.26 | 3.40E-01 | 461 | 387 |
| *SOCS1* | -0.86 | 2.50E-03 | 98 | 53 |
| *SOCS2* | 0.93 | 2.30E-06 | 243 | 474 |
|  |  |  |  |  |
| **Gene Name** | **Log_2_ Fold Change** | **Q-value** | **Mean Normalized Counts** | |
|  |  |  | **No Budesonide** | **Budesonide** |
| **Budesonide versus control in cells transfected with NT siRNA** | | | | |
| *IL6R* | 0.19 | 9.40E-01 | 397 | 450 |
| *SOCS3* | -0.05 | 1.00E+00 | 934 | 910 |
| *SOCS1* | 0.5 | 2.30E-01 | 102 | 145 |
| *SOCS2* | -0.51 | 8.30E-02 | 185 | 132 |
| **Budesonide versus control in cells transfected with *CEBPD* siRNA** | | | | |
| *IL6R* | 0.16 | 1.00E+00 | 164 | 188 |
| *SOCS3* | -0.14 | 1.00E+00 | 524 | 477 |
| *SOCS1* | 0.66 | 2.70E-01 | 60 | 110 |
| *SOCS2* | -0.31 | 1.00E+00 | 237 | 183 |
| **Budesonide + TNFα versus TNFα in cells transfected with NT siRNA** | | | | |
| *IL6R* | 0.05 | 1.00E+00 | 178 | 179 |
| *SOCS3* | -0.05 | 1.00E+00 | 492 | 461 |
| *SOCS1* | 0.33 | 9.50E-01 | 80 | 98 |
| *SOCS2* | -0.61 | 6.10E-03 | 379 | 243 |
| **Budesonide + TNFα versus TNFα in cells transfected with *CEBPD* siRNA** | | | | |
| *IL6R* | 0.02 | 1.00E+00 | 92 | 94 |
| *SOCS3* | -0.18 | 9.30E-01 | 443 | 387 |
| *SOCS1* | 0.12 | 1.00E+00 | 49 | 53 |
| *SOCS2* | -0.05 | 1.00E+00 | 487 | 474 |
|  |  |  |  |  |
| **Gene Name** | **Log_2_ Fold Change** | **Q-value** | **Mean Normalized Counts** | |
|  |  |  | **No TNFα** | **TNFα** |
| **TNFα versus control in cells transfected with NT siRNA** | | | | |
| *IL6R* | -1.22 | 9.50E-07 | 397 | 178 |
| *SOCS3* | -1.01 | 8.40E-09 | 934 | 492 |
| *SOCS1* | -0.34 | 5.20E-01 | 102 | 80 |
| *SOCS2* | 1.01 | 1.30E-07 | 185 | 379 |
| **TNFα versus control in cells transfected with *CEBPD* siRNA** | | | | |
| *IL6R* | -0.86 | 1.10E-03 | 164 | 92 |
| *SOCS3* | -0.3 | 5.00E-01 | 524 | 443 |
| *SOCS1* | -0.28 | 8.10E-01 | 60 | 49 |
| *SOCS2* | 1.03 | 8.40E-06 | 237 | 487 |

# Figure E1. Sample quality control prior to RNA-Seq via RT-qPCR of *CEBPD* and *CXCL8*.

A) *CEBPD* and B) *CXCL8* mRNA expression in ASM cells from four non-asthma donors transfected with NT siRNA or *CEBPD* siRNA, and exposed to control, BUD, TNFα, and BUD+TNFα as measured by RT-qPCR. Expression levels were measured in triplicates per condition per donor. Barplots are of height equivalent to the mean across the three technical replicates, and error bars represent corresponding standard errors (SEs). BUD: budesonide; NT: non-targeting. ^#^sample was not included in RNA-Seq experiment.


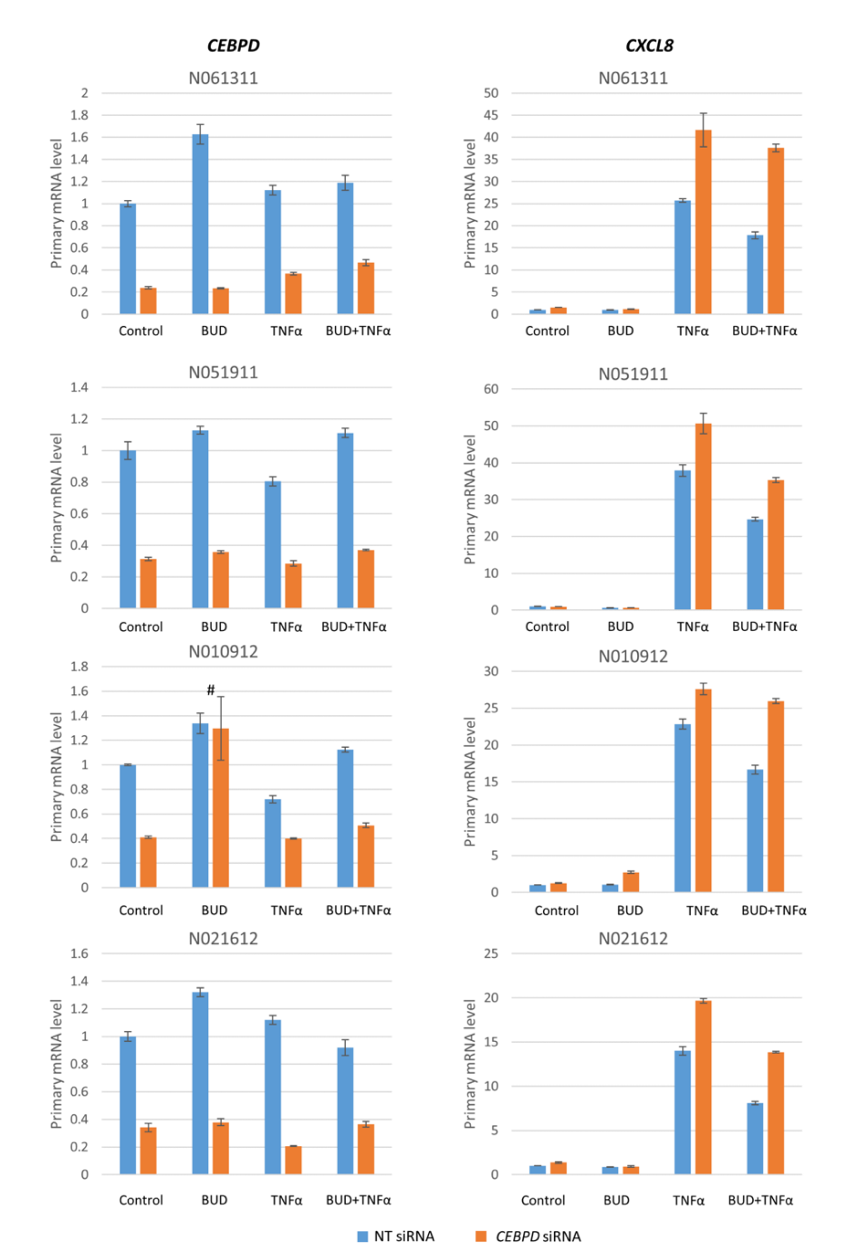


# Figure E2. RNA-Seq data quality control.

A) Estimated read coverage across transcripts for each sample. Position and coverage are normalized by adjusting for transcript lengths and total number of reads per sample. B) Gene expression of the housekeeping genes *GABARAP* and *RPL19* by control, BUD, TNFα, and BUD+TNFα exposures in NT siRNA cells. *CEBPD* knockdown was confirmed by C) reduced normalized counts in RNA-Seq in ASM cells transfected with NT siRNA and *CEBPD* siRNA of four exposures. N=3-4 donors per condition. BUD: budesonide; NT: non-targeting.


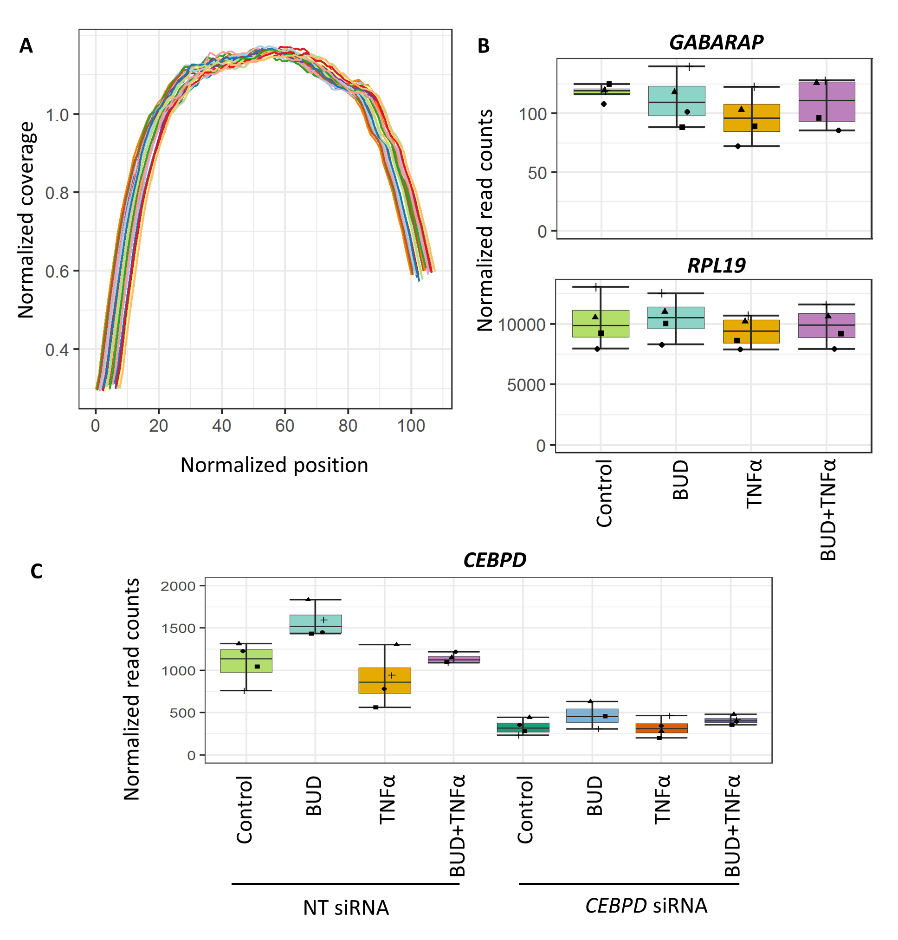


# Figure E3. Significant gene set enrichment analysis categories corresponding to the *CEBPD* siRNA versus NT siRNA comparisons.

Ontological categories with a q-value <0.05 and an absolute normalized enrichment score (NES) >2 in any of the four exposures are shown. BUD: budesonide; NT: non-targeting.

**
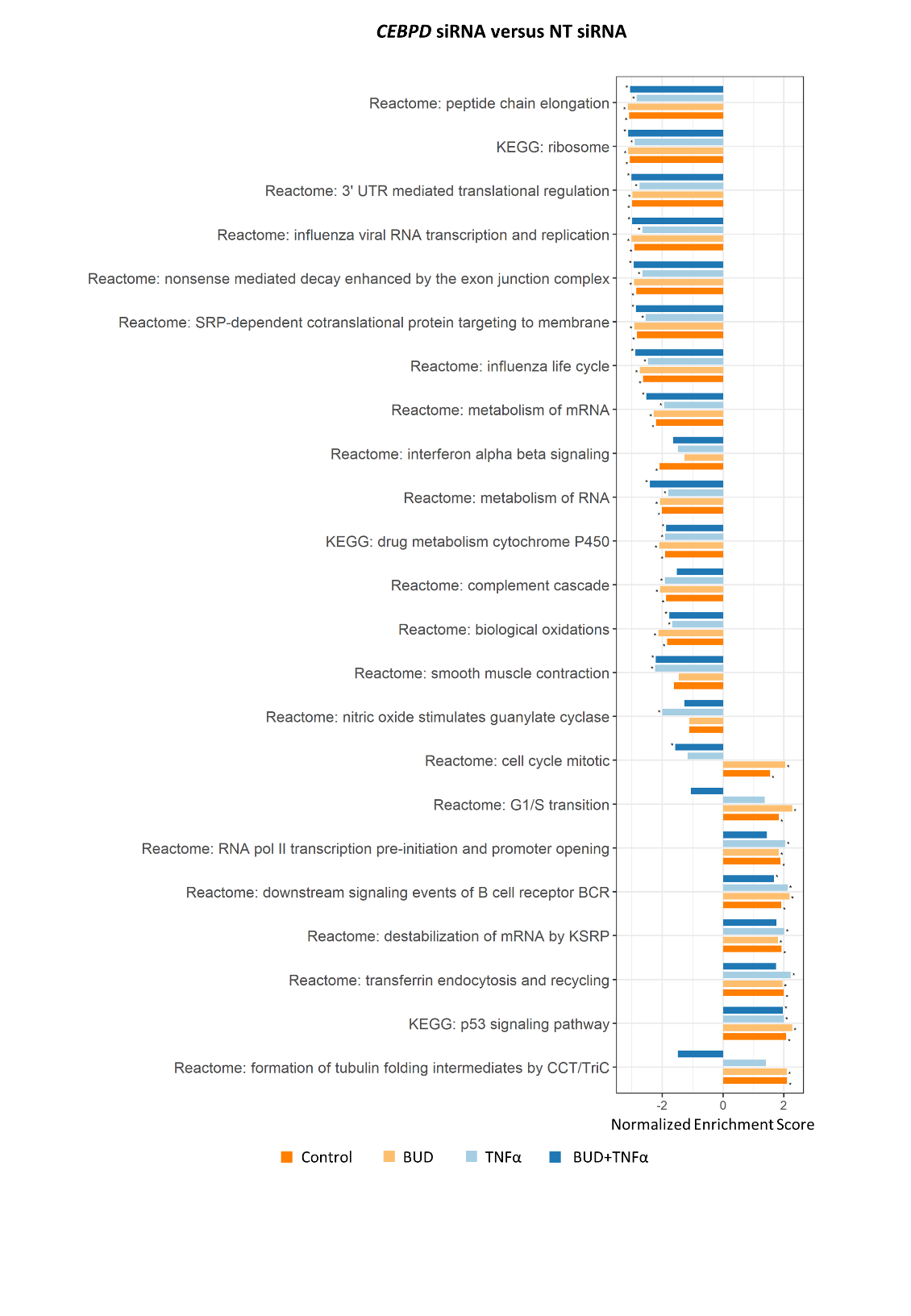
**

# Figure E4. Overall RNA-Seq results for each exposure were generally consistent with *CEBPD* knockdown.

Log_2_-fold changes of differential expression results for A) TNFα versus control, B) BUD versus control and C) BUD+TNFα versus TNFα within the NT siRNA and *CEBPD* siRNA groups show overall consistency of direction of effect with knockdown. BUD: budesonide; NT: non-targeting.


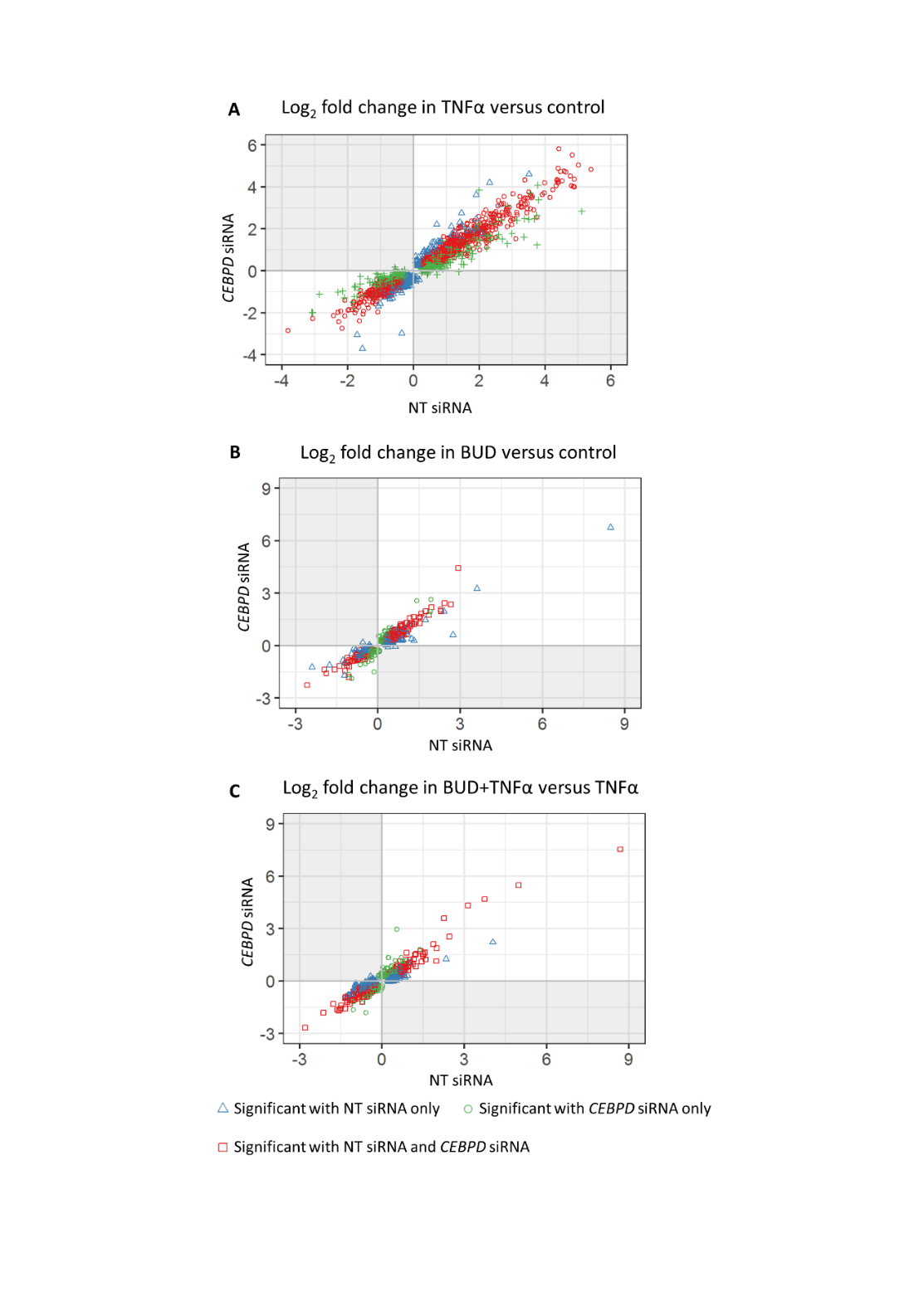


# Figure E5. Significant gene set enrichment analysis categories corresponding to the TNFα versus control comparisons.

Ontological categories with a q-value <0.05 and an absolute normalized enrichment score (NES) >2 within transfection status conditions (i.e., NT siRNA and *CEBPD* siRNA) are shown. NT: non-targeting.

**
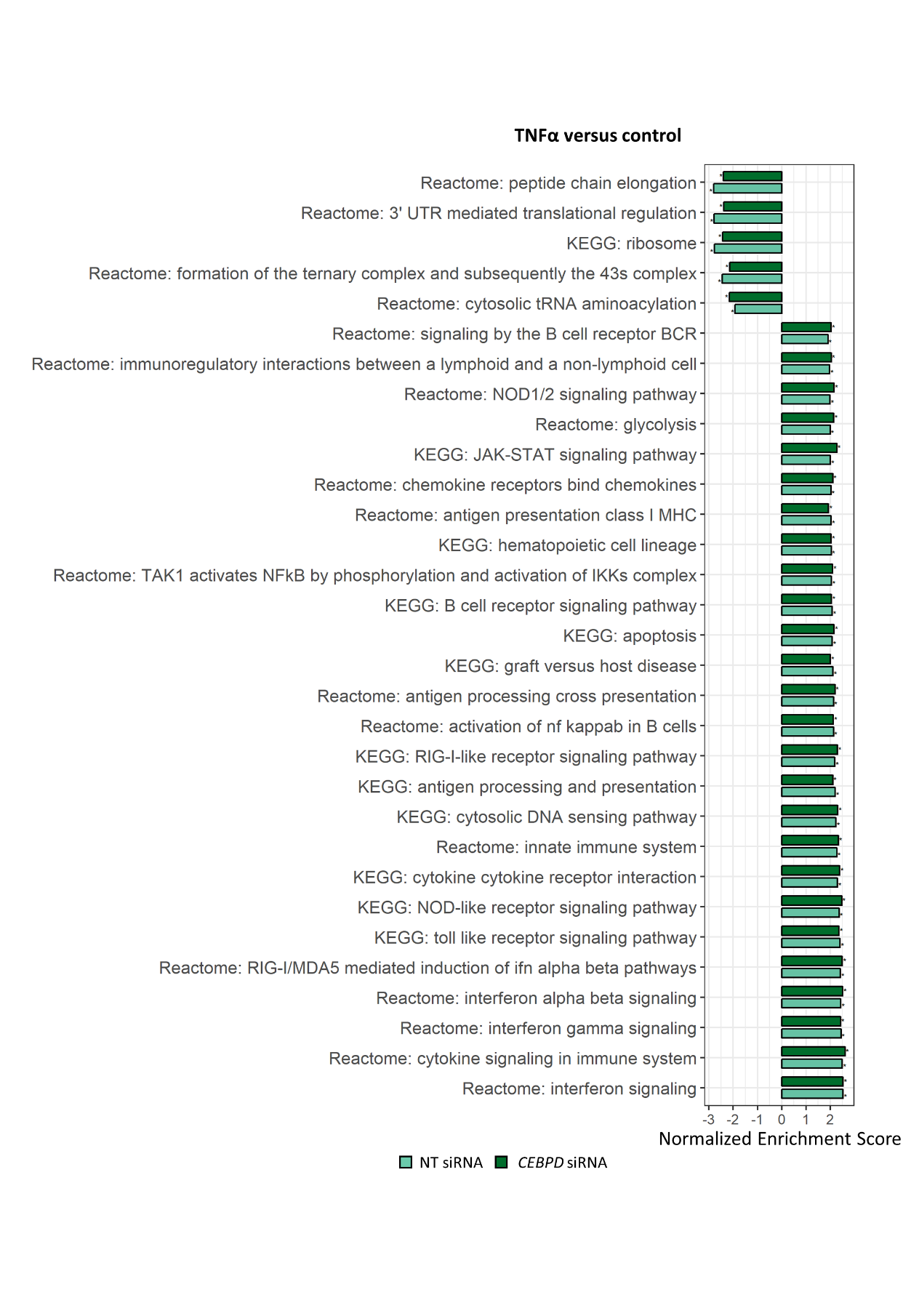
**

# Figure E6. TNFα-responsive genes whose expression changed with *CEBPD* knockdown selected from two significantly changed ontological categories.

Normalized read counts for *ITGA1*, *MYL9*, *GUCY1B3* and *MRVI1* in NT siRNA and *CEBPD* siRNA cells exposed to 1) control, 2) BUD, 3) TNFα, and 4) BUD+TNFα exposure. *ITGA1* and *MYL9* drove the *smooth muscle contraction* category over-representation, while *GUCY1B3* and *MRVI1* drove the *nitric oxide stimulates guanylate cyclase* over-representation. N=3-4 donors per condition. BUD: budesonide; NT: non-targeting.


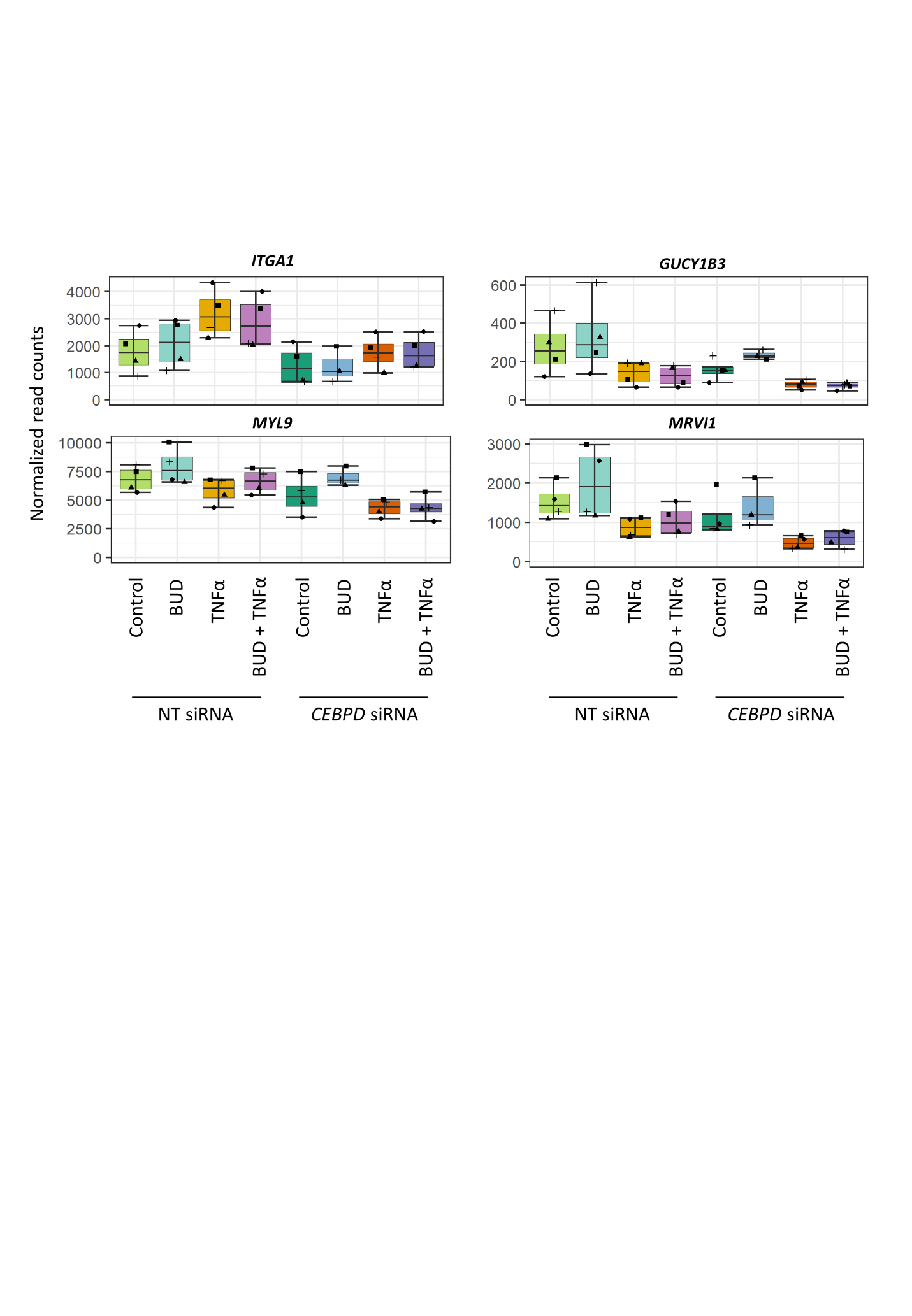


# Figure E7. Significant gene set enrichment analysis categories corresponding to budesonide-responsive genes.

Ontological categories with a q-value <0.05 and an absolute normalized enrichment score (NES) >2 in any of the four comparisons representing BUD response are shown. BUD: budesonide; NT: non-targeting.


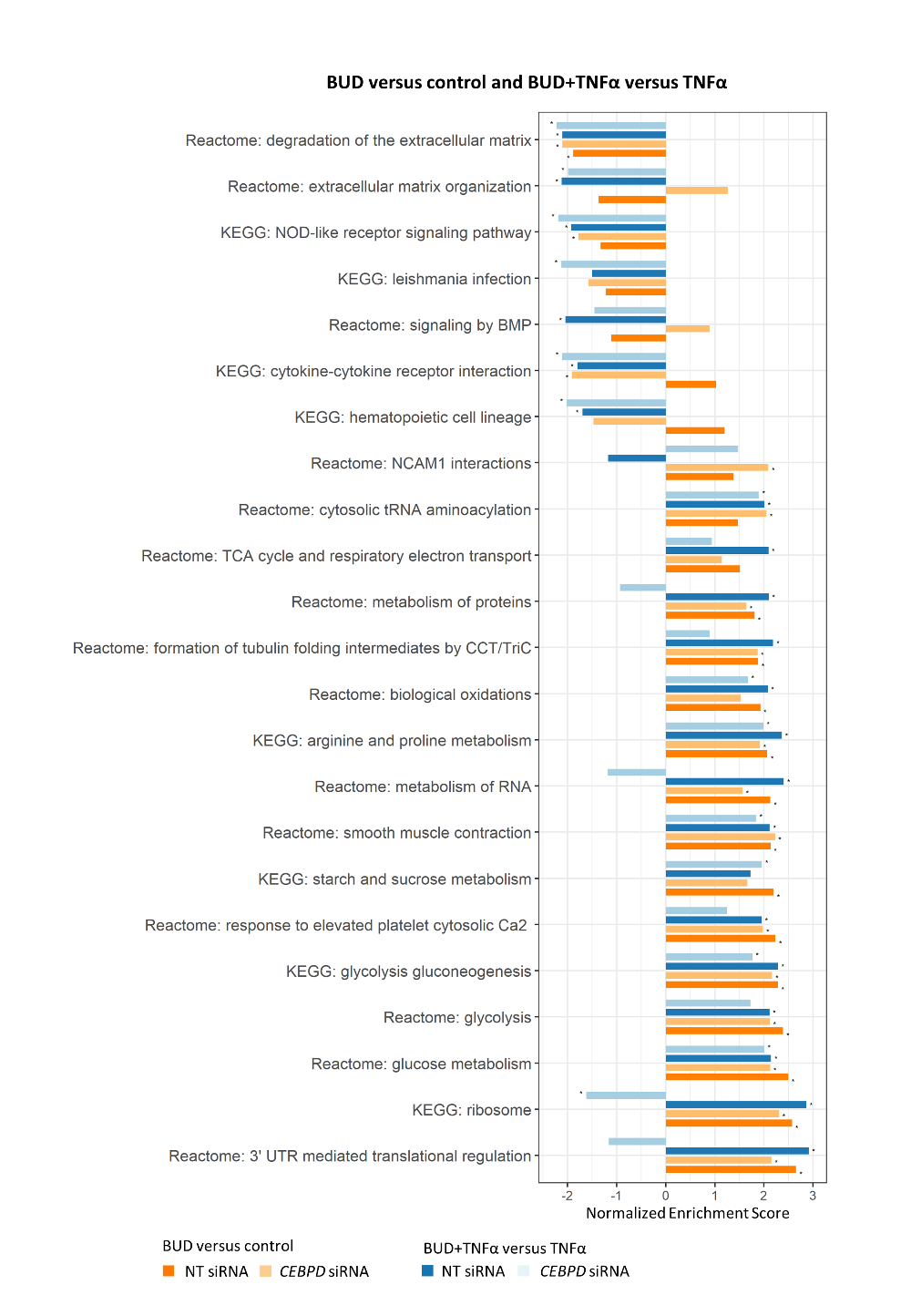


# Figure E8. Selection of soft-thresholding power (β) for weighted gene co-expression network analysis.

The soft-thresholding power (β) was selected to be 18 because A) it was the smallest power parameter that led to the network approximately fitting a scale-free topology with an *R^2^* >0.9, and B) the number of connections in the network did not significantly decrease when the power parameter reached 18.


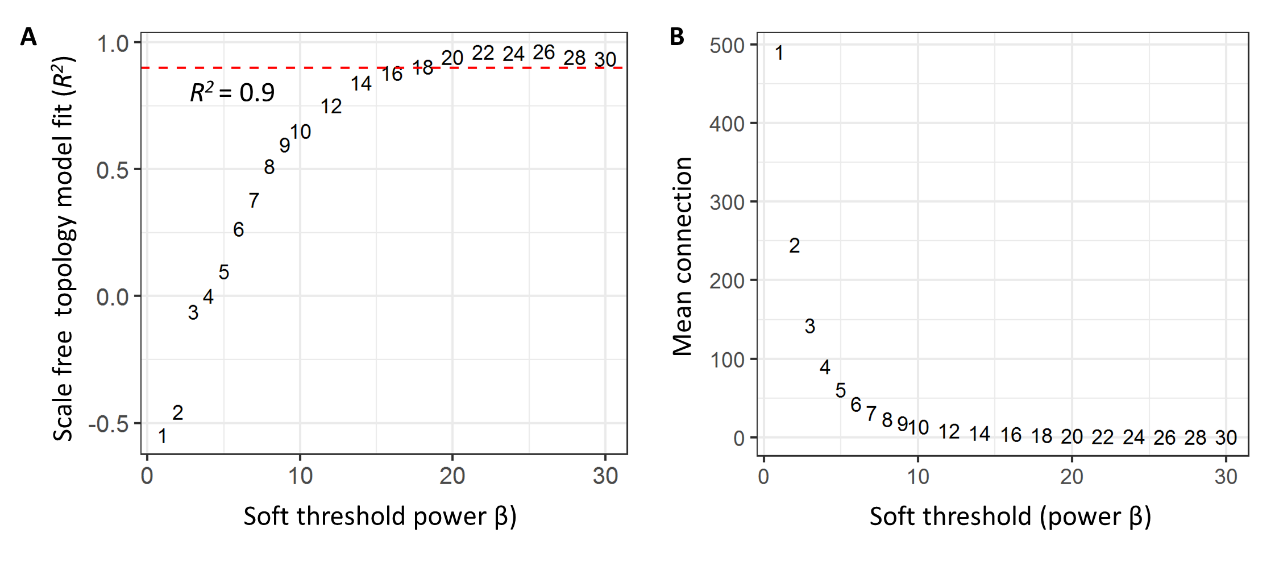


# Figure E9. Correlations between gene co-expression groups and phenotypes.

The correlation coefficients between *eigengenes* of each gene co-expression group and each phenotype (i.e., exposure condition and donor) indicated that Groups 1-3 were the only ones not highly related to a donor. Each box contains the correlation coefficient (top) and corresponding p-value (bottom) for a given group versus phenotype pair. BUD: budesonide; NT: non-targeting.


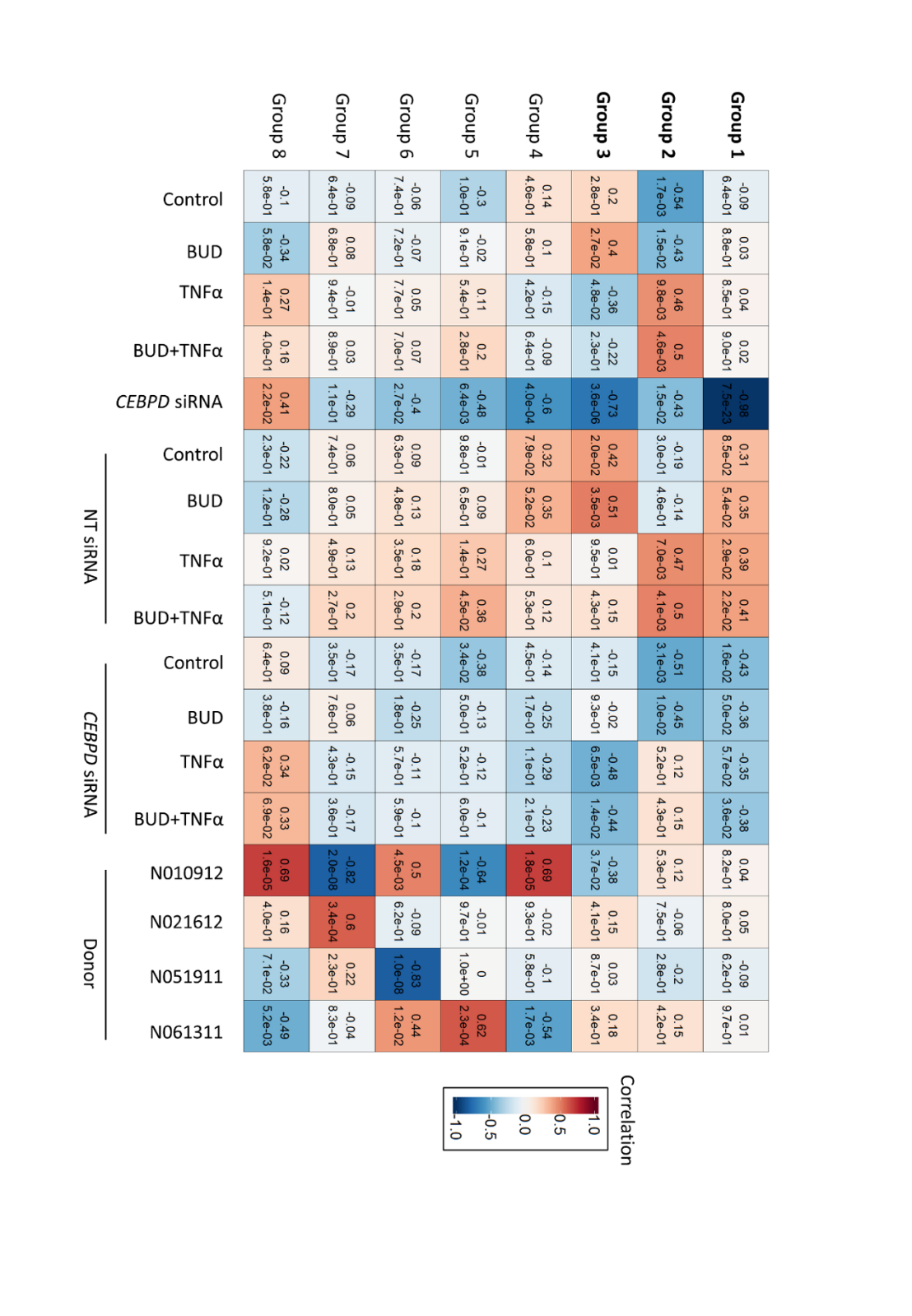


# Figure E10. CEBPD-binding sites near select IL-6 signaling pathway genes.

CEBPD-binding sites in HepG2 (green) and K562 (purple) cells near the transcription start sites (TSS) of *IL6R*, *SOCS3*, *SOCS1*, and *SOCS2* are visualized using UCSC Genome Browser tracks. The arrow points to the transcription start site of each gene.


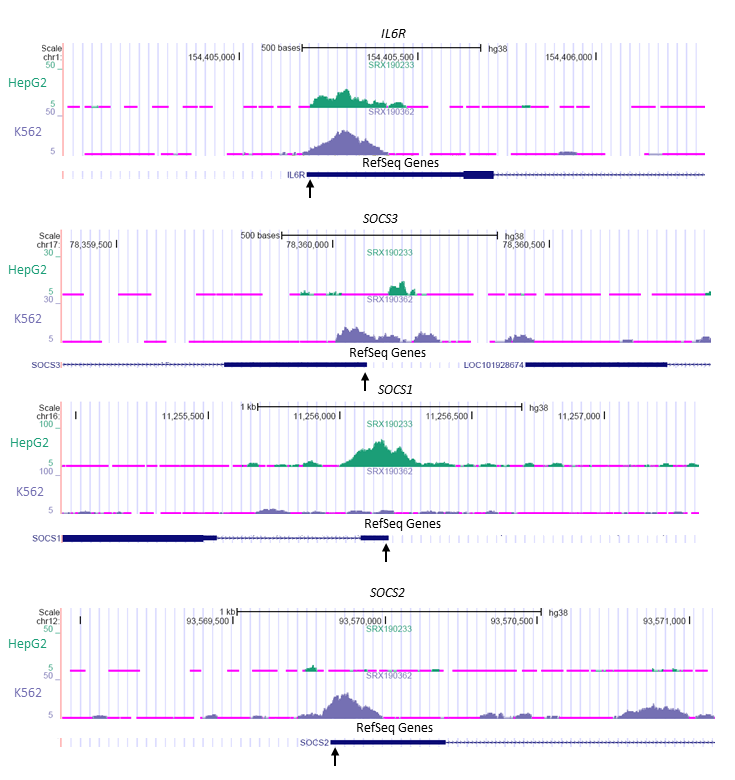


# Figure E11. Full representative immunoblots and CEBPD/Tubulin expression levels showing adequacy of CEBPD knockdown.

A**)** Representative immunoblots showing all experimental conditions tested for immunoblotting experiments that were rearranged to create Figures 3A-B. B) CEBPD expression levels (normalized to tubulin and NT siRNA under control condition) were significantly decreased in *CEBPD* knockdown cells under exposures of IL-6, BUD, and BUD+IL-6. The ratios of signals were visualized as barplots of height equivalent to the mean across donors and error bars represent standard errors (SEs) across replicates. N=6 donors per condition. BUD: budesonide; NT: non-targeting; pSTAT3: phosphorylated STAT3. *p-value <0.05.

**
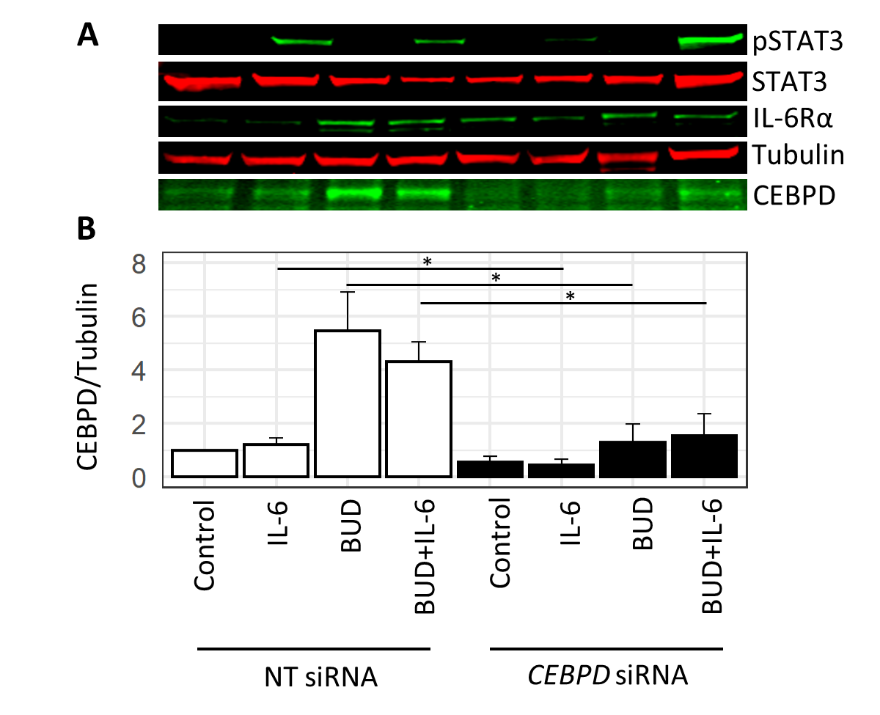
**

**References**

1. Kan M, Shumyatcher M, Diwadkar A, Soliman G, Himes BE. Integration of Transcriptomic Data Identifies Global and Cell-Specific Asthma-Related Gene Expression Signatures. AMIA Annu Symp Proc. 2018;2018:1338–47.

2. Bolger AM, Lohse M, Usadel B. Trimmomatic: a flexible trimmer for Illumina sequence data. Bioinformatics. 2014;

3. Andrews S. FastQC A Quality Control tool for High Throughput Sequence Data.

4. Dobin A, Davis CA, Schlesinger F, Drenkow J, Zaleski C, Jha S, et al. STAR: ultrafast universal RNA-seq aligner. Bioinformatics. 2013;29:15–21.

5. Barnett DW, Garrison EK, Quinlan AR, Stromberg MP, Marth GT. BamTools: a C++ API and toolkit for analyzing and managing BAM files. Bioinformatics. 2011;27:1691–2.

6. Anders S, Pyl PT, Huber W. HTSeq--a Python framework to work with high-throughput sequencing data. Bioinformatics. 2015;31:166–9.

7. Love MI, Huber W, Anders S. Moderated estimation of fold change and dispersion for RNA-seq data with DESeq2. Genome Biol. 2014;15:550.

8. Durinck S, Moreau Y, Kasprzyk A, Davis S, De Moor B, Brazma A, et al. BioMart and Bioconductor: a powerful link between biological databases and microarray data analysis. Bioinformatics. 2005;21:3439–40.

9. Langfelder P, Zhang B, Horvath S. Defining clusters from a hierarchical cluster tree: the Dynamic Tree Cut package for R. Bioinformatics. 2008;24:719–20.

10. Zhang B, Horvath S. A General Framework for Weighted Gene Co-Expression Network Analysis. Statistical Applications in Genetics and Molecular Biology [Internet]. 2005 [cited 2019 Sep 23];4. Available from: https://www.degruyter.com/view/j/sagmb.2005.4.issue-1/sagmb.2005.4.1.1128/sagmb.2005.4.1.1128.xml

11. Sergushichev A. An algorithm for fast preranked gene set enrichment analysis using cumulative statistic calculation. bioRxiv. 2016;

12. Hosack DA, Dennis G, Sherman BT, Lane HC, Lempicki RA. Identifying biological themes within lists of genes with EASE. Genome Biol. 2003;4:R70.

13. Huang DW, Sherman BT, Lempicki RA. Systematic and integrative analysis of large gene lists using DAVID bioinformatics resources. Nat Protoc. 2009;4:44–57.

14. Gertz J, Savic D, Varley KE, Partridge EC, Safi A, Jain P, et al. Distinct properties of cell-type-specific and shared transcription factor binding sites. Mol Cell. 2013;52:25–36.

15. Diwadkar AR, Kan M, Himes BE. Facilitating Analysis of Publicly Available ChIP-Seq Data for Integrative Studies. AMIA Annu Symp Proc. 2019;2019:371–9.

16. Li H, Durbin R. Fast and accurate short read alignment with Burrows-Wheeler transform. Bioinformatics. 2009;25:1754–60.

17. Zhang Y, Liu T, Meyer CA, Eeckhoute J, Johnson DS, Bernstein BE, et al. Model-based analysis of ChIP-Seq (MACS). Genome Biol. 2008;9:R137.

18. Panettieri RA, Murray RK, DePalo LR, Yadvish PA, Kotlikoff MI. A human airway smooth muscle cell line that retains physiological responsiveness. American Journal of Physiology: Cell Physiology. 1989;256:C329–35.

19. Cooper PR, Mesaros AC, Zhang J, Christmas P, Stark CM, Douaidy K, et al. 20-HETE mediates ozone-induced, neutrophil-independent airway hyper-responsiveness in mice. PLoS ONE. 2010;5:e10235.

20. Butler JP, Tolić-Nørrelykke IM, Fabry B, Fredberg JJ. Traction fields, moments, and strain energy that cells exert on their surroundings. Am J Physiol, Cell Physiol. 2002;282:C595-605.
